# End User Needs and Perspectives for a Digital Opioid Safety Tool in Adolescents and Young Adults With Inflammatory Bowel Disease: A Qualitative Human-Centered Design Study

**DOI:** 10.2196/92202

**Published:** 2026-07-31

**Authors:** Maria Younan, Amanda Acquaire, Lauren Castro, Deysi Paniagua-Perez, Devyani Dharanipragada, Stephen B Hanauer, Jane L Holl, Mehul V Raval, Andrew B L Berry, Salva N Balbale

**Affiliations:** 1 Feinberg School of Medicine, Northwestern University, 633 N St Clair Street, 20th Floor, Chicago, IL, United States, 1 8477545967; 2 Central Michigan University College of Medicine, Saginaw, MI, United States; 3 McCormick School of Engineering, Northwestern University, Evanston, IL, United States; 4 School of Medicine, University of Chicago, Chicago, IL, United States; 5 Lurie Children's Hospital, Chicago, IL, United States; 6 Edward Hines, Jr VA Hospital, Hines, IL, United States

**Keywords:** human-centered design, digital health, inflammatory bowel disease, Crohn's disease, ulcerative colitis, opioid analgesics, adolescents and young adults, pain management, patient-clinician communication

## Abstract

**Background:**

Chronic opioid use, a key predictor of opioid overdose, is common among adolescents and young adults (AYA) with inflammatory bowel diseases (IBD), underscoring a need for tailored interventions to monitor risk for opioid-related harm. Prior research highlights the need to engage both AYA patients with IBD and IBD-focused clinicians in the development of pain management and opioid safety interventions. Human-centered design offers a promising approach to address this gap by directly engaging patients and clinicians in cocreating solutions.

**Objective:**

We aimed to explore and identify AYA patient and clinician perspectives to inform the design and development of a digital opioid safety tool.

**Methods:**

Using convenience sampling, we conducted semistructured interviews with AYA patients with IBD and IBD-focused clinicians (gastroenterologists, surgeons, and nurses) in July-August 2023 in person or by video-based conferencing. Interviews explored patient experiences with pain management, opioid use, and transitions to adult care, as well as clinician experiences in monitoring pain and prescribing opioids. A co-design workshop, following the interviews, brought patient and clinician participants together to reflect as a group on the unique challenges of managing pain in IBD care, and consider potential creative solutions to enhance pain management safety. The workshop was facilitated in person in September 2023 and included 7 patients and 8 clinicians who previously participated in individual interviews. Data were audio recorded, transcribed, and thematically analyzed using an inductive approach to identify themes.

**Results:**

Twenty participants (7 AYA patients and 13 clinicians) contributed to the study. Thematic analysis generated three domains of needs that a digital opioid safety tool should address: (1) Intersecting Needs (ie, relevant at the patient and clinician and/or health system levels), (2) Patient-Level Needs (ie, relevant at the patient level only), and (3) Clinician- and/or Health System–Level Needs (ie, relevant at the clinician and/or health system levels only). Intersecting needs included integrating opioid safety interventions into multidisciplinary chronic care, supporting AYA transitions to independence, and acknowledging individual patient differences. Patient-level needs included assessing lived experiences of pain routinely, setting clear expectations about pain management, and connecting patients with safe nonopioid alternatives. Clinician- and/or health system–level needs included accounting for pain management received outside the IBD clinic; addressing gaps in information, education, and resources regarding opioid risk or pain management; and coordinating safety efforts across clinical teams.

**Conclusions:**

Incorporating these insights into the development of a digital opioid safety tool may enhance alignment between patient and clinician expectations on pain management and opioid use. This study underscores the value of human-centered design in developing digital opioid safety tools that are practical, patient-focused, and effectively integrated into clinical workflows. Findings may guide future intervention design, prototyping, and testing with continued engagement of AYA patients with IBD and clinicians.

## Introduction

The opioid epidemic in the United States continues to be a significant public health problem with disproportionate impact on adolescents and young adults (AYA). Evidence indicates that 2 of every 3 adults treated for opioid use disorder initiated opioid use before the age of 25 years [1]. Today, opioid overdoses are a leading cause of death among AYA, frequently resulting from chronic, or long-term, opioid use [2]. AYA with opioid use disorder have lower rates of engaging with, and retaining, treatment [3]. AYA [4] may also face age-specific barriers, including developmental vulnerability, stigma, and inadequate treatment system capacity [5]. In addition to its association with overdose and other serious harms, chronic opioid use is also ineffective as a long-term pain management strategy [6-8]. This concern is particularly salient for AYA living with painful, chronic illnesses such as inflammatory bowel diseases (IBDs) [9,10], including Crohn’s disease and ulcerative colitis, characterized by relapsing inflammation of the gastrointestinal (GI) tract [11]. The prevalence of IBD has risen significantly over the past decade, with notable increases among individuals aged 10-17 years in the United States [2,12]. Pain is a commonly reported symptom across the IBD continuum [13], often leading to short-term opioid prescriptions for acute pain management. However, evidence suggests that approximately 20% of AYA with IBD transition to chronic opioid use [14], placing this population at high risk of opioid overdoses or opioid use disorder, poorer disease-related outcomes [15], greater health care use [2], and reduced quality of life [16,17].

The high prevalence of opioid use among AYA with IBD creates an urgent need for targeted interventions and safer, more effective strategies to mitigate opioid-related harm [14]. Existing literature highlights the importance of engaging AYA with IBD and their clinicians as active contributors in developing opioid safety and pain management solutions [18-21]. This is especially important, given that a significant proportion of young patients reports that they experience persistent pain and, in parallel, receive limited guidance or resources to address pain safely [18]. Prior studies also show that opioid use in this patient population is closely linked to IBD recurrence and comorbid conditions, such as joint pains, highlighting a need to evaluate individualized risk factors for AYA with IBD [14]. Despite these challenges, there remains a gap in available tools or strategies that enable AYA with IBD and their clinicians to monitor opioid exposure at the point of care or assess risk of progression to chronic use [22,23].

Human-centered design (HCD) offers a useful approach to develop evidence-based, scalable opioid safety tools tailored to AYA with IBD [24]. With an emphasis on empathy and engagement with end users to generate tailored, contextually grounded solutions, HCD has been increasingly used across health care settings, including pediatric care [24-28]. HCD centers the lived experiences, preferences, and challenges of patients and clinicians, ensuring that resulting interventions are relevant, acceptable, and effective. Application of HCD is a relatively new concept in IBD care. To date, it has been used to develop clinician decision support tools to address anemia among patients with IBD [29], tailor care coordination according to IBD patient-centric values and needs [30], and coproduce patient-centered educational resources [25,31]. Beyond IBD, HCD has been applied in cancer, diabetes, and mental health care, where co-design approaches have yielded digital tools that improved self-management, decision-making, and communication between patients and clinicians [24,32,33]. These examples highlight HCD’s unique ability to uncover latent needs, address barriers that are often overlooked, and generate solutions that are contextually relevant and sustainable. Using an HCD approach, we conducted a qualitative study to identify patient and clinician needs and expectations to inform the design and development of a digital opioid safety intervention (herein referred to as “digital opioid safety tool”) for AYA with IBD.

## Methods

### Study Design

The HCD research process typically involves defining the problem and empathizing with end user needs, followed by brainstorming and ideation, prototyping, and testing potential solutions [24]. In this study, we focused on early formative phases of HCD: defining and empathizing with end user needs through collaborative exploration of perspectives and discussion of experiences among all participants. To that end, we first conducted a semistructured individual interview with AYA patients with IBD and IBD clinicians (including gastroenterologists, surgeons, and nurses). The primary goal of the interviews was to elucidate experiences with managing pain, current practices related to using or prescribing opioid medications, and potential gaps in the care pathways for AYA patients with IBD that could be contributing to higher rates of opioid use in this patient population.

To further clarify end user needs and begin brainstorming solutions, we conducted a co-design workshop, a well-established application within the HCD approach [34]. Bringing together patient and clinician interview participants, our goal for the co-design workshop was to gain insight, based on participants’ discussions and reflections as a group, into the challenges of managing pain in IBD care and potential solutions to enhance safety in pain management. Together, the interview and workshop findings establish a foundation for future development of a digital opioid safety tool tailored for AYA with IBD. The study design and reporting adhered to the COREQ (Consolidated Criteria for Reporting Qualitative Research) guidelines (Checklist 1) [35].

Our multidisciplinary team brought together a variety of experiences and expertise to the process of data collection and analysis. For example, 3 research team members are clinicians who see young patients with IBD (LC, SBH, and MVR), 2 research team members are patients with IBD (AA and SNB), and another team member is the father of a child with a complex GI condition (ABLB). Additionally, 2 team members have extensive design training and expertise (AA and ABLB), 4 team members are trained in community health research (MY, LC, DD, and NSB), and 3 team members have expertise in health services and outcomes research (MY, MVR, and SNB). Given prior literature describing power imbalances between patients and clinicians and/or researchers, we ensured that our team was intentionally multidisciplinary (ie, including patient, clinician, and researcher viewpoints) so that we could consider and balance those perspectives throughout the study [36,37]. To this end, we took several steps, particularly during the co-design workshop, to mitigate potential power imbalances between patient and clinician participants. These steps included abandoning formal professional titles in favor of first names, using interactive techniques such as structured “round robin” turns to equalize participation, and holding the sessions in a neutral, easily accessible space rather than a clinical boardroom, office, or hospital space [36,37]. Throughout the study planning, data collection (interviews and workshop), and analytic phases, team members acknowledged how their clinical knowledge and lived experiences (as researchers, patients, and/or clinicians) may have influenced their interpretation of the findings and emerging themes in the data. To ensure reflexivity, team members engaged in regular team discussions and memo writing to document analytic decisions.

### Participants

Semistructured interviews and the co-design workshop were conducted with AYA patients with IBD and IBD clinicians at Ann & Robert H. Lurie Children’s Hospital of Chicago and the Digestive Health Center at Northwestern Memorial Hospital.

We used Malterud and colleagues’ [38] model of information power to help inform the total number of study participants, indicating a narrow study aim: participants with experiences in living with IBD or caring for others who live with IBD; a strong dialogue during interviews and the co-design workshop, guided by prior relevant knowledge; and an appropriate thematic analysis. For patients, we focused on their experiences with pain management, opioid use, and the transition to adult care. We used a convenience sampling approach. Eligibility criteria for patients included (1) patients aged 15‐29 years and (2) previously diagnosed with IBD with a clinic visit record. The IBD clinics at both Northwestern’s Digestive Health Center and Lurie Children’s Hospital provided our team with a list of AYA patients, with their email contact information, who received IBD care from that facility and met our eligibility criteria. Eligible patients who met inclusion were invited to participate by email. In the summer of 2023, we informed these patients about the study by email and invited them to participate in the study. We then sent a weekly reminder via email to all eligible patients who had not responded. Interested eligible patients received an information sheet outlining our study aims, procedures, potential risks and benefits, privacy protections, and the voluntary nature of their participation. We scheduled the interview at participants’ convenience between June and August 2023 either in-person or by video-based conferencing. At the beginning of each interview, we included time to answer any questions the participant may have had about the study and to obtain informed consent for participation in the study.

For clinicians, the objective was to explore their perspectives on, and practices for, monitoring pain, prescribing opioids, and managing pain in AYA patients with IBD. All IBD-focused clinicians (16 in total) at the Digestive Health Center and the Lurie Children’s Hospital, including gastroenterologists, surgeons, nurses, and nurse practitioners, were invited to participate by email invitation. As in the patient interviews, we scheduled clinician interviews according to their convenience between July and August 2023 either in-person or by video-based conferencing. At the beginning of each interview, we included time to answer any questions the clinician participant may have had about the study and obtained informed consent for participation in the study.

### Data Collection

#### Interviews

Two team members (AA and SNB), a design innovation master’s level graduate student and a researcher (PhD) in health services research with expertise in GI disorders, respectively, trained in qualitative methods and design research, facilitated the 45-minute semistructured interviews using a moderator discussion guide for patients (Multimedia Appendix 1) and for clinicians (Multimedia Appendix 2). Both patient and clinician discussion guides were developed based on a review of literature on pain management and opioid use in the patient population with IBD, HCD principles, and input from study team members trained in qualitative methods and design research. The discussion guide was semistructured to focus on key topics while allowing exploration of other topics raised by participants. The patient discussion guide focused on personal experiences with IBD-related pain, health monitoring and communication preferences, decision-making processes, and perceived value and expectations of a digital opioid safety tool. The clinician guide addressed practices for monitoring medications and pain symptoms, communication strategies, and anticipated usefulness of a digital opioid safety tool. All data were collected in English.

#### Co-Design Workshop

We hosted a 2-hour co-design workshop in which patients and clinicians discussed, in a group setting, opportunities for improving pain management and opioid safety in IBD care. The workshop was facilitated by the same team members who facilitated the interviews, with the presence of many others from the team. At the start of the workshop, the goal of the study was shared with participants (patients, clinicians, and facilitators present) again, and all participants introduced themselves, including their role in the study and relevant training or experiences. As in prior studies involving co-design, we sought to follow a participatory approach focused on harnessing creativity and promoting active collaboration between patients impacted by IBD and the clinicians who care for this patient population [28-30]. All workshop participants had previously participated in individual interviews, and this included all 7 patient participants as well as 8 clinician participants. Five remaining clinicians declined to participate in the workshop due to scheduling conflicts. A slide deck guided the workshop session (Multimedia Appendix 3). As a study team, we reviewed prior literature [39-42] and used the interview data previously collected to inform 2 workshop activities that the team collectively felt would (1) resonate with the interview participants and (2) uniquely build upon their perspectives shared in the interviews. These workshop activities were facilitated by study coauthors trained in design research (AA and SNB) and guided by a third coauthor (ABLB) with extensive design expertise and experience (Textbox 1).

Textbox 1. **Workshop activities.**

- In activity 1, “Breaking the Rules,” participants joined small groups of 3‐4, reflected on themes from the earlier interviews, and identified underlying or unwritten “rules” that, in their view, influence pain management and opioid safety in inflammatory bowel disease care. We then asked participants to challenge those rules by asking questions such as “What aspects of this rule can we break?” or “Which rules would we like to keep?” Finally, we encouraged participants to generate potential new ideas or new rules for a hypothetical “new world.”

- In activity 2, “Wild Card,” we provided participants with 2 prompts to stimulate discussion, reflection, and brainstorming in small groups of 3‐4 participants about novel strategies to address the challenges of pain and high opioid use rates among adolescents and young adults with inflammatory bowel disease. The first prompt laid out a hypothetical scenario for participants: “After identifying an infiltrate across factories globally, all over-the-counter pain relievers have been recalled.” To foster discussion, we then asked participants to consider questions, such as “How can we control patient pain through alternate routes? How will patients be advised if they won’t have access to these medications?” The second prompt reflected another hypothetical scenario: “Earlier today, the Supreme Court ruled that it is now illegal for providers to talk about their patients with one another.” We then asked participants to consider their role as a health care provider or imagine themselves as one and describe how they would continue to care for their patients and address issues such as pain, particularly in cases of concurrent chronic conditions.

### Data Analysis

All interviews and the workshop session were audio recorded and transcribed verbatim. The interviews and workshop sessions were analyzed concurrently using thematic analysis [43]. Two research team members (SNB and LC) independently coded the transcripts using an inductive coding approach and the constant comparison method to compare data from an initial group of transcripts and generate a preliminary codebook. The 2 coders then collaboratively compared and refined codes, code applications, and code definitions to produce a final codebook (Multimedia Appendix 4), which was then applied to all transcripts. Discrepancies in code applications were reconciled through consensus [43,44]. Coded transcripts were analyzed within and across cases to develop themes, all of which represented needs and ideal attributes for the design of a digital opioid safety tool in this clinical setting. While interview and workshop data shared some overlap, they were distinct in that the interview data focused more on challenges experienced or needs expressed by participants. By contrast, the co-design workshop generated unique insights, building on the interviews, by inviting participants to consider innovative solutions related to opioid safety while engaging them in the workshop activities together. For both the interviews and workshop session, we again reflected on Malterud and colleagues’ [38] model of information power as a guide during our analysis meetings. In this context, development of our final themes and their categories continued until agreement was reached. MAXQDA (version 2022; VERBI Software GmbH) was used for data storage, coding, and thematic analysis.

### Ethical Considerations

The study was approved by the institutional review board at Northwestern University and all participants provided verbal consent (institutional review board number STU00216695). All study data were deidentified prior to analysis to protect participant confidentiality. Data were stored on encrypted, password-protected systems, and any Protected Health Information used for recruitment was securely maintained in a separate Health Insurance Portability and Accountability Act–compliant location with access limited to the study team. For their participation, all participants received a US $50 virtual gift card following each activity.

## Results

### Overview

Of 20 patients approached, 7 (35%) participants were recruited. The remaining patients (n=13) reported that they could not participate due to other competing responsibilities, time constraints, or scheduling conflicts related to summer travel. After 1 weekly recruitment reminder, 4 of the 20 eligible patients had replied that they were willing to participate, which resulted in 4 interviews. After 2 additional weekly reminders via email, 3 additional patients expressed their interest in participating, resulting in a total of 7 interviews. Of the 16 clinicians approached, 13 (81%) were recruited; the remaining clinicians cited lack of time or scheduling conflicts as the reason why they could not participate or did not respond to our invitation. Therefore, overall, 7 patients and 13 clinicians participated in the study. Table 1 summarizes participant characteristics. Clinicians included 8 physicians (including gastroenterologists and surgeons), 3 nurse practitioners, 1 nurse, and 1 psychologist. All clinicians focused on IBD clinical care.

**Table 1.** Demographic characteristics of the participants.

Characteristics	Participants
Patients (n=7)	
Age (years), mean (SD)	22 (2.8)
Sex, frequency (%)	
Female	6 (85.7)
Male	1 (14.3)
IBD^a^ diagnosis, frequency (%)	
Crohn’s disease	3 (42.9)
Ulcerative colitis	4 (57.1)
Age (years) at IBD diagnosis, mean (SD)	14.3 (3.5)
Had prior surgery to treat IBD, frequency (% yes)	3 (42.9)
Current IBD medications, frequency (%)	
Biologic therapy	5 (71.4)
Immunomodulator	1 (14.3)
5-ASA^b^	1 (14.3)
Clinicians (n=13)	
Sex, frequency (%)	
Male	6 (46.2)
Female	7 (53.4)
Profession, frequency (%)	
Physician	8 (61.2)
Nurse	1 (7.7)
Nurse practitioner	3 (23.1)
Psychologist	1 (7.7)

a IBD: inflammatory bowel disease.

b 5-ASA: 5-aminosalicylic acid.

**Figure 1.**
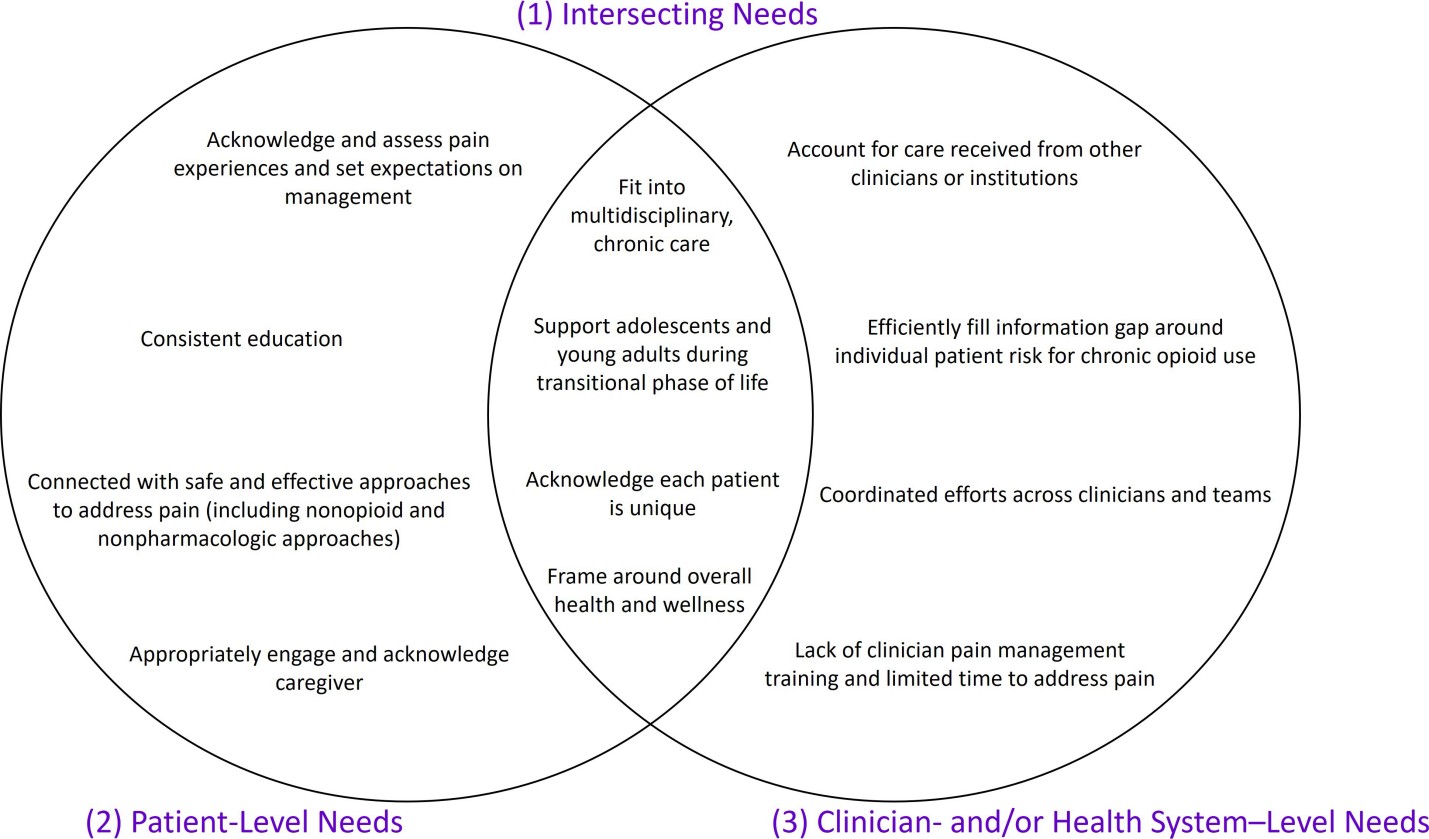
Ideal attributes and needs for a digital opioid safety tool for adolescents and young adults with inflammatory bowel disease. Emerging themes by relevance to the patient- and clinician- and/or health system–level needs (category 1: Intersecting Needs), patient-level needs (category 2), and clinician- and/or health system–level needs (category 3).

We organized the resultant themes into three broad categories: (1) Intersecting Needs (ie, relevant at the patient and clinician and/or health system level), (2) Patient-Level Needs (ie, relevant at the patient level only), and (3) Clinician- and/or Health System–Level Needs (ie, relevant at the clinician and/or health system levels only) to inform the design and development of a digital opioid safety tool. These categories helped to organize the insights shared by AYA patients and their clinicians, including needs for future interventions based on their experiences and roles. Our rationale for organizing themes in this way was to distinguish the unique needs of each group while still bringing their viewpoints together, particularly because the safety tool is intended to be used by both clinicians and patients. Therefore, our themes delineate these viewpoints by stakeholder group, although they may include viewpoints of participants representing a different group. Figure 1 demonstrates these themes by category. Themes are denoted in italicized text throughout this section.

### Category 1: Intersecting Needs

We derived key themes, collectively categorized as “Intersecting Needs,” based on data reported by clinicians and patients. These themes are outlined in Table 2 alongside representative quotes. A primary need emphasized by both groups is the *integration of safety interventions across multidisciplinary, chronic care settings*. Clinicians emphasized that effective management of AYA patients with IBD requires comprehensive coordination across clinicians from multiple specialties beyond gastroenterology (eg, psychiatry or primary care), necessitating interventions that streamline care and access to a patient’s history. Patients frequently reported the burden of consolidating records from various clinicians to ensure comprehensive information exchange during appointments. For example, 1 AYA patient described getting procedures done with 1 clinician yet discussing results and future management with a different clinician, complicating continuity of care and putting the onus of coordination across clinical specialties on the patient (Table 2). Clinicians and patients both highlighted that, while this may be relatively typical of the siloed infrastructure of health care in the United States, in a setting specific to AYA with IBD or other chronic conditions, it is important to consider that many of these young patients also face the burden of additional chronic comorbidities, such as mental health illnesses. Seamlessly coordinating care and coordination of management is necessary for a positive, safe, and healthy experience for the patients, while also minimizing gaps in care management. This extended to clinician and patient perceptions surrounding the design of a digital opioid safety tool, which they believed must address co-occurring comorbidities and the reality of which AYA patients with IBD need to seek care from multiple specialties beyond gastroenterology.

**Table 2.** Category 1: Intersecting needs for a digital opioid safety tool for adolescents and young adults with inflammatory bowel disease, stratified by theme.

Theme	Representative quote
Seamlessly fit into multidisciplinary, chronic care setting	“It was part of my initial talks with him to see what was going on, because my colonoscopy wasn’t with him. It was at a different doctor, so he was getting the results and talking about it with me.” [Patient]
Support adolescents and young adults during transitional phase of life	“My parents are pretty involved, but they live in Chicago. So for my infusions, I was getting those in Chicago, so I was actually flying back every few weeks to get those. And they’ve been very supportive. I use MyChart. I’m always messaging my doctor or the nurses, and I think those are the two biggest supporters in my healthcare journey.” [Patient] “Yeah, I think starting out, like calling insurance, that was all them. And then it just got easier for me to call because they kept being like, ’Oh, your daughter’s over a certain age, we need her on the phone.’ So it would just be easier for me to just call them. But still, if insurance really frustrates me, they offer to get on the phone with them, and sometimes they will. There’s an issue now of billing and stuff, so I think they’re probably going to get on the call with insurance for me. But in general, in terms of making appointments, I make that on my own with their consultation, because if I take the appointment in-person rather than on Zoom, they’ll take me to the doctor’s appointment. So I’ll go, ‘Hey, does this work?’ And then they know what’s going on, it’s in their calendar, they’re taking me there, kind of thing.” [Patient] “I feel like, with her going to college...at Lurie, what they do with kids when they’re seniors in high school, they are going to have a training session of how to handle things moving forward. Obviously, she’s going to go through that with the proper people at Lurie, and then she’ll probably be taking over the communications on the portal.” [Patient] “It can be hard though from an 18-year-old to assume all that responsibility and to attend appointments by themselves because they may not know the details of their disease history if they’ve been diagnosed for so many years, and their parents just scheduled all their appointments and all their infusions and so they truly don’t know.” [Co-design Workshop] “When patients are coming to the adult world and just feeling very alone and isolated we have wonderful support groups offered by…psychologists...So another way to connect IBD patients that are in their young teens with one another that are just navigating the adult world. But the CCF [Crohn’s & Colitis Foundation] also has resources, and there’s chapters throughout the nation that patients can reach out to.” [Co-design Workshop]
Acknowledge that every patient is different	“I guess someone, we do have patients that seek out opioids, and so those patients...And one, we can see their med list, and the patients who ask for it all the time definitely raises a flag, okay, is this someone that really has untreated, unmanaged pain or is it someone who’s more just opioid seeking?... every patient’s just so different that I don’t know if there’s one way to be able to best determine that.” [Nurse] “Different patients will have different kind of thresholds and ideas of how they define their own pain.” [Surgeon] “Sometimes I know my patients because I’ve been their nurse for five years, so I have a relationship with them and I may know this person has a high pain tolerance, they never complain about pain. So when they’re complaining about pain this is a red flag, versus someone that kind of always has pain. Doesn’t mean I don’t acknowledge it any less, but take that into consideration when they’re rating their pain.” [Nurse] “Everybody’s pain is so different and you can’t compare people’s pain because they all rate it in different ways.” [Patient]
Frame conversation around overall health and wellness	“...we’ve never heard anything about stats on addiction with kids with UC or any chronic disease, but what a scary place to be in for the family, that they’re trying to help their kids, and then one thing turns into another thing. One problem becomes a bigger problem. And also the correlation between physical health and having mental health issues because of having to face that on top of the disease, on top of addiction, that’s heavy.” [Patient] “That’s unfair because I’m 21. I want to experience what you people are experiencing.” And everyone else is like, “You can’t because you might get sick.” [Patient] “[With UC]...the correlation between physical health and having mental health issues because of having to face that on top of the disease, on top of addiction, that’s heavy.” [Patient]

Supporting AYA with IBD as they transition to independent care is another critical theme. The ages of 15‐29 years are marked by significant life transitions, including entering new academic or professional settings, relocating away from caregivers, and assuming primary responsibility for health management. Due to their positioning in terms of age and developmental stages, it is imperative to acknowledge the impact of a chronic disease and potential chronic opioid use for AYA with IBD and frame conversations and management plans, respectively. AYA patients expressed a desire for *support during this transitional phase of life*, such as continued support from caregivers during decision-making processes or even returning home for health care visits to maintain that support. Clinicians noted that many AYA may lack a full understanding of their diagnosis or treatment history, having only recently taken over care management responsibilities. Programs such as those at Ann & Robert H. Lurie Children’s Hospital of Chicago and the Crohn’s & Colitis Foundation were cited as existing resources designed to support AYA during this transitional phase through training sessions or support groups. However, both patients and clinicians emphasized the need for *individualized approaches***,** recognizing the heterogeneity of experiences of AYA. All patients, especially AYA whose circumstances vary and who may rely on caregivers more than adult populations, require unique approaches and care. Variations in pain thresholds, definitions of pain, and care priorities were also noted as key factors that influence each patient’s needs.

The chronic nature of IBD fosters ongoing relationships between clinicians and patients, allowing for a deeper understanding of individual care preferences. Patients stressed the importance of *framing conversations around overall health and wellness*, emphasizing the profound impact of physical pain on mental health and social functioning. Additionally, AYA patients expressed fear about long-term medication use, specifically anxiety about potential side effects and dependence. Therefore, addressing both the physical and psychological dimensions of IBD management requires open communication about pain management and medication use, supporting AYA patients as they navigate their evolving health care needs while navigating their overall development and life stages.

### Category 2: Patient-Level Needs

Table 3 highlights key themes reflecting patient concerns, needs, and experiences related to pain management in IBD care. Clinicians must actively *acknowledge and assess AYA patients’ pain experiences and set clear expectations for pain management*. One clinician noted that external factors such as sleep patterns and nutrition can significantly impact pain experiences, underscoring the need for a comprehensive understanding of each patient’s unique lifestyle and circumstances.

**Table 3.** Category 2: patient-level needs for a digital opioid safety tool for adolescents and young adults with inflammatory bowel disease, stratified by theme.

Theme	Representative quote
Acknowledge/assess pain experiences and set expectations on pain management	“Managing certain things in your personal life, so understanding how sleep affects your pain, or understanding how things like changing your nutrition can alleviate some of the more mild symptoms. And then I think the last thing we also had, which it was distraction. So I think [*patient*] had talked about how being distracted and having things to think about other than the pain that she was in actually did help alleviate the symptoms a little bit because there was just something else to think about. Mental health factors.” [Surgeon]
Connect patients with safe and effective approaches to address pain (including nonopioid and nonpharmacologic approaches)	“I wish there was something that I could take immediately or...A hundred percent. Because these medicines are long-term, and so it’s just getting my inflammation levels down over time. But I definitely wish it was like a, I take a pill or something and it would make me feel better instantly.” [Patient] “So I was really irritated at that point because I’m like, ‘All right, here I’m trying my best to manage my pain, but no one’s really helping me. They’re just trying to like, here’s some Tylenol,’ the stuff I take at home. The stuff that literally does absolutely nothing for me. So it was probably the worst thing that I went through at that time.” [Patient] “I really haven’t asked if there’s anything I could do in the meantime. I’ve been told yoga, and that’s why I’ve tried to change my eating and fitness habits. I have done yoga more often to try and help that. So just easy, self ways I can do it rather than reaching out for help. Yeah, I just haven’t reached out. I just always assumed it was because taking this medication is the only thing I can be doing. I didn’t know if there was other options.” [Patient] “because of the fact that I got it young, so did you, the fact that we were diagnosed so young and they tried to treat so quickly, I’ve read that you can become reliant your whole life if you start on such heavy medications at a young age. And so, if you have to go on such a heavy medication that it’s addictive and you become reliant on it, yeah, that’s terrible.” [Patient] “There’s a mindset that surgery is kind of the worst thing. Surgery represents failure. And I feel like patients come, and when they’re talking to me about surgery they take it as a personal failure, should they have done something different? And I feel like surgery should be considered a toolbox, not the worst case scenario.” [Surgeon]
Consistently educate patients	“A lot of parents too will try and go for avoiding biologics and some of the other prescription medications just give side effects and what they read online. So more education on what those options are can hopefully help address the base issue of where they are coming from.” [Surgeon] “Especially with my medicine, getting my medicine so late, I’m worried that this won’t work and what will happen for the future....I’m still learning how all this works, but it’s not a medicine that I take for a short amount of time. It’s a medicine I’m on indefinitely. So I’m worried what things come up in the future. This isn’t like a, ‘It’s fixed.’ It’s just helping the problem. So I do worry about in the future what could possibly happen.” [Patient]
Appropriately engage and acknowledge caregiver views	“And even the change between medications this year, I’d say my parents were very involved in wanting to make sure I’m making the right decision and everything.” [Patient] “Oh. well, I tell you [mom] everything about my symptoms and stuff and if I’m concerned about something, and we discuss if it’s something we think we can treat or if we need to take it to the doctors, and so we’ll usually message them together. We’ll be talking as we write it to them to make sure we hit all the right points.” (Mom): I’ll send it, but if it’s something complicated, then I’ll be like, ‘[patient], here, read this. Am I describing your symptoms the right way?’ And she’ll say yes or no or, ‘Change this,’ and then we send it to them.” [Patient] “And I think if I were to be prescribed some kind of opioid in the future...I don’t know. I think my parents would feel very strongly about that. I think that’s when they would actually really step in and try and find something else before I would take that.” [Patient] “And then it just got easier for me to call because they kept being like, ‘Oh, your daughter’s over a certain age, we need her on the phone.’ So it would just be easier for me to just call them. But still, if insurance really frustrates me, they offer to get on the phone with them, and sometimes they will...But in general, in terms of making appointments, I make that on my own with their consultation, because if I take the appointment in-person rather than on Zoom, they’ll take me to the doctor’s appointment.” [Patient] “A lot of parents too will try and go for avoiding biologics and some of the other prescription medications.” [Surgeon]

As emphasized in category 1, addressing information gaps regarding individual patient risk for chronic opioid use requires clinicians to fully understand the patient’s specific concerns, questions, and available resources. Both patients and clinicians emphasized the importance of *connecting patients with safe and effective pain management strategies, including nonopioid and nonpharmacologic options*. Several AYA patients reported limited awareness of pain management options beyond medication. For instance, 1 patient recalled being advised to try yoga and dietary adjustments, assuming that those were the only alternatives to long-term medication use. In contrast, other patients reported being offered medication as the sole pain management option. Clinicians advocated for a “toolbox” approach to care, integrating diverse strategies to manage pain effectively. During the co-design session, a clinician addressed the misconception that “surgery represents failure,” despite surgery being a viable treatment option, further emphasizing the need for balanced and comprehensive patient education. *Education must be consistent* and tailored to each patient’s needs, information level, and overall health goals. Clinicians also noted that parents of AYA patients may avoid opioid prescriptions based on perceived risks, misinformation, or negative experiences. Therefore, more education on the empirical understanding of nonopioid pain management alternatives and how to address the root issue of the patient, or their child’s pain, can help relieve patients and their caregivers’ concerns and strengthen their understanding.

Caregivers often hold valuable insights regarding medication history, treatment side effects, and patient preferences, which can be vital for effective pain management. Many AYA patients receive their diagnoses and begin their journey with IBD care from a young age, during which their caregivers are the primary decision makers and documentarians of their care. As AYA transition into adulthood, they assume responsibilities for themselves. AYA patients expressed a desire for continued caregiver input, particularly during decision-making about complex treatment options. Likewise, clinicians emphasized that caregiver perspectives should be actively considered to support well-rounded, patient-centered care. Ensuring that caregivers remain appropriately informed and engaged can strengthen the patient-caregiver-clinician triad, promoting more effective, cohesive care throughout the AYA care journey.

Enhancing caregiver education on evidence-based nonopioid pain management options can alleviate concerns and promote more informed decision-making. Effectively *engaging and acknowledging caregivers in the care of AYA patients* with IBD is crucial, particularly as patients transition from pediatric to adult care. Many AYA patients receive their diagnosis in childhood, with caregivers initially serving as primary decision makers and record-keepers. As patients assume responsibility for their own care—an overwhelming transition for anyone but especially for AYA with chronic diseases (ie, IBD)—they often encounter challenges navigating complex systems. For example, 1 patient shared that their parents typically handled insurance-related calls for them, but once they turned 18 years of age, insurance providers required that the patient be on the phone. This shift required a strong understanding of the processes which the patient may not have been involved in before turning 18 years of age. This transition underscores the importance of caregiver involvement to ensure continuity of care, to prevent critical information gaps, and to support AYA patients in managing new responsibilities, alongside their diagnoses, with confidence.

### Category 3: Clinician- and/or Health System–Level Needs

Multiple clinician- and health system–level themes emerged from discussions with AYA IBD-focused clinicians, as outlined in Table 4. Clinicians emphasized the importance of *accounting for care provided by other clinicians or institutions*. For example, 1 nurse described using Epic (an electronic medical records software) to track patients’ pharmacy records and medication history, regardless of the prescribing clinician or institution. Comprehensive awareness of all care received is essential to ensure appropriate and coordinated treatment for AYA with IBD. A significant need identified by clinicians is *addressing patient information gaps regarding individual risk for chronic opioid use*. A nurse practitioner noted that patient education, including pain management, medications, and disease understanding, is a substantial component of their role. However, each patient presents with unique circumstances, including varying disease manifestations, social determinants of health, and support systems. Thus, clinicians emphasized the importance of tailored education and resources that address each patient’s specific risk profile within the context of their IBD and care. Additionally, clinicians highlighted the necessity of *coordinated opioid safety efforts across care teams*. Without such coordination, the risk of overprescribing, inadequate education, or inconsistent messaging may increase, leading to potential overdosing, suboptimal outcomes, or adverse health experiences. On a systemic level, clinicians reported *lack of training in pain management specific to AYA with IBD*, despite having high levels of expertise in other areas. They also noted that time constraints due to systemic factors and patient volume often limit their ability to thoroughly address pain management, further underscoring the need for streamlined, effective communication and educational strategies.

**Table 4.** Category 3: clinician- and/or health system–level needs that should be addressed in a digital opioid safety tool for adolescents and young adults with inflammatory bowel disease, stratified by theme.

Theme	Representative quote
Account for care received from other clinicians or institutions	“But there is, in Epic you can see, look at the patients’ pharmacies and see what medications a patient has picked up. So that is a cool tool you can use (across different medical systems that the patient goes to).” [Nurse practitioner]
Efficiently fill information gap around individual patient risk for chronic opioid use	“I feel like it’s just constant patient education, whether it’s about pain or medication administration or just disease education. That’s a huge, huge part of our job just because there are so many questions and so many unknowns and you encounter something for the first time.” [Nurse practitioner]
Opioid safety must be a coordinated effort across clinicians and teams	“They’re highly trained and have strong expertise in one area or in just closely related areas. But for the patient, their experiences, all of those things are sometimes interacting.” [Co-design workshop]
Acknowledge the lack of clinician pain management training and limited time to address pain	“a provider has this very high level of expertise, the medical provider, but doesn’t always empower the patient to take information with them to their other providers. So maybe they have the knowledge but don’t know to communicate it or how to communicate it in a way that the patient can use it if they’re supposed to be their care coordinators.” [Co-design workshop]

## Discussion

### Principal Findings

In this study, we used HCD principles [24] and qualitative research methods [45] to examine and identify end user perspectives to inform the future design of a digital opioid safety tool. We engaged AYA patients with IBD and IBD clinicians to understand their perspectives and needs as potential end users of such a tool. Our findings highlight, first, the importance of supporting collaborative relationships among patients, caregivers, and clinicians to promote safe opioid use; second, the need to contextualize and frame opioid- and pain-related discussions according to AYA patients’ age and developmental stage; and finally, the value of applying HCD methods in clarifying directions for digital safety tools tailored to the lived experiences of AYA with IBD, a population at high risk of opioid overdose and other serious harm. Taken together, these findings provide a foundation for the development and implementation of future efforts that aim to actively engage end users (ie, patients and clinicians) together in optimizing opioid safety for AYA with IBD, seamlessly fit into both patient care experiences and clinician workflows, and address their unique needs and perspectives. Based on the findings of this study, our team identified salient design recommendations for consideration as a digital opioid safety tool is developed for this population, presented in Table 5.

**Table 5.** Practical design recommendations for a digital opioid safety tool for adolescents and young adults with inflammatory bowel disease based on emerging qualitative themes.

Design recommendation	Relevance	Need addressed	Rationale
1. Integrate structured inter- and previsit prompts and elicitation tools to routinely capture patient-reported pain experiences, psychosocial contributors, and care priorities to inform clinician-patient discussions.	Patient and clinician levels (Intersecting Needs)	Clinicians reported limited time to comprehensively assess pain experiences and contextual factors influencing pain, while patients expressed a desire for their lived experiences to be recognized in care decisions.	Clinicians and patients both underscored the importance of individualized, holistic understanding of pain. This finding suggests that digital tools should support systematic elicitation of patient context prior to clinical encounters. Previsit questionnaires or reflection prompts can efficiently identify patient priorities and prepare clinicians to support more personalized and collaborative pain management conversations.
2. Establish a personalized educational content library that delivers opioid and pain management education tailored to AYA^a^ patients’ knowledge gaps, developmental stage, and preferred learning modality (eg, text, audio, and video).	Patient level	AYA patients reported inconsistent and incomplete education about opioid risks and nonopioid pain management options, while clinicians identified persistent patient knowledge gaps and variability in individual risk profiles.	Clinician and patient needs intersected around the need for consistent, accessible education on opioid risks and pain management. Clinicians emphasized the importance of addressing individual patient knowledge gaps, aligning with patients’ expressed desire for clear, reliable information. This finding suggests that digital safety tools should assess individual educational needs and deliver tailored content in formats that align with patients’ expectations, as illustrated by providing a multimedia educational library with patient-directed content selection.
3. Embed optional caregiver-facing educational and decision support modules with patient-controlled permission settings to support shared understanding during transitions to independent care.	Patient level	AYA patients rely on caregivers for historical knowledge and decision-making support, yet transitions to adult care often create information gaps and role ambiguity between patients and caregivers.	Patients emphasized the value of continued caregiver involvement in complex decisions, while clinicians highlighted caregivers’ contributions to medication history and treatment context. These intersecting needs indicate that opioid safety tools should support flexible caregiver engagement. Allowing patients to selectively share educational content and decision summaries with caregivers can promote continuity of care while respecting patient autonomy.
4. Provide clinician-facing conversation guides and prompts that support shared decision-making and reframe opioid and pain discussions around overall wellness rather than isolated symptoms.	Clinician level	Narrow, medication-focused conversations may exacerbate fear, stigma, or misunderstanding among AYA patients and undermine trust and engagement.	Patients emphasized that framing discussions around overall health and wellness improved trust and alignment. This suggests that opioid safety tools should include clinician-facing supports that guide developmentally appropriate, value-based conversations, reinforcing partnership and shared decision-making rather than transactional prescribing discussions.
5. Enable cross-institutional visibility of opioid prescriptions, pain management plans, and relevant comorbidities through integration with existing electronic health systems and medication histories.	Clinician and/or health system level	Fragmented care across specialties and institutions places coordination burdens on patients and increases the risk of inconsistent messaging or unsafe prescribing.	Clinicians and patients both identified the need for coordinated, multidisciplinary care, particularly given the prevalence of comorbid mental health conditions in AYA with IBD^b^. This finding implies that opioid safety tools must support integration of information across care contexts, enabling clinicians to access a comprehensive view of patient care and reduce gaps in opioid safety management.
6. Design opioid safety features to align with existing clinical workflows and best practice guidelines, including concise summaries, automated risk flags, and just-in-time educational prompts to minimize clinician burden.	Clinician and/or health system level	Clinicians reported insufficient time and training to address pain management and opioid safety amid competing clinical demands.	Clinicians emphasized that tools must be feasible within real-world practice to be adopted. Embedding streamlined opioid safety supports within existing workflows can enhance usability, reduce cognitive burden, and promote sustained use, aligning safety interventions with routine clinical care.

a AYA: adolescents and young adults.

b IBD: inflammatory bowel disease.

By centering the experiences and needs of both patients and clinicians, our findings contribute uniquely to the evidence-base at the intersection of IBD care, pain management, and opioid use. Pain management and opioid use among AYA with IBD are well-documented challenges [45-48]. AYA with IBD face distinct challenges due to their transitional life stage and evolving health care needs [49,50], increasing their risk for short-term or chronic opioid use [51]. In our study, clinician and patient needs intersected (category 1), highlighting a gap in consistent, accessible communication and education tailored to individual patient needs. Clinicians emphasized the importance of addressing patient knowledge gaps regarding opioid risks, aligning with patients’ expressed need for consistent, accessible education. For future prototyping and design, this suggests a need for methods capable of assessing individual patients’ educational needs and providing education tailored according to those needs. As highlighted in Table 5, recommendation 2, a digital opioid safety tool could include a library of educational materials with options for text, audio, and video, and a patient-friendly method of identifying the content needed and format desired.

Additionally, patients emphasized the value of caregiver engagement as part of their care team, while clinicians highlighted the necessity of coordinated, multidisciplinary approaches. Collaborative efforts among patients, caregivers, and clinicians are therefore essential for safe and effective opioid management and risk assessment among AYA patient populations. This finding underscores the need for digital opioid safety tool developers to focus on interoperability across care contexts to facilitate seamless information exchange (Table 5, recommendations 3 and 5). This raises potential design tensions, such as how informatics systems can mediate access to and disclosure of information in a nuanced manner (eg, between AYA and parental caregivers). Having the ability to access a comprehensive view of patient care through a digital tool can strengthen clinical care teams, reduce gaps in opioid safety management, and allow AYA to engage their caregivers as necessary.

Both patients and clinicians emphasized the importance of holistic care that acknowledges the distinctive challenges of AYA living with IBD. AYA often balance school, work, extracurriculars, and social transitions alongside new responsibilities for independently managing their chronic, fluctuating health, which can complicate engagement in care [49]. Furthermore, each patient’s individual circumstances, evolving disease trajectories, and unique developmental needs demand tailored and shared decision-making delivered through education and collaboration [52]. Participants noted that by consistently framing health discussions around holistic wellness rather than isolated symptoms, clinicians can provide more comprehensive, developmentally attuned care for AYA with IBD and help patients feel supported in managing the physical and psychosocial dimensions of their IBD. Future design work should consider how to best support clinicians and patients in identifying the unique patient circumstances that are relevant. Future digital tools could support previsit elicitation workflows that capture the patient’s broader life context and updates between appointments, which can allow clinicians to adequately monitor patient needs and address pertinent experiences (Table 5, recommendation 4).

Our study demonstrates that HCD can extend to the critical domain of opioid safety in IBD through careful and consistent engagement of clinicians, patients, and others involved in the IBD care continuum. Bringing these groups, and their naturally distinct but complementary perspectives, together begins to fill an important gap where few patient-centered digital tools currently exist. The interviews and co-design workshop that we conducted serve as a helpful first step in this direction to unpack patient and clinicians’ underlying perceptions influencing pain management practices. Additional design work will be useful to build on this and consider, for example, how to foster a sense of partnership between these groups to sustain longer-term opioid safety. As our research advances to design the process, formats, and tools needed to integrate a digital opioid safety tool into the care of AYA with other painful chronic digestive disorders, it will also be important to collaborate and consider the needs, from a design perspective, of other groups involved in care delivery for AYA with chronic digestive disorders, including clinicians beyond gastroenterology and GI surgery, electronic health record architects, and health system leaders to ensure interoperability and feasibility.

This study lays out an adaptable model for future research that seeks to characterize the unique needs and perspectives among AYA with IBD and their clinicians to develop targeted health care interventions in this care setting. As shown in many clinical areas beyond IBD [53-55], HCD presents an especially promising opportunity in addressing opioid safety in IBD because it moves beyond traditional patient education to cocreate solutions that reflect the lived realities of patients, caregivers, and clinicians. As we build on these findings to develop and integrate a digital opioid safety tool into the care of AYA with IBD, future research is needed to understand the extent to which HCD can help drive meaningful and sustainable reductions in opioid use in this vulnerable patient population.

### Limitations

This study has several limitations. Participants were recruited exclusively from Northwestern Memorial Hospital and Ann & Robert H. Lurie Children’s Hospital in Chicago, which may limit the generalizability of findings to other settings. The sample was predominantly female, and the recruitment population was largely White, potentially overlooking the broader racial and socioeconomic diversity present in the IBD community, although the findings provide important groundwork to guide more inclusive future research. Additionally, while existing studies address opioid use across broader GI populations, this study focused narrowly on a relatively small sample with IBD [15,45,46], potentially limiting applicability to other GI conditions. These limitations should be considered in the context of existing methodological literature establishing that the goal of qualitative research is, often, to investigate discrete in-depth phenomena (vs generalizing to larger populations) [56]. Smaller sample sizes, such as those in this study, can be justified with the appropriate information power generated through data analysis [38,57]. Therefore, the study provides a strong foundation, despite these limitations, for future work aimed at optimizing digital opioid safety interventions for AYA with IBD based on end user experiences and needs.

Future design processes and research must also account for socioeconomic and structural barriers that influence opioid risk and digital engagement among marginalized AYA populations [58]. Digital health interventions can unintentionally exacerbate health disparities if structural barriers such as digital literacy, device or internet access, and socioeconomic constraints are not considered during development [59,60]. User-centered and co-design approaches attempt to mitigate these risks by involving patients, caregivers, and clinicians throughout the design process to ensure that technologies reflect the needs of diverse users with consideration of potential constraints. Similarly, prior research on co-designed digital health interventions for AYA suggests that engaging end users during development can improve usability, accessibility, and relevance across varying socioeconomic contexts [61].

### Conclusions

In this study, we used an HCD approach to qualitatively assess the needs and expectations of AYA patients with IBD and IBD clinicians regarding opioid safety. By directly engaging stakeholders, we identified overlapping priorities that extend beyond pain control to encompass education, communication, and coordinated care across the AYA care continuum. Our findings highlight critical insights for the foundation of using HCD to inform the design of sustainable interventions for AYA with IBD: first, the importance of building collaborative relationships to support safe opioid use; second, the need to frame pain discussions within a holistic, developmentally attuned approach to AYA wellness; and third, the potential of HCD to generate practical, scalable digital tools tailored to the lived experiences of this high-risk group.

Moving forward, our findings indicate that for digital health interventions to be effective in chronic disease management, they must move beyond passive symptom monitoring to facilitate active clinical coordination and shared decision-making. By integrating end user perspectives, our results lay the groundwork for co-designed digital tools that can improve opioid safety, strengthen multidisciplinary care, and ultimately enhance quality of life for AYA with IBD.

## Supplementary material

10.2196/92202 Multimedia Appendix 1 Patient discussion guide for moderators.

10.2196/92202 Multimedia Appendix 2 Clinician discussion guide for moderators.

10.2196/92202 Multimedia Appendix 3 Co-design workshop presentation slides.

10.2196/92202 Multimedia Appendix 4 Codebook.

10.2196/92202 Checklist 1 COREQ 32-item checklist.

## Abbreviations

AYA: adolescents and young adults
COREQ: Consolidated Criteria for Reporting Qualitative Research
GI: gastrointestinal
HCD: human-centered design
IBD: inflammatory bowel disease
