# Supplementary material for: End User Needs and Perspectives for a Digital Opioid Safety Tool in Adolescents and Young Adults With Inflammatory Bowel Disease: A Qualitative Human-Centered Design Study
Source: JMIR Form Res. 2026 Jul 31;10:e92202. doi: 10.2196/92202 (PMC13426124; doi:10.2196/92202)
Supplement: Multimedia Appendix 1 [file formative-v10-e92202-s001.docx]

**Multimedia Appendix 1: Patient Discussion Guide for Moderators**

Getting to Know the Participant (10 min):

1. Tell me a little bit about yourself
   1. In school? Work? Married? Kids? Pets? Sports/hobbies?
2. Tell me about your IBD journey
   1. Esp. any surgeries/procedures that may have required pain management
3. How did your path bring you to NM/Lurie?
4. How did you get connected with the Chronic Opioid Assessment and Screening Tool (COAST) research team?

Pain Experience (20 min):

1. Tell me about about a time when you recently had to manage pain
   1. ould be from a flare or non-IBD problem
2. How did you manage it?
   1. Why did you choose this option/these options?
   2. Were you confident this was going to help your pain?
      1. Did it work?
3. Did you consult a doctor?
   1. Your gastro?

*If consulted a doctor:*

1. Tell me about what led up to you deciding to contact the doctor
2. How did you and your care team decide on the best course of action?
   1. Collaborative?
   2. Prescriptive?
3. Did you feel like the plan was appropriate (i.e. would address your pain)?
4. Did you follow that course of action?
   1. If not, why?
      1. What didn’t you do that you were supposed to?
      2. What additional measures did you take?
5. What did follow up look like?
6. Do you have other doctors besides this one?
   1. (if yes): Did you share this incident with them?
      1. Did you share any medication taken during the event with them?

*If didn’t consult a doctor:*

1. Why did you decide not to reach out to your care team about your pain?
2. How did you decide how to treat your pain?
3. What would prompt you to call the doctor in the future?
4. Did you worry about your pain recurring?
5. Would you follow this same approach in the future?
   1. Do you think it’s sustainable?
6. Tell me about the last time you tried something new for your pain (meds, exercises, alt. therapies, etc.)
   1. What prompted it?
   2. How did you decide what to try?
   3. Who did you consult?
   4. Tell me about how you coordinated this with your caregiver(s) and/or care team

Coordination of Care (10 min):

1. Let’s zoom out - what does your “care landscape” look like?
   1. Are all your doctors at NM/Lurie?
2. Adolescence and young adulthood is a time of transition
   1. Have you had to change doctors (including your gastro?)
   2. What role did your parent/caregiver play in this transition?
3. Tell me about your parent’s/caregiver’s involvement in managing your health
   1. How do the three “points of the triangle” coordinate?
      1. Did you consult your parent/caregiver during the pain management episode you told me about earlier?
      2. Does this change if you’re not physically in the same place as your caregiver (i.e. at college)?
4. Do you keep track of your medical records personally?
   1. How?
5. Tell me about the last time you had to share care records between your providers
   1. How did you do it?
   2. What was the process like?
6. Do you feel like your doctors take into account your other conditions and experiences with other clinicians?

Wrap-Up (5 min)

1. Is there anything else you would like to share that we haven’t talked about yet?
2. Do you have any questions before we wrap up?
