# Supplementary material for: End User Needs and Perspectives for a Digital Opioid Safety Tool in Adolescents and Young Adults With Inflammatory Bowel Disease: A Qualitative Human-Centered Design Study
Source: JMIR Form Res. 2026 Jul 31;10:e92202. doi: 10.2196/92202 (PMC13426124; doi:10.2196/92202)
Supplement: Multimedia Appendix 2 [file formative-v10-e92202-s002.docx]

**Multimedia Appendix 2: Clinician Discussion Guide for Moderators**

Getting to Know the Participant (5 min):

1. Tell me a little bit about yourself
   1. NM/Lurie? Role? Tenure?
2. Tell me about your experience with IBD patients
   1. AYA patients?

3. How did you get connected with Chronic Opioid Assessment and Screening Tool (COAST) research team?

Response (10-15 min):

1. If a patient comes to you with pain, what are the first steps you take?
   1. Questions you ask yourself?
   2. Questions you ask them?
   3. History you check?
2. Tell me about the last time a patient came to you with acute pain
   1. Could be from a flare or non-IBD problem
3. Why did you choose this option/these options?
   1. Were you confident this was going to help your patient’s pain?
      1. Did it work?
      2. Was there follow up?
4. How did the care team decide on the best course of action for the patient?
   1. Collaborative?
   2. Prescriptive?
5. Tell me about the last time a patient came to you with chronic pain
   1. How did the process look different?
   2. Were there different factors you considered?
      1. What were they?
6. In both instances, did you feel like the plan was appropriate (i.e. would address the patient’s pain)?
   1. Was anyone else on the team (incl. the patient) concerned?
   2. Any worries about the pain recurring?
7. Would you follow this same approach in the future?

Patient History (5-10 min):

1. How do you assess pain?
   1. Is there any relativity aspect to pain assessment?
2. How do a patient’s previous experience with opioids influence the way you approach their pain?
3. Tell me about the last time you had to share care records between your patients’ providers
   1. How did you do it?
   2. What was the process like?
   3. How did you feel about the information you did and didn’t have access to?
      1. Were you able to make an informed decision re: your patient?

Wrap-Up (5 min)

1. Is there anything else you would like to share that we haven’t talked about yet?
2. Do you have any questions before we wrap up?
