# Supplementary material for: End User Needs and Perspectives for a Digital Opioid Safety Tool in Adolescents and Young Adults With Inflammatory Bowel Disease: A Qualitative Human-Centered Design Study
Source: JMIR Form Res. 2026 Jul 31;10:e92202. doi: 10.2196/92202 (PMC13426124; doi:10.2196/92202)
Supplement: Multimedia Appendix 3 [file formative-v10-e92202-s003.pptx]

## Slide 1
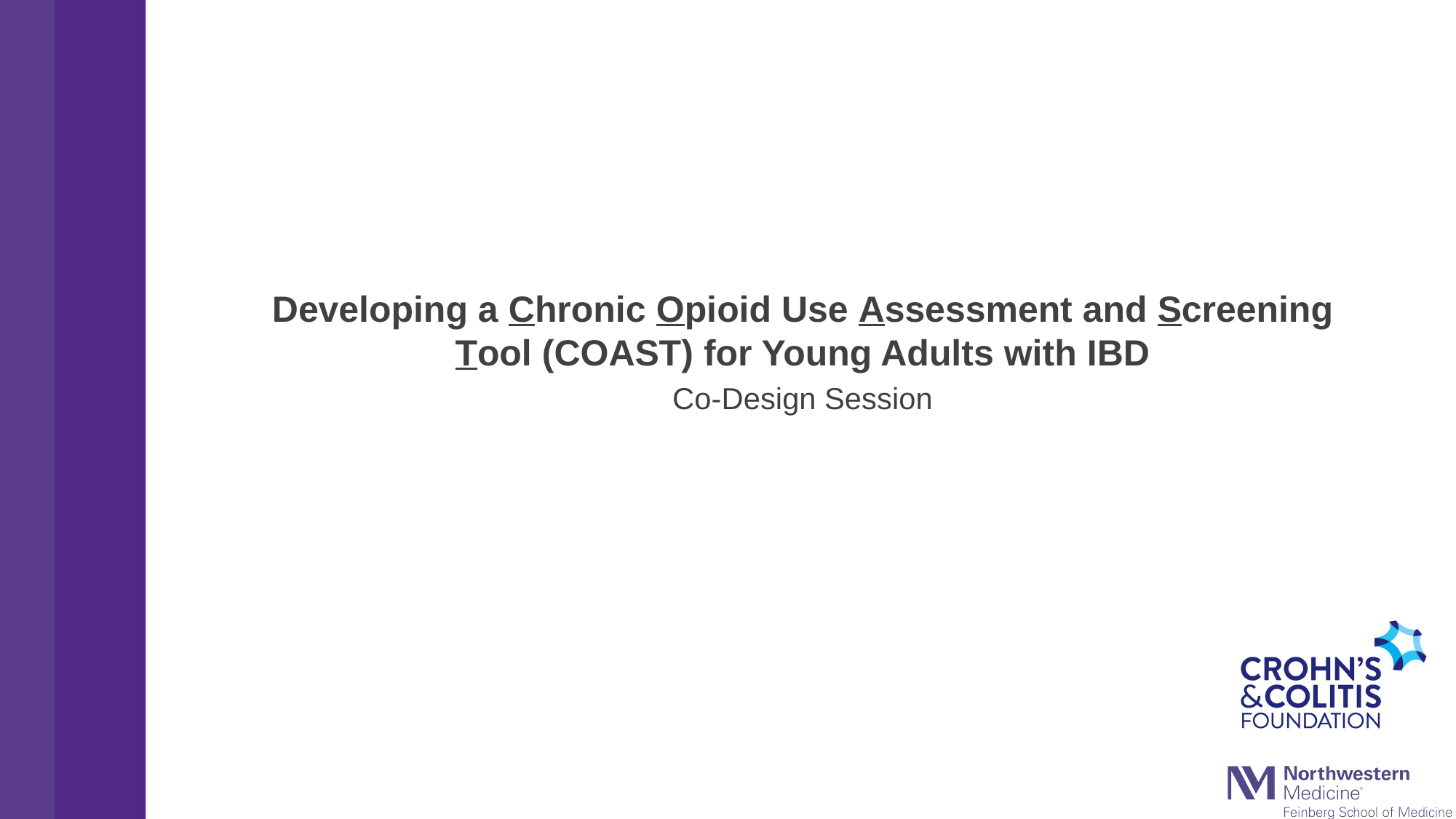

Developing a Chronic Opioid Use Assessment and Screening Tool (COAST) for Young Adults with IBD
Co-Design Session

## Slide 2
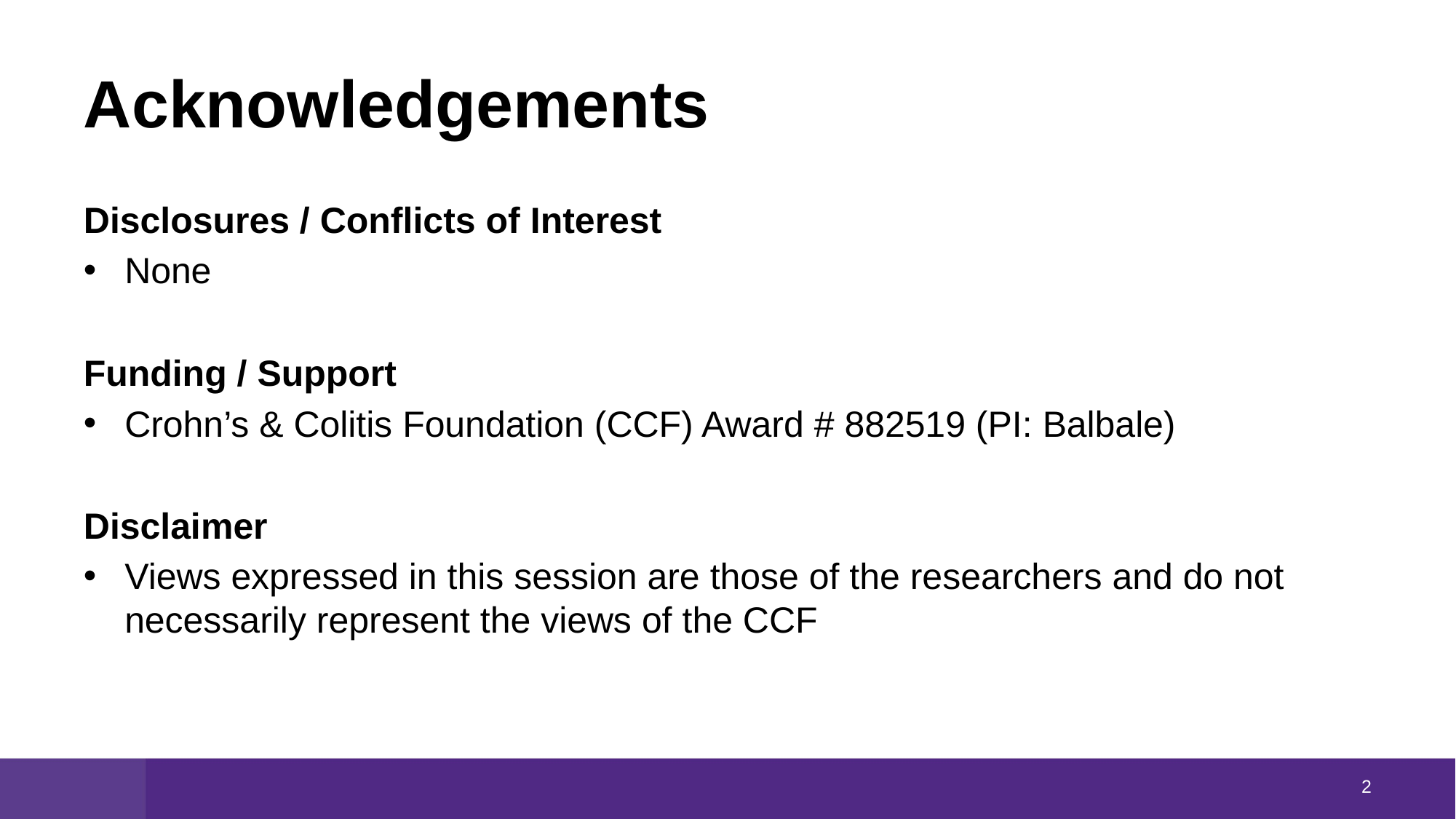

# Acknowledgements
Disclosures / Conflicts of Interest
None
Funding / Support
Crohn’s & Colitis Foundation (CCF) Award # 882519 (PI: Balbale)
Disclaimer
Views expressed in this session are those of the researchers and do not necessarily represent the views of the CCF
1

## Slide 3
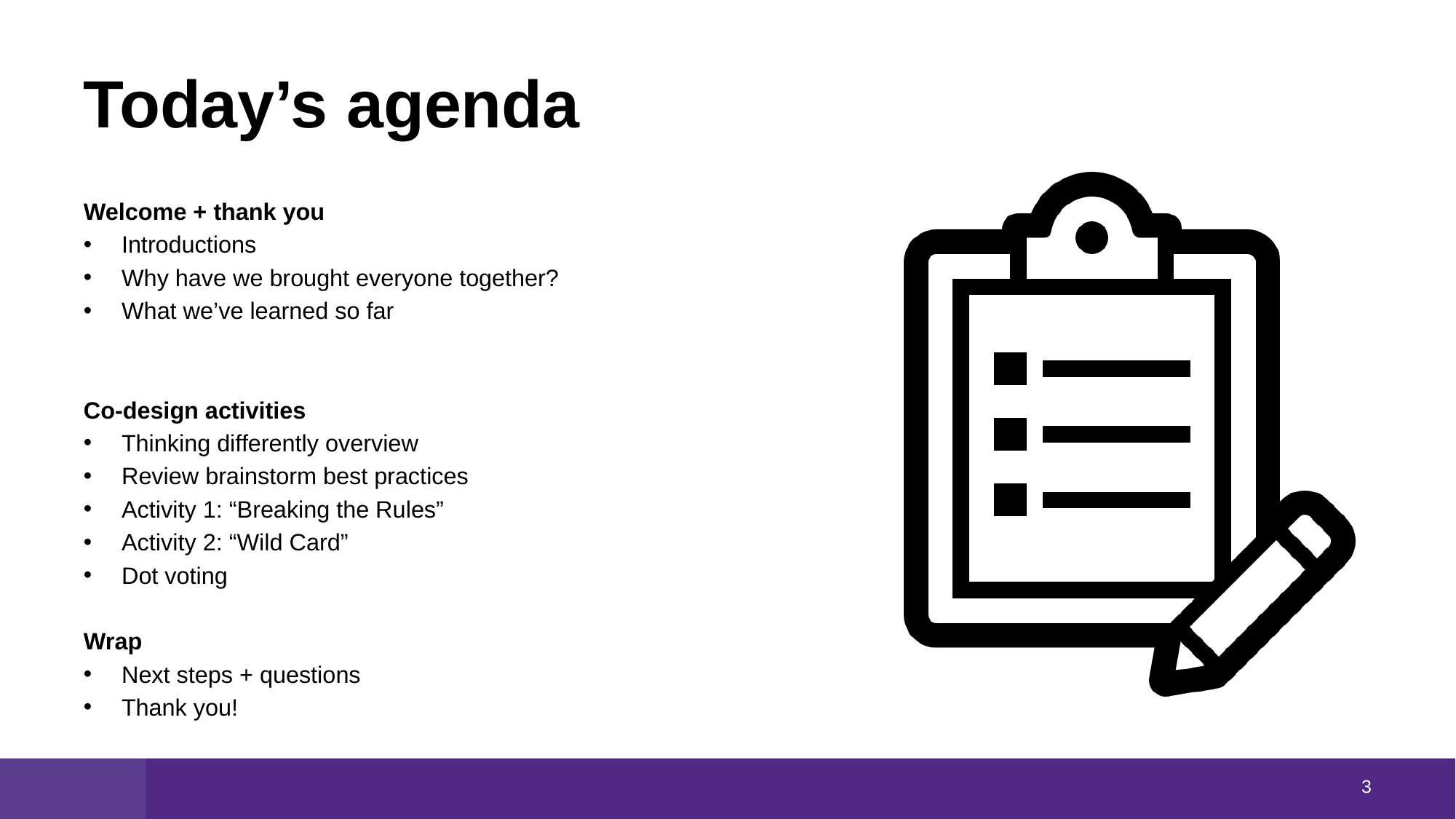

# Today’s agenda
Welcome + thank you
Introductions
Why have we brought everyone together?
What we’ve learned so far
Co-design activities
Thinking differently overview
Review brainstorm best practices
Activity 1: “Breaking the Rules”
Activity 2: “Wild Card”
Dot voting
Wrap
Next steps + questions
Thank you!
2

## Slide 4
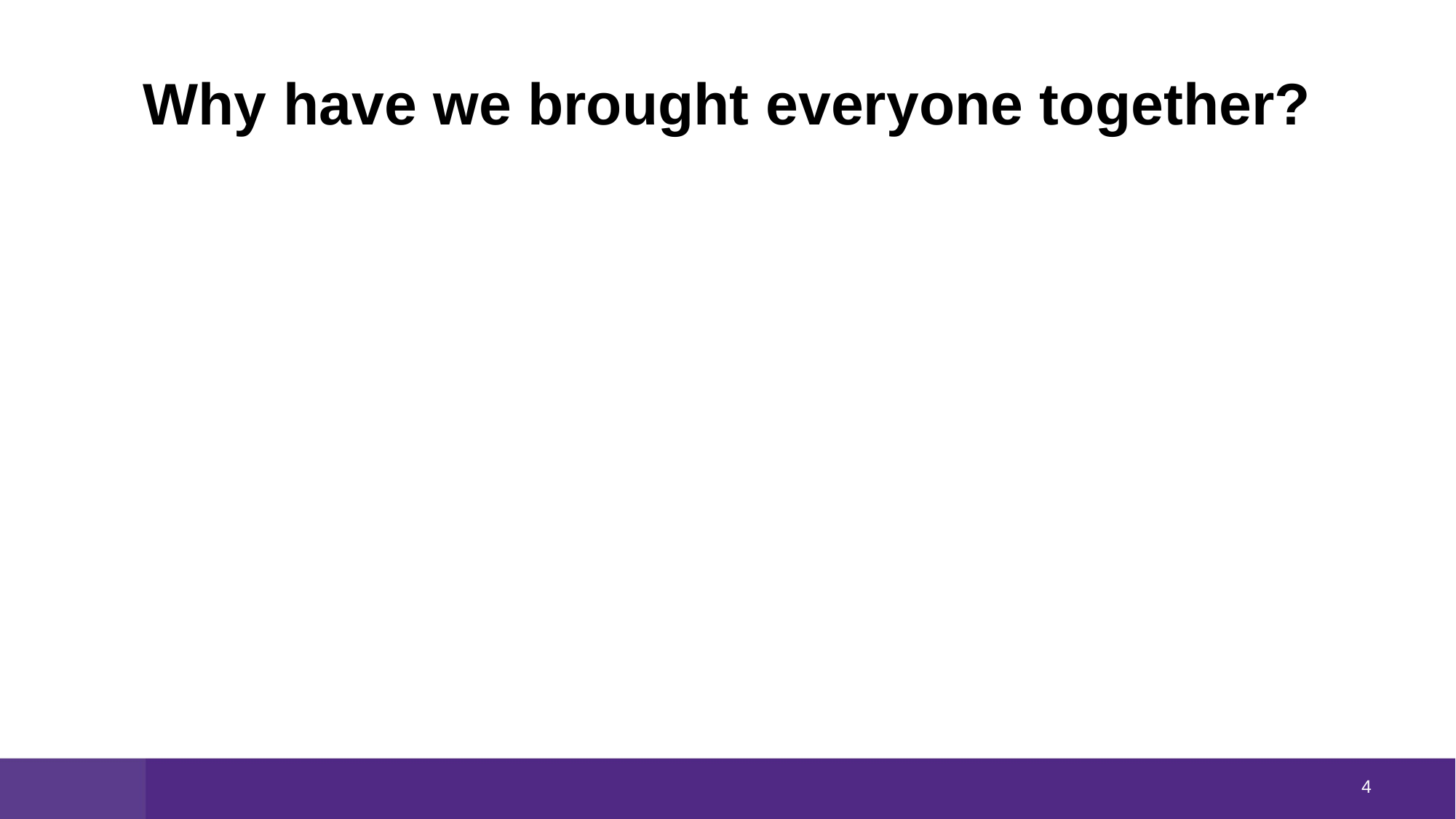

# Why have we brought everyone together?
3

## Slide 5
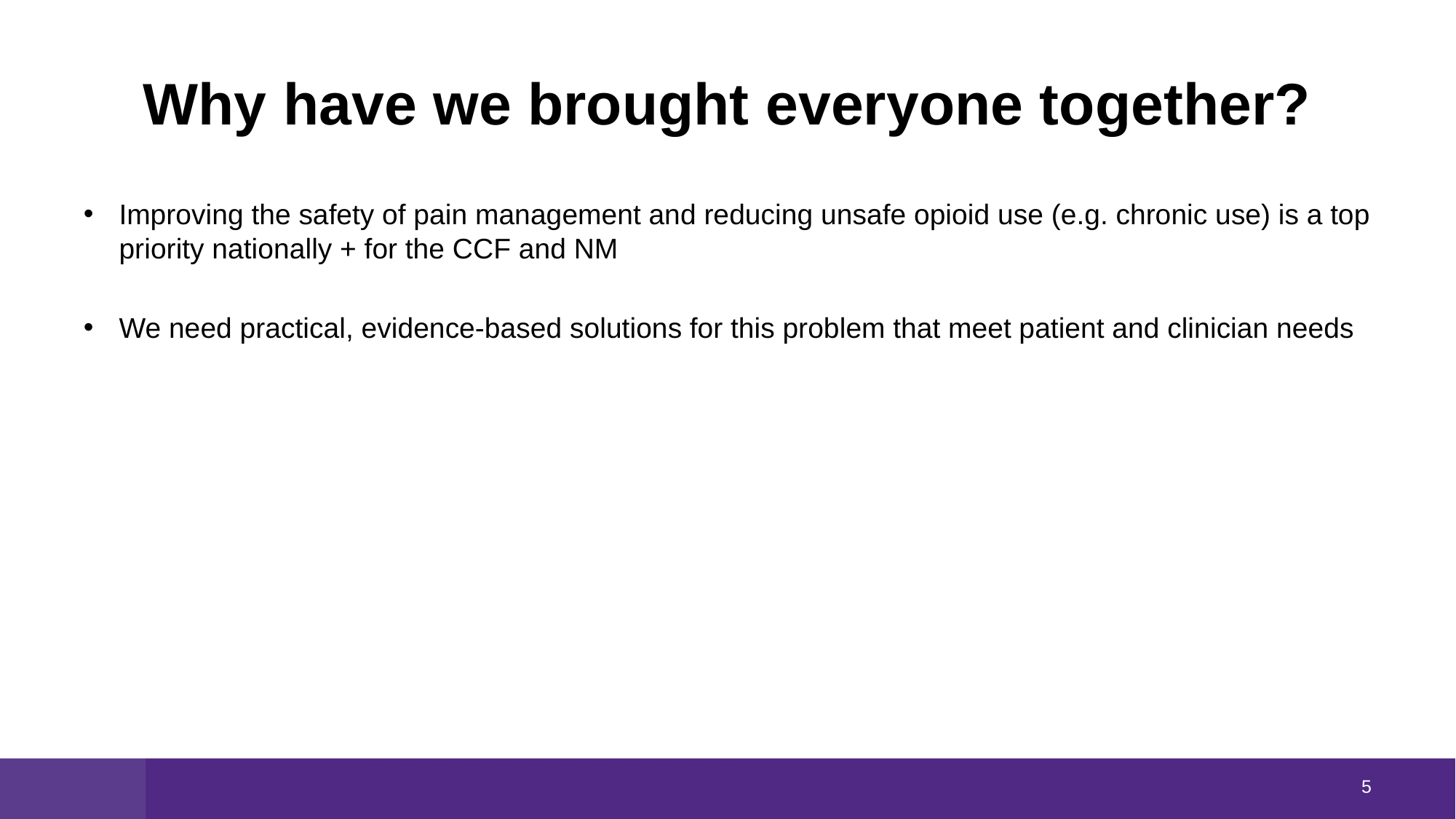

# Why have we brought everyone together?
Improving the safety of pain management and reducing unsafe opioid use (e.g. chronic use) is a top priority nationally + for the CCF and NM
We need practical, evidence-based solutions for this problem that meet patient and clinician needs
Adolescents and young adults (AYA) with IBD have complex healthcare needs in a vulnerable stage of life
When it comes to pain management / opioid use  patients and clinicians are not always on the same page
From our perspective, working directly with you to design a potential solution is key  our goal is to make it make sense!
4

## Slide 6
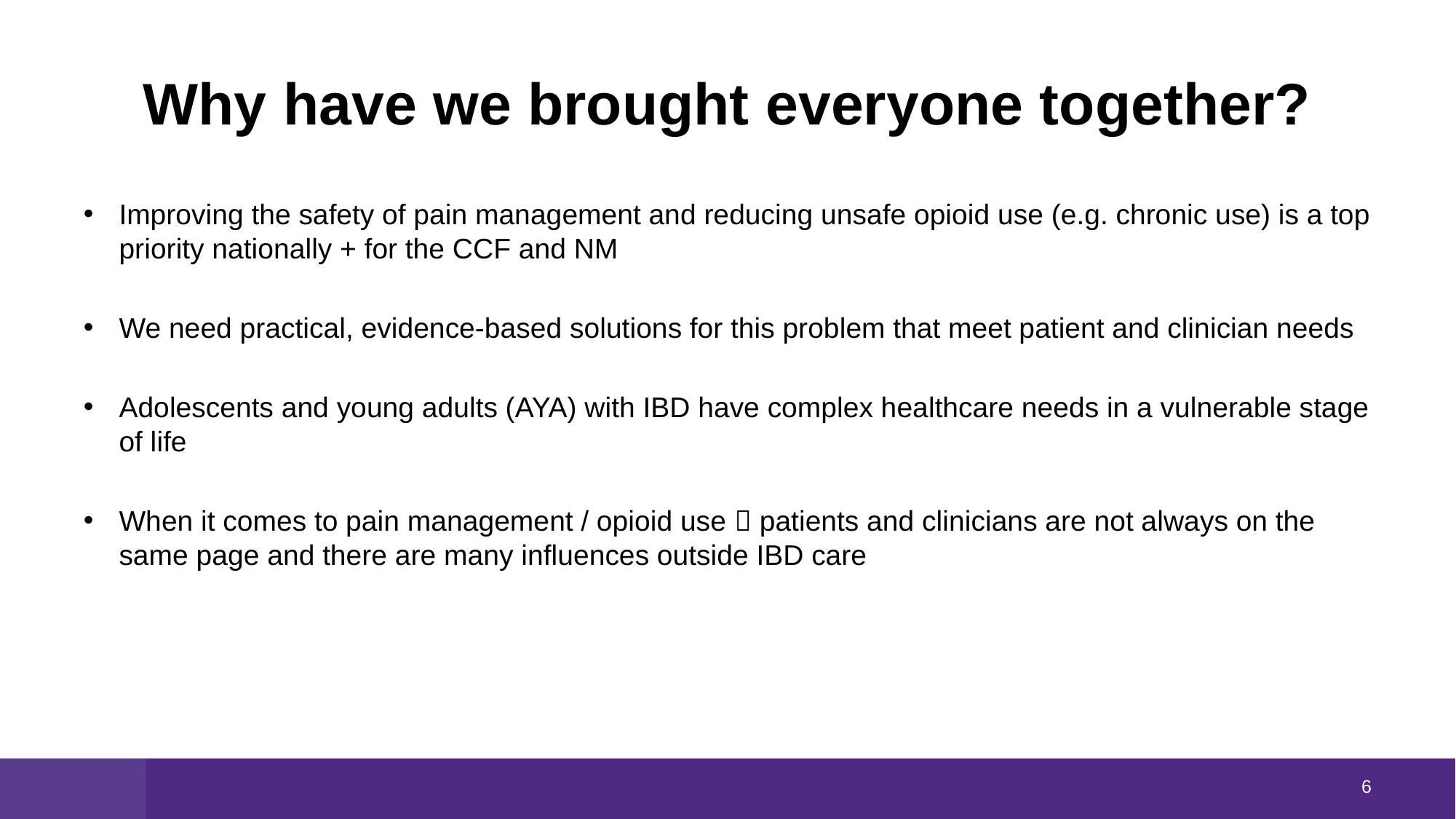

# Why have we brought everyone together?
Improving the safety of pain management and reducing unsafe opioid use (e.g. chronic use) is a top priority nationally + for the CCF and NM
We need practical, evidence-based solutions for this problem that meet patient and clinician needs
Adolescents and young adults (AYA) with IBD have complex healthcare needs in a vulnerable stage of life
When it comes to pain management / opioid use  patients and clinicians are not always on the same page and there are many influences outside IBD care
From our perspective, working directly with you to design a potential solution is key  our goal is to make it make sense!
5

## Slide 7
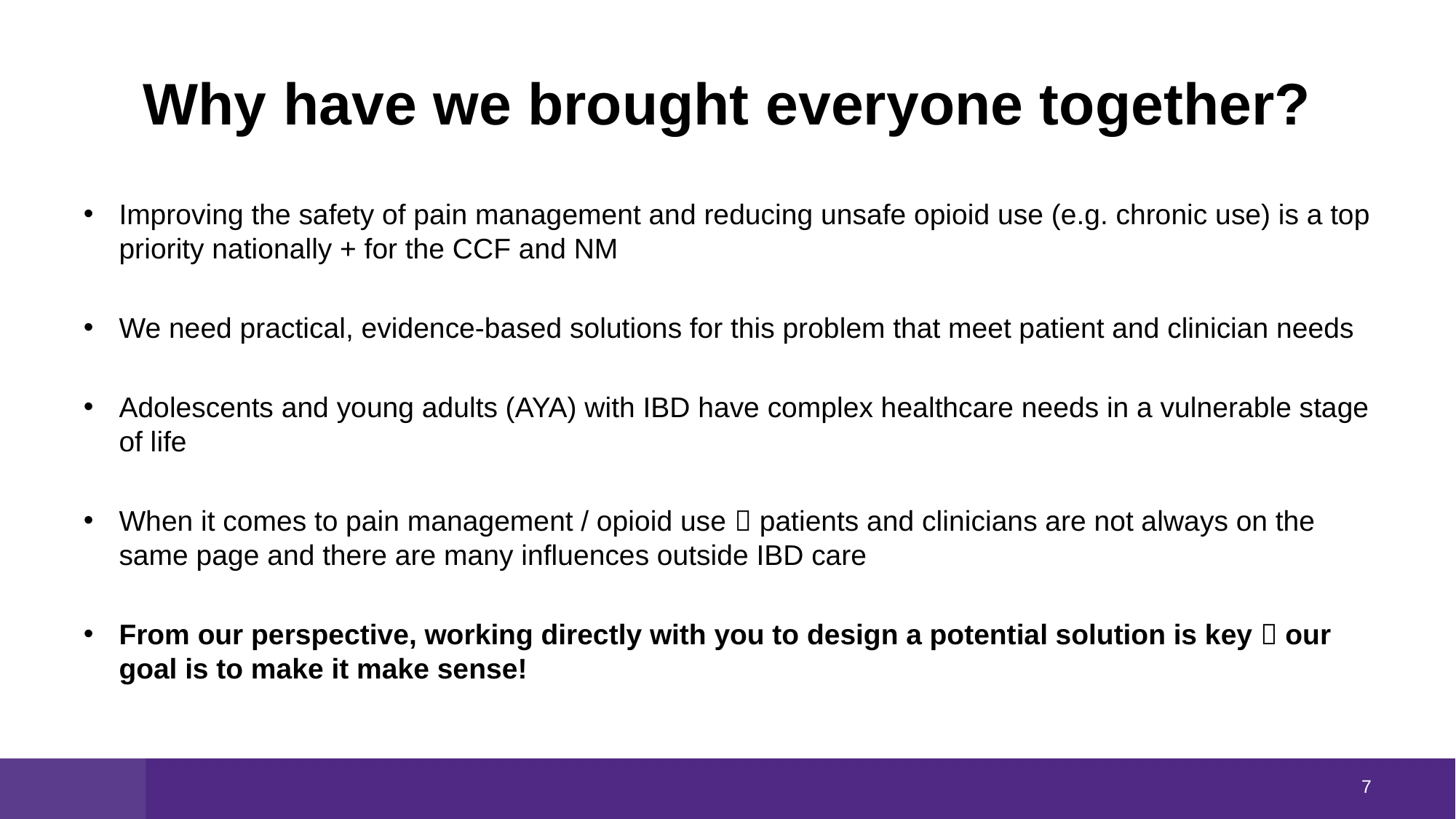

# Why have we brought everyone together?
Improving the safety of pain management and reducing unsafe opioid use (e.g. chronic use) is a top priority nationally + for the CCF and NM
We need practical, evidence-based solutions for this problem that meet patient and clinician needs
Adolescents and young adults (AYA) with IBD have complex healthcare needs in a vulnerable stage of life
When it comes to pain management / opioid use  patients and clinicians are not always on the same page and there are many influences outside IBD care
From our perspective, working directly with you to design a potential solution is key  our goal is to make it make sense!
6

## Slide 8
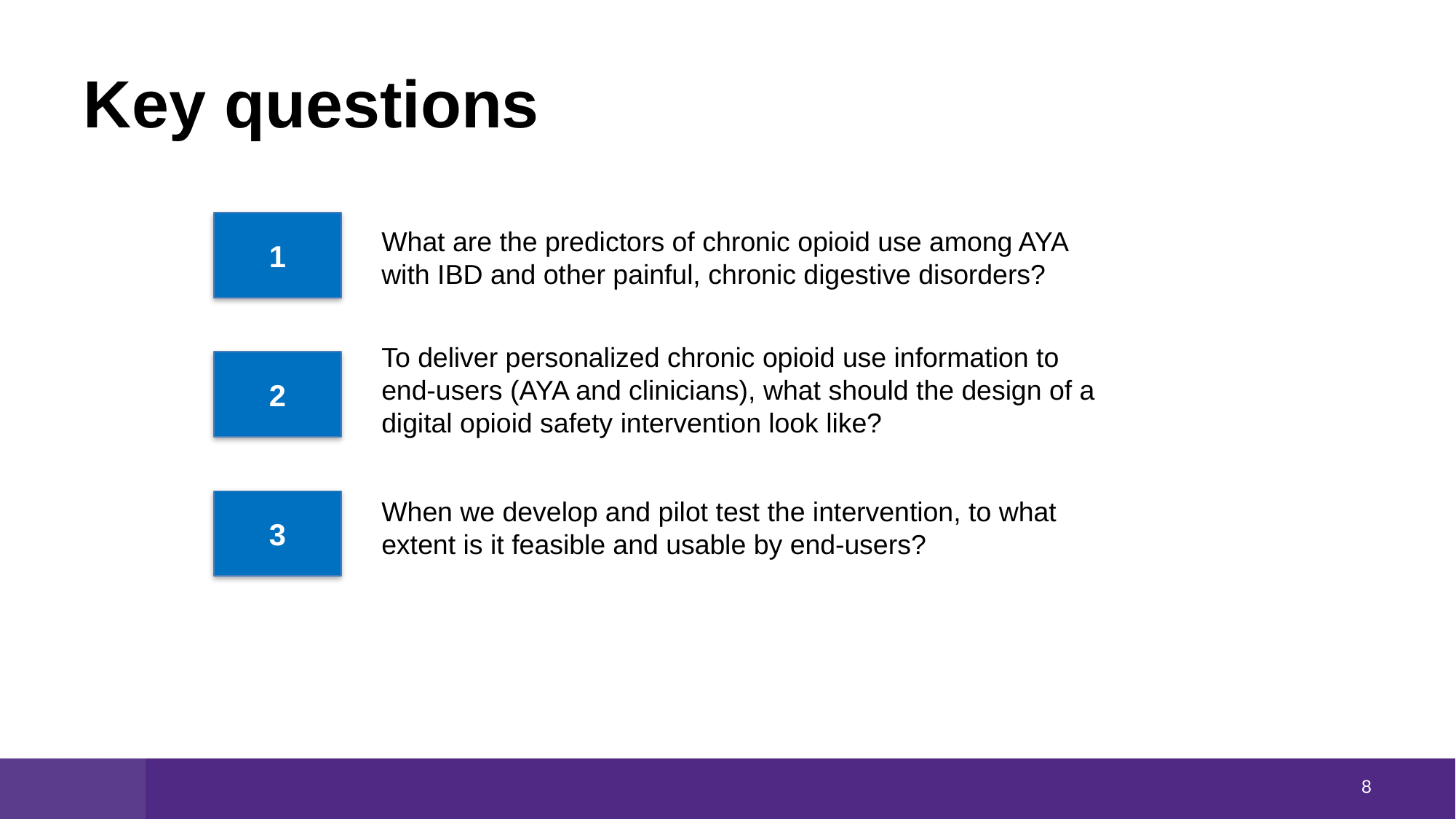

# Key questions
What are the predictors of chronic opioid use among AYA with IBD and other painful, chronic digestive disorders?
1
To deliver personalized chronic opioid use information to end-users (AYA and clinicians), what should the design of a digital opioid safety intervention look like?
2
When we develop and pilot test the intervention, to what extent is it feasible and usable by end-users?
3
7

## Slide 9
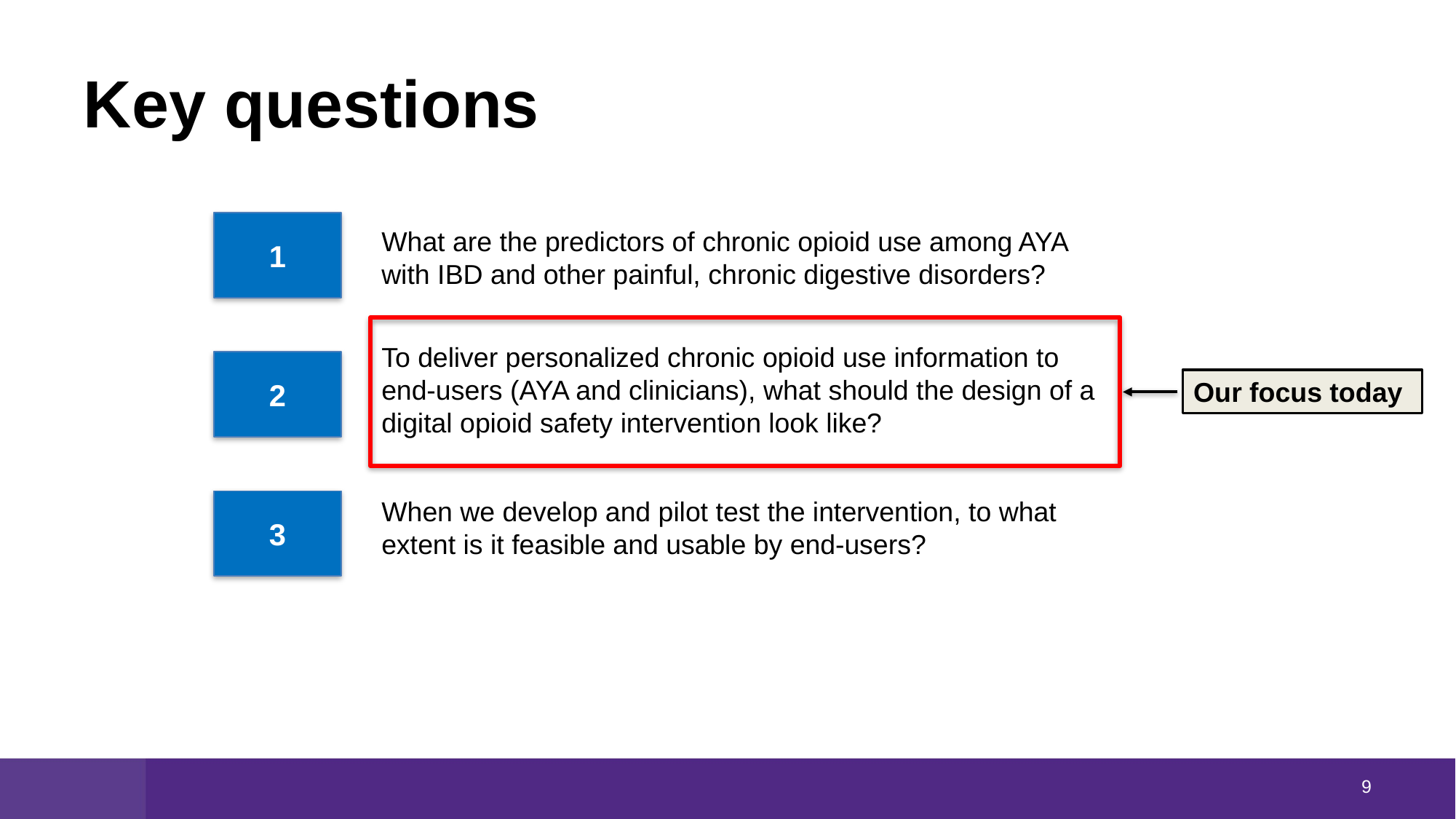

# Key questions
What are the predictors of chronic opioid use among AYA with IBD and other painful, chronic digestive disorders?
1
To deliver personalized chronic opioid use information to end-users (AYA and clinicians), what should the design of a digital opioid safety intervention look like?
2
Our focus today
When we develop and pilot test the intervention, to what extent is it feasible and usable by end-users?
3
8

## Slide 10
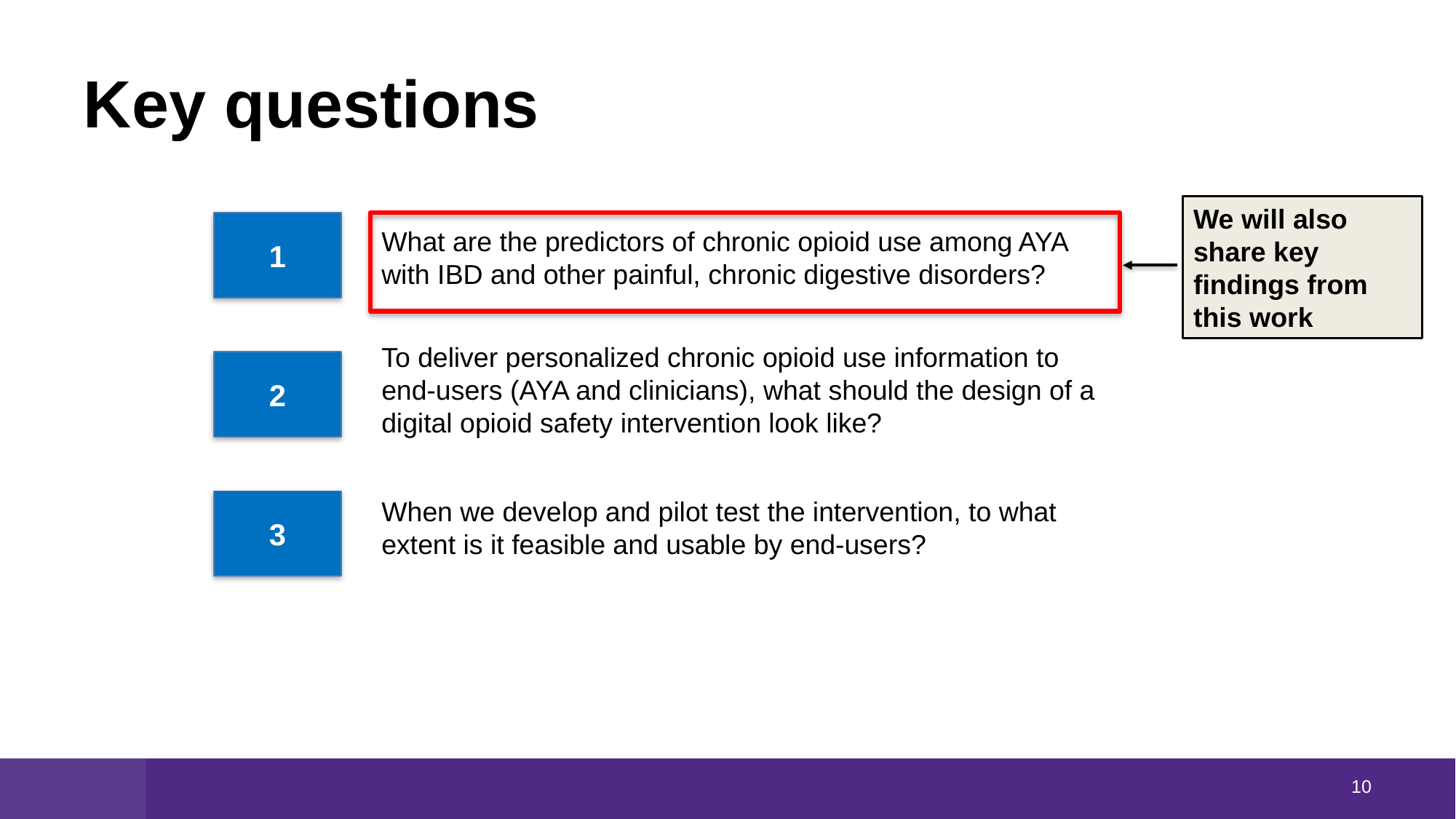

# Key questions
What are the predictors of chronic opioid use among AYA with IBD and other painful, chronic digestive disorders?
We will also share key findings from this work
1
To deliver personalized chronic opioid use information to end-users (AYA and clinicians), what should the design of a digital opioid safety intervention look like?
2
When we develop and pilot test the intervention, to what extent is it feasible and usable by end-users?
3
9

## Slide 11
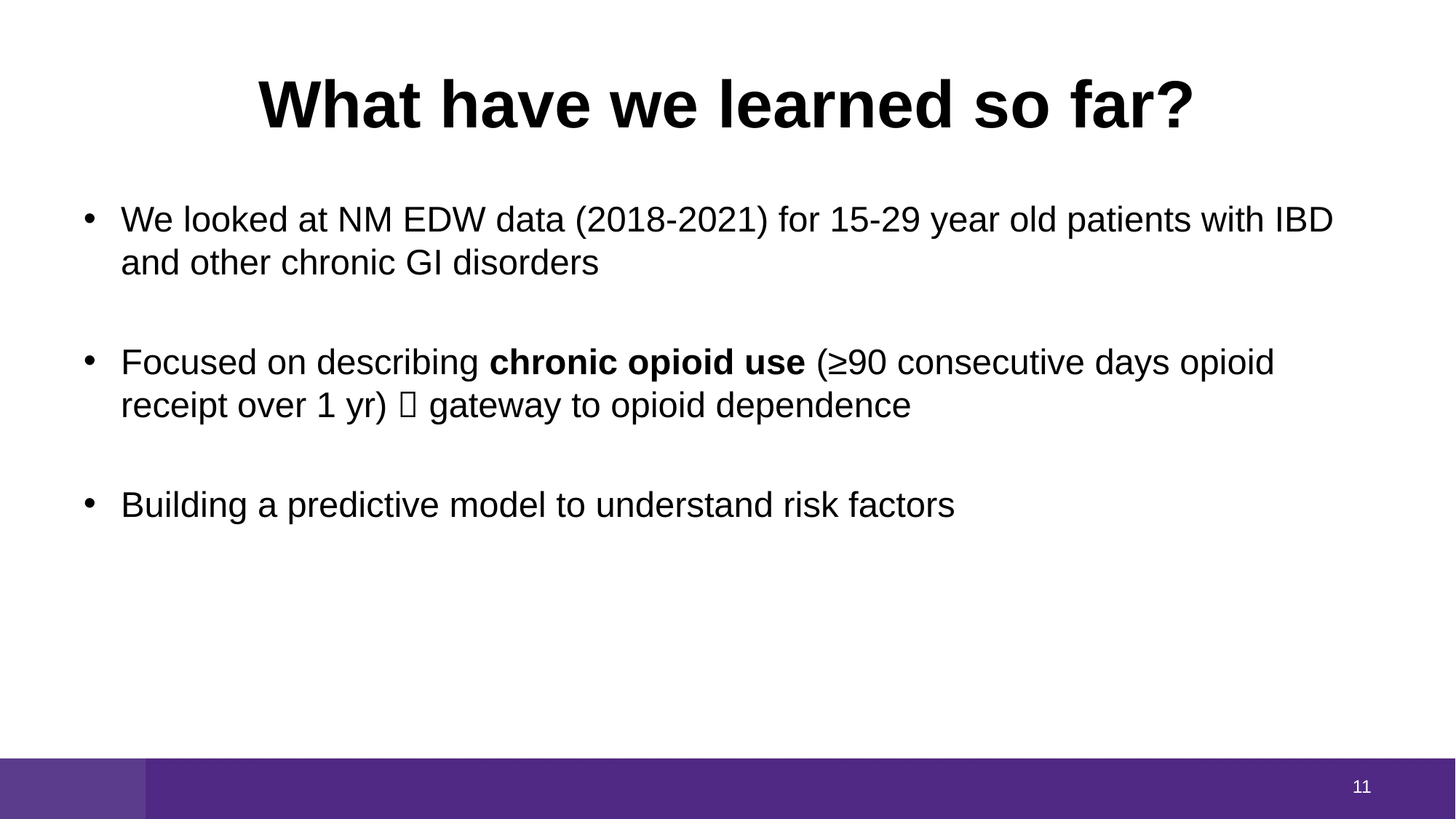

# What have we learned so far?
We looked at NM EDW data (2018-2021) for 15-29 year old patients with IBD and other chronic GI disorders
Focused on describing chronic opioid use (≥90 consecutive days opioid receipt over 1 yr)  gateway to opioid dependence
Building a predictive model to understand risk factors
In a cohort of ~9K patients, >30% had ≥1 opioid prescription
Among those (~3K patients), chronic opioid use ranged ~50-67%
10

## Slide 12
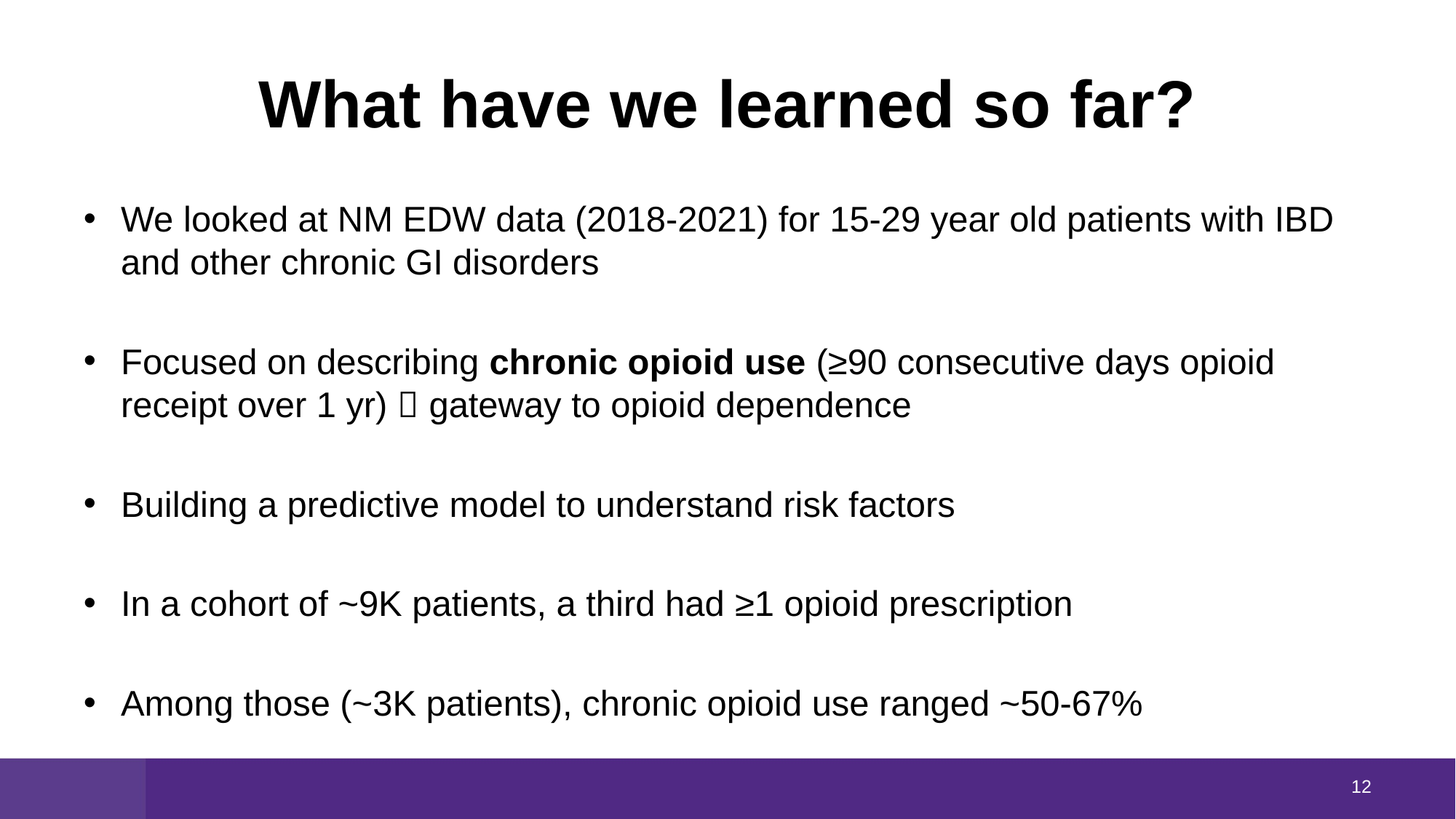

# What have we learned so far?
We looked at NM EDW data (2018-2021) for 15-29 year old patients with IBD and other chronic GI disorders
Focused on describing chronic opioid use (≥90 consecutive days opioid receipt over 1 yr)  gateway to opioid dependence
Building a predictive model to understand risk factors
In a cohort of ~9K patients, a third had ≥1 opioid prescription
Among those (~3K patients), chronic opioid use ranged ~50-67%
11

## Slide 13
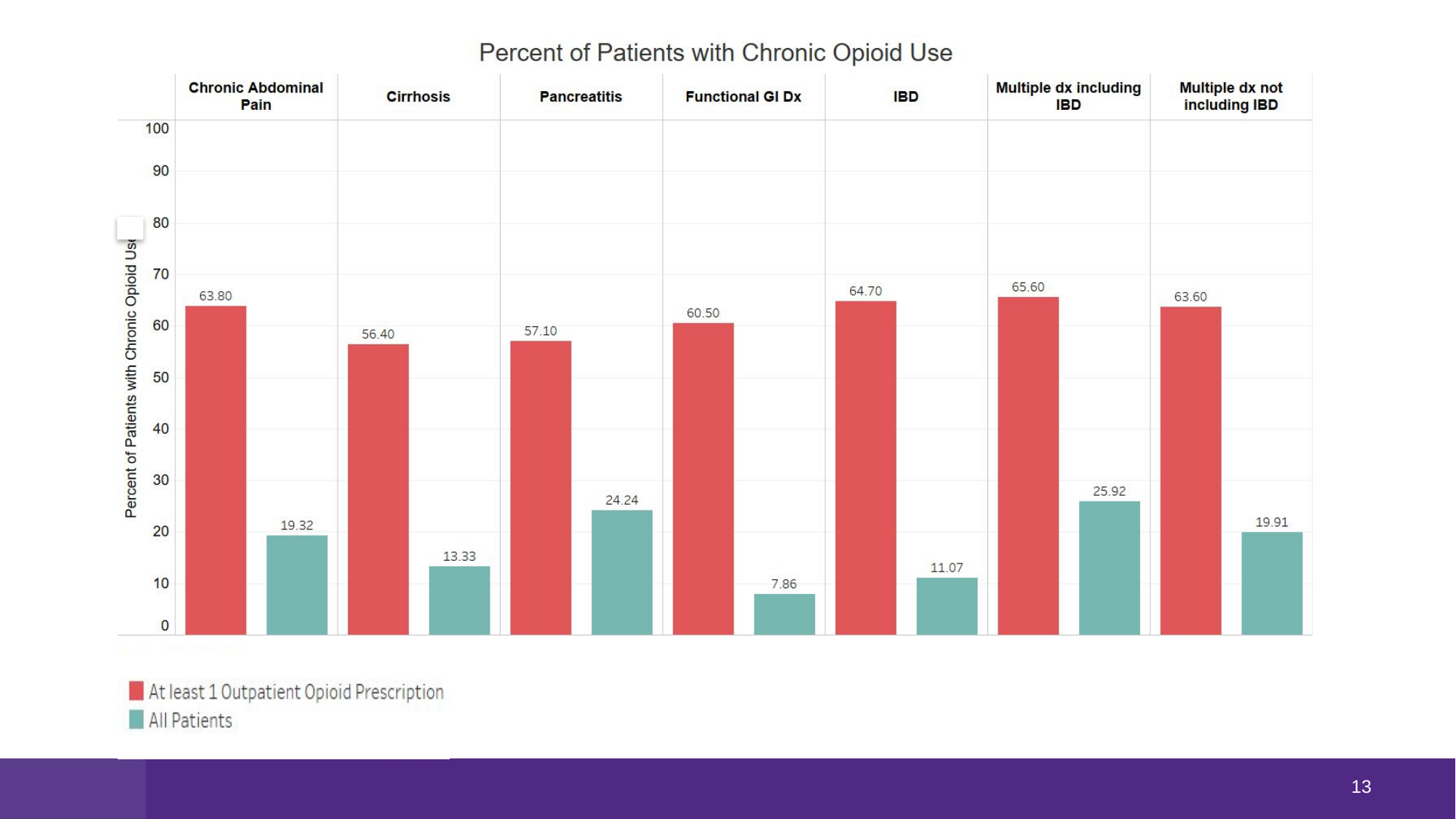

12

## Slide 14
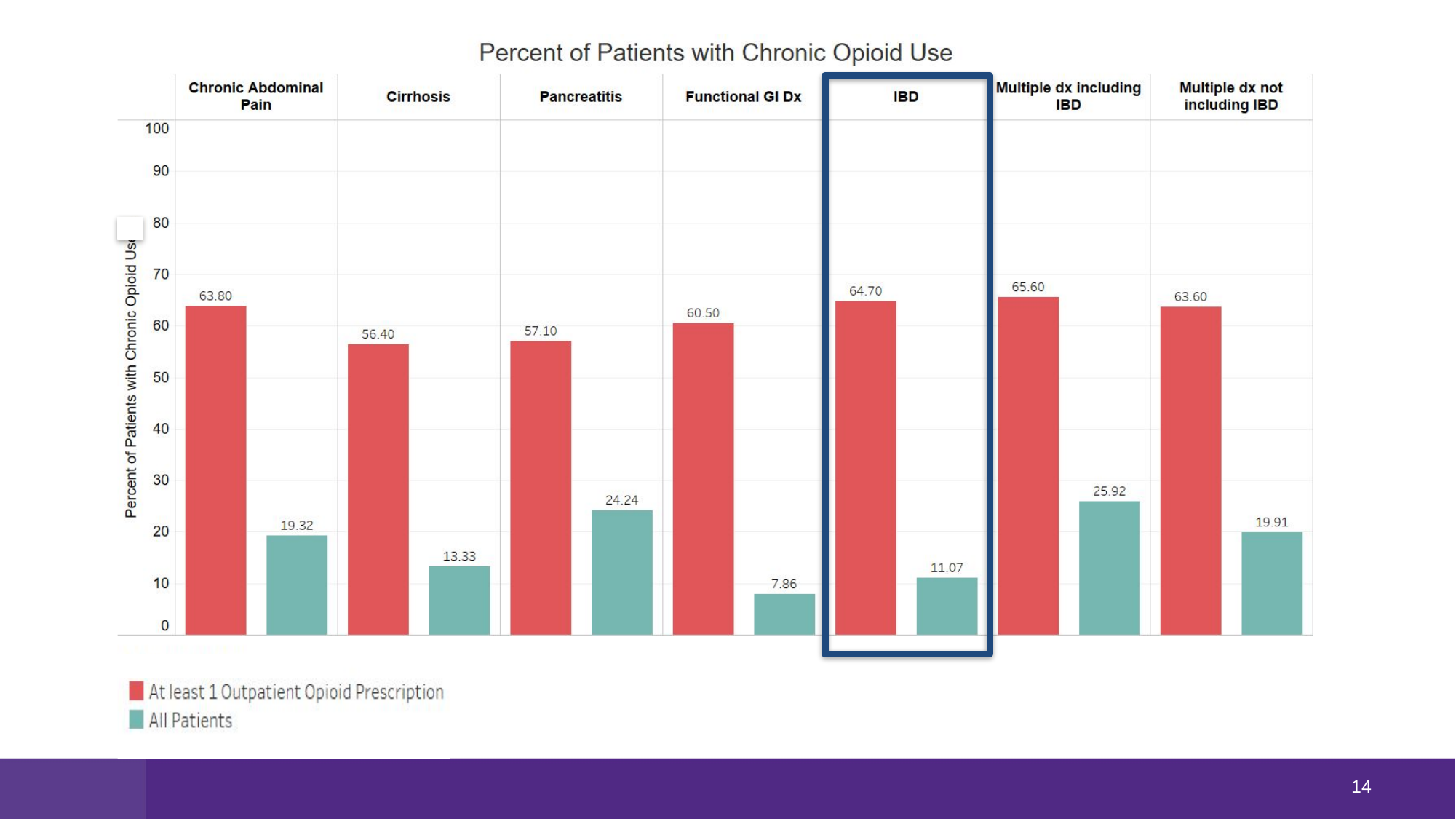

13

## Slide 15
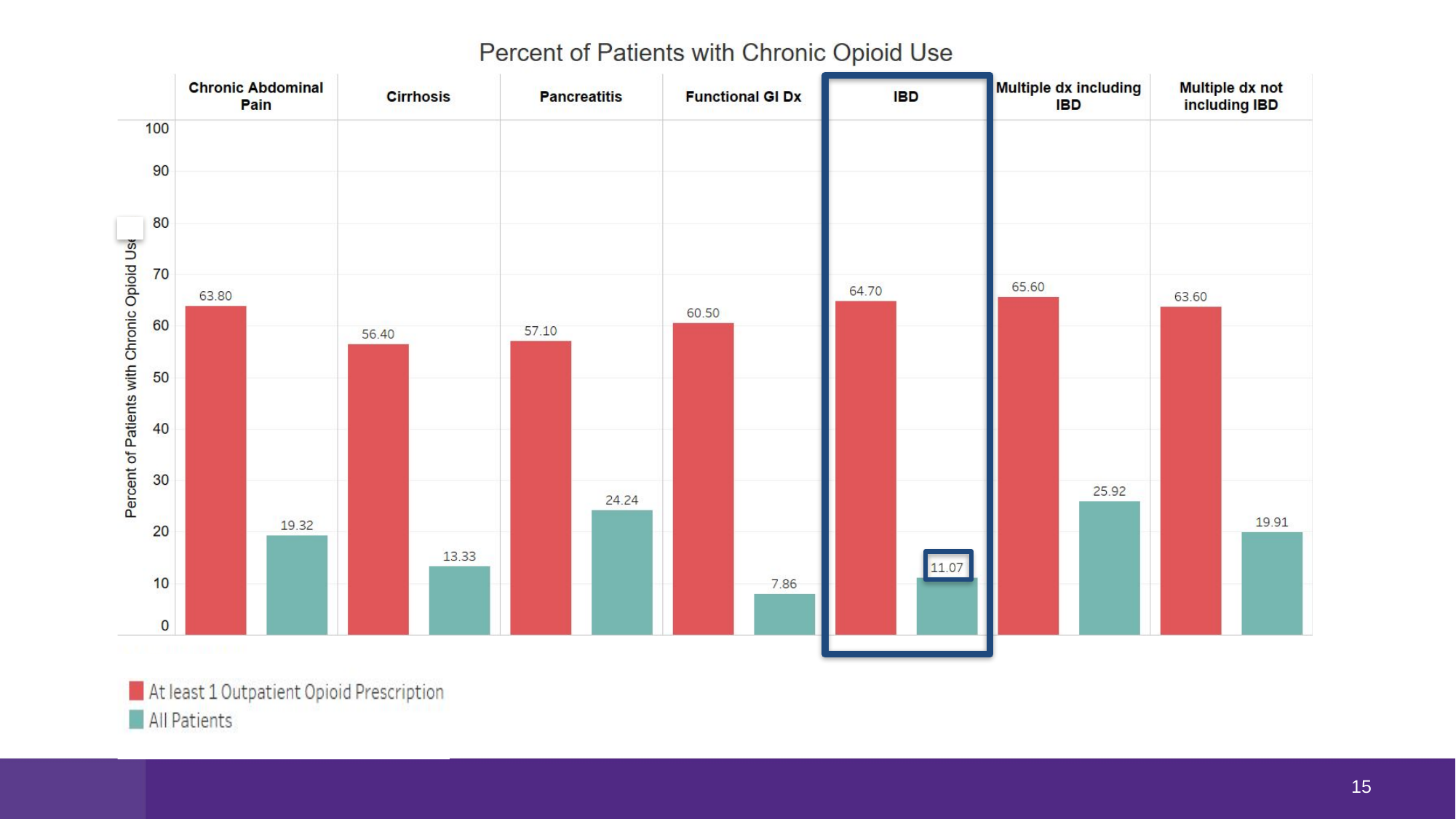

14

## Slide 16
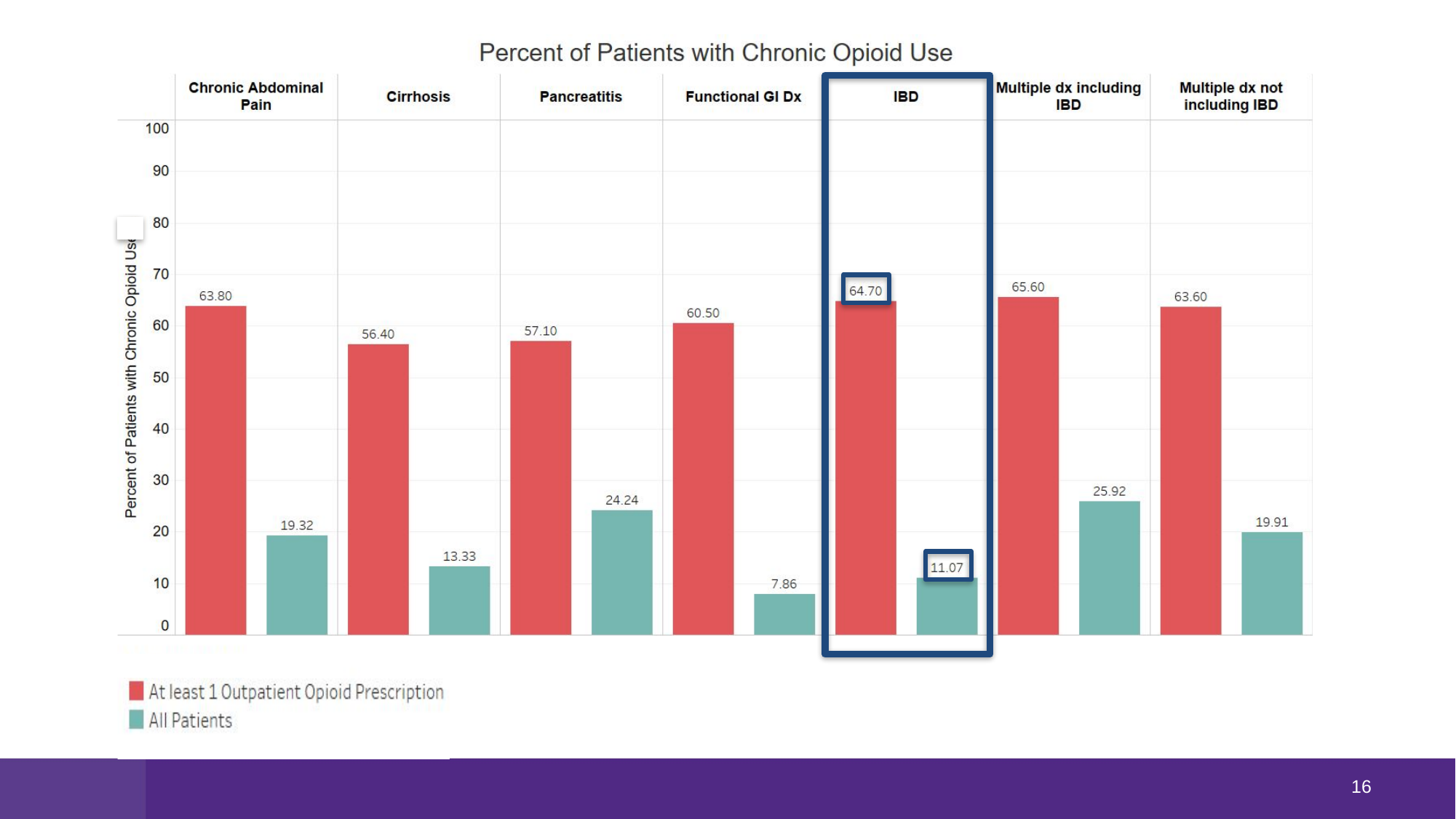

15

## Slide 17
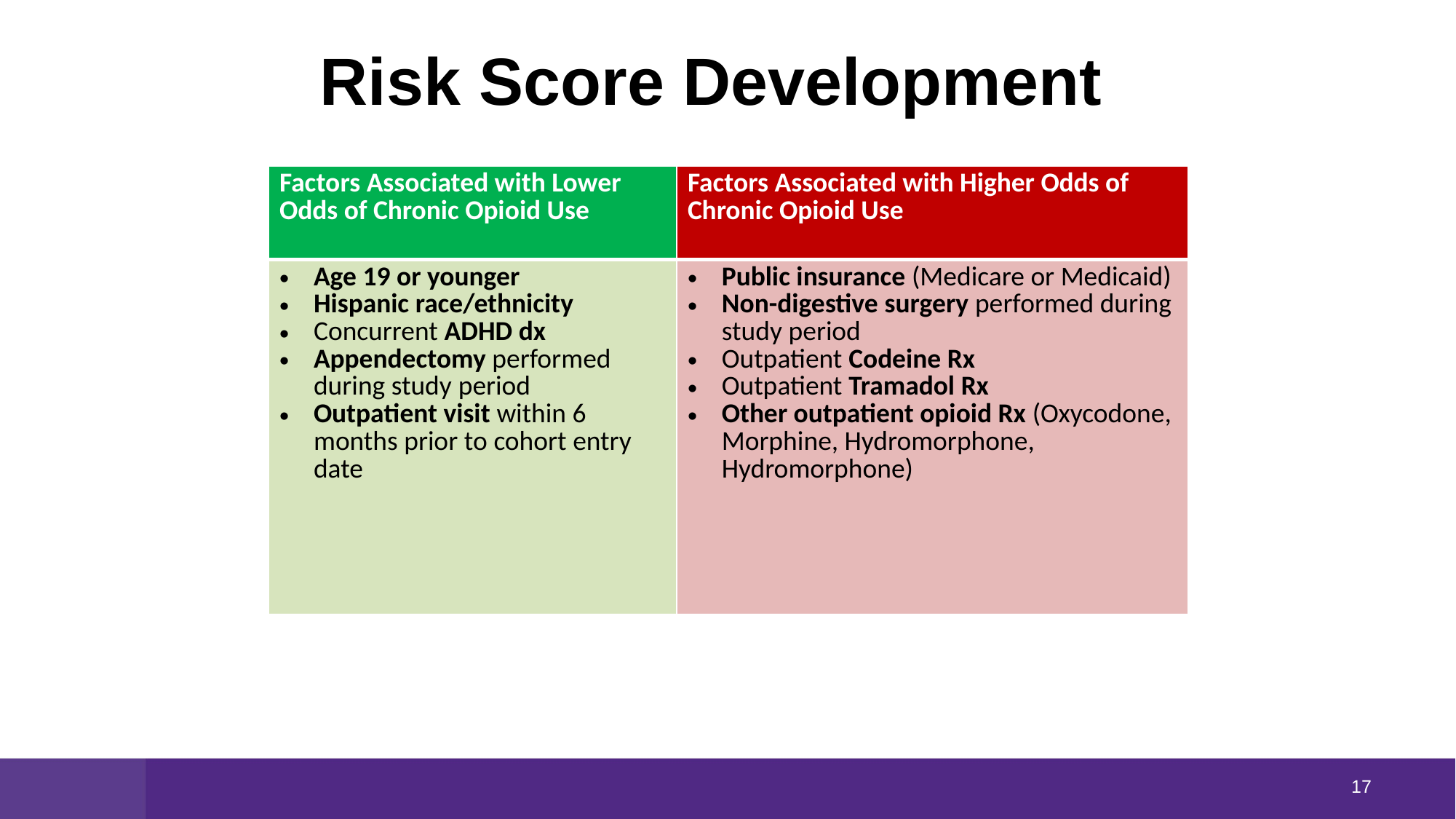

Risk Score Development
| Factors Associated with Lower Odds of Chronic Opioid Use | Factors Associated with Higher Odds of Chronic Opioid Use |
| --- | --- |
| Age 19 or younger  Hispanic race/ethnicity Concurrent ADHD dx Appendectomy performed during study period Outpatient visit within 6 months prior to cohort entry date | Public insurance (Medicare or Medicaid) Non-digestive surgery performed during study period Outpatient Codeine Rx Outpatient Tramadol Rx Other outpatient opioid Rx (Oxycodone, Morphine, Hydromorphone,  Hydromorphone) |
16

## Slide 18
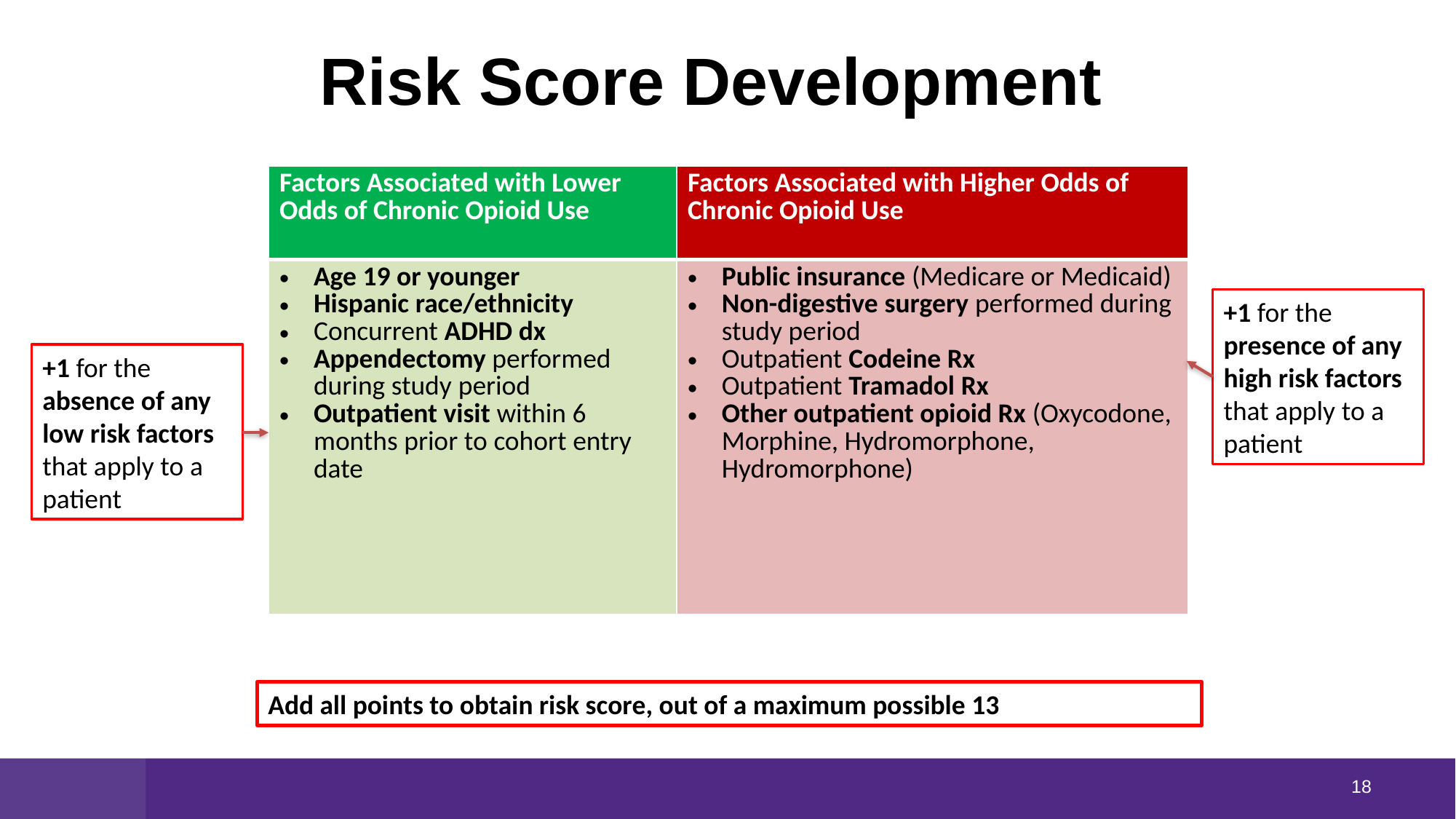

Risk Score Development
| Factors Associated with Lower Odds of Chronic Opioid Use | Factors Associated with Higher Odds of Chronic Opioid Use |
| --- | --- |
| Age 19 or younger  Hispanic race/ethnicity Concurrent ADHD dx Appendectomy performed during study period Outpatient visit within 6 months prior to cohort entry date | Public insurance (Medicare or Medicaid) Non-digestive surgery performed during study period Outpatient Codeine Rx Outpatient Tramadol Rx Other outpatient opioid Rx (Oxycodone, Morphine, Hydromorphone,  Hydromorphone) |
+1 for the presence of any high risk factors that apply to a patient
+1 for the absence of any low risk factors that apply to a patient
Add all points to obtain risk score, out of a maximum possible 13
17

## Slide 19
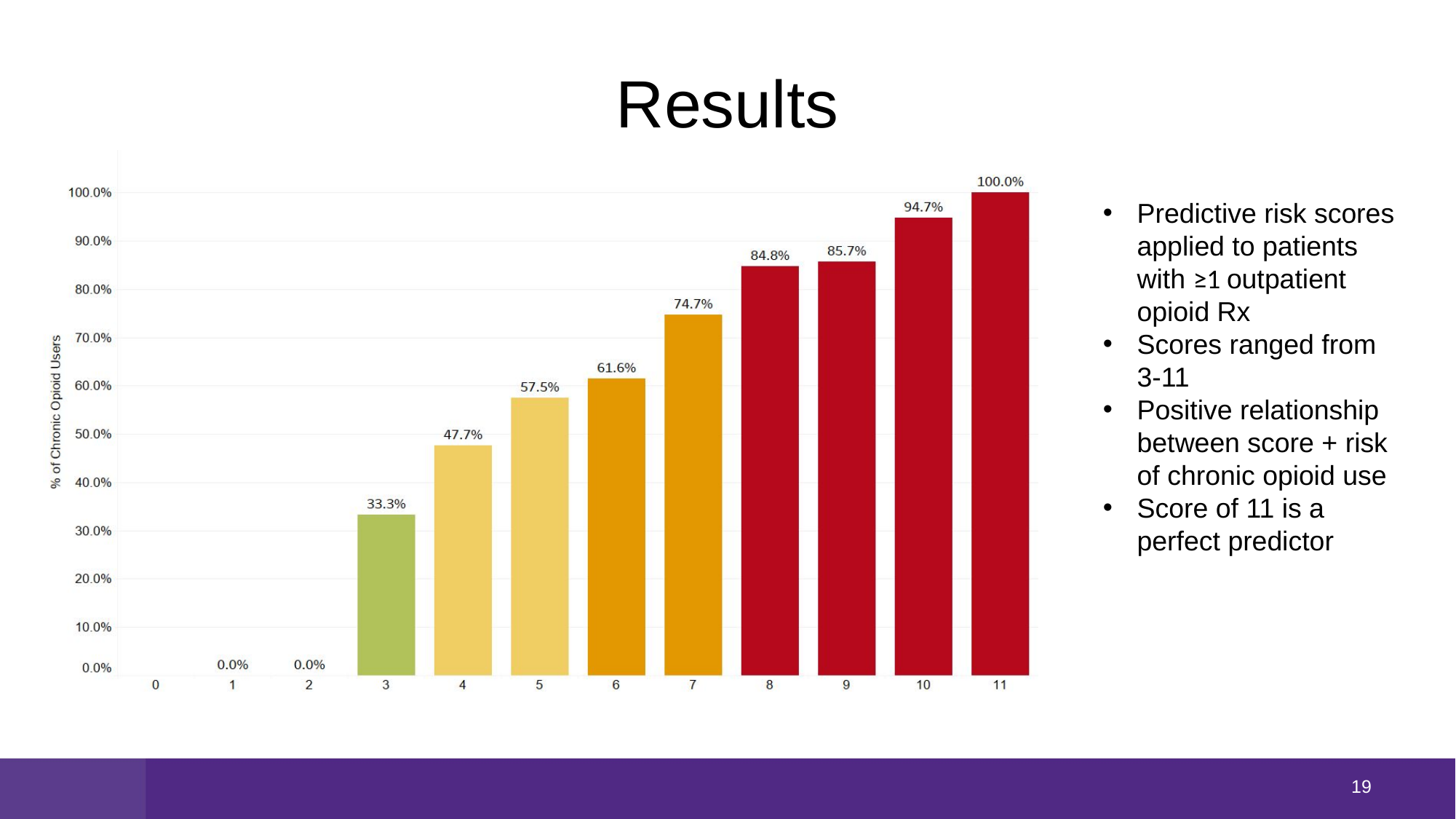

# Results
Predictive risk scores applied to patients with ≥1 outpatient opioid Rx
Scores ranged from 3-11
Positive relationship between score + risk of chronic opioid use
Score of 11 is a perfect predictor
18

## Slide 20
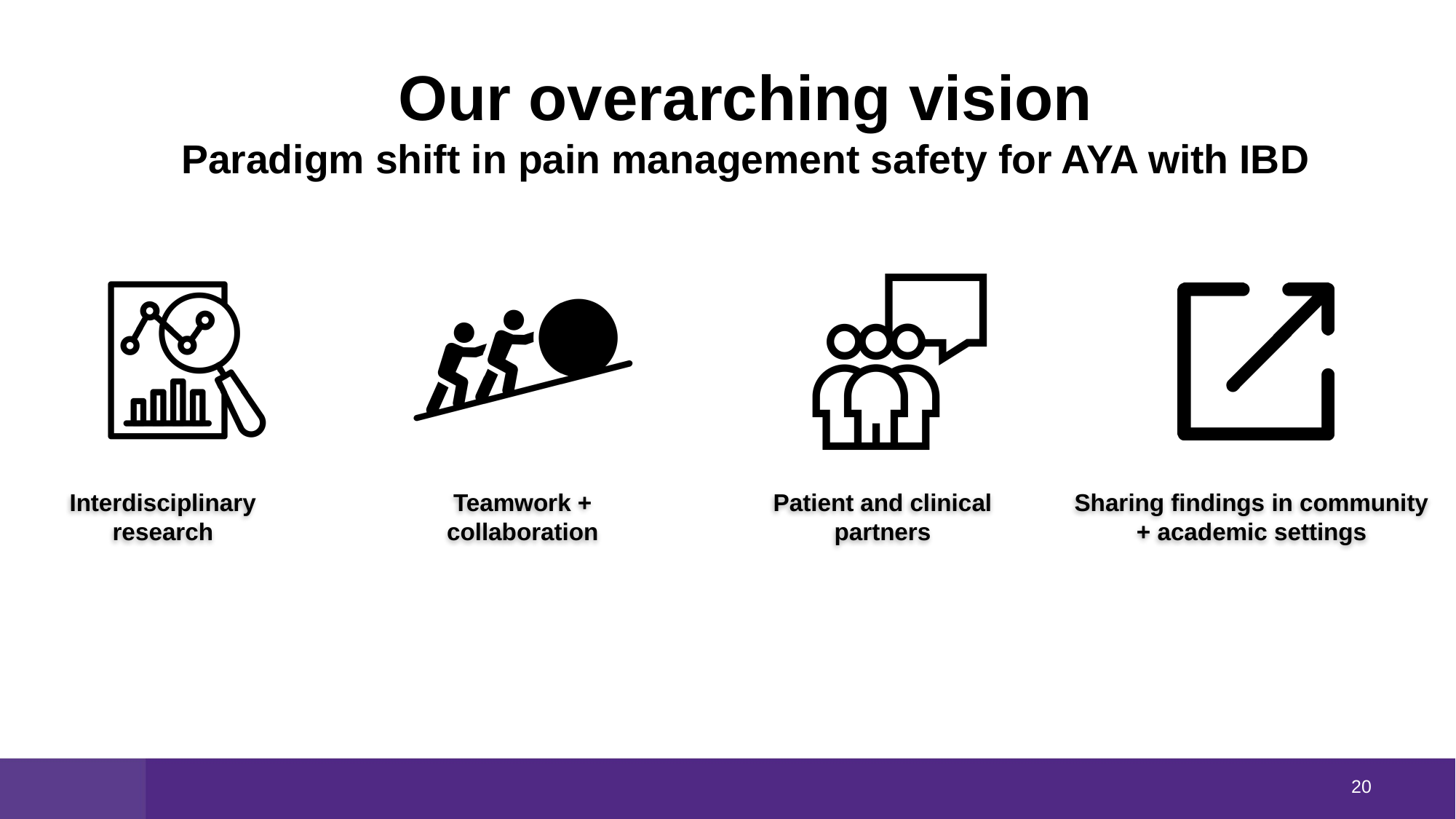

Our overarching vision
Paradigm shift in pain management safety for AYA with IBD
Interdisciplinary research
Teamwork + collaboration
Patient and clinical partners
Sharing findings in community + academic settings
19

## Slide 21
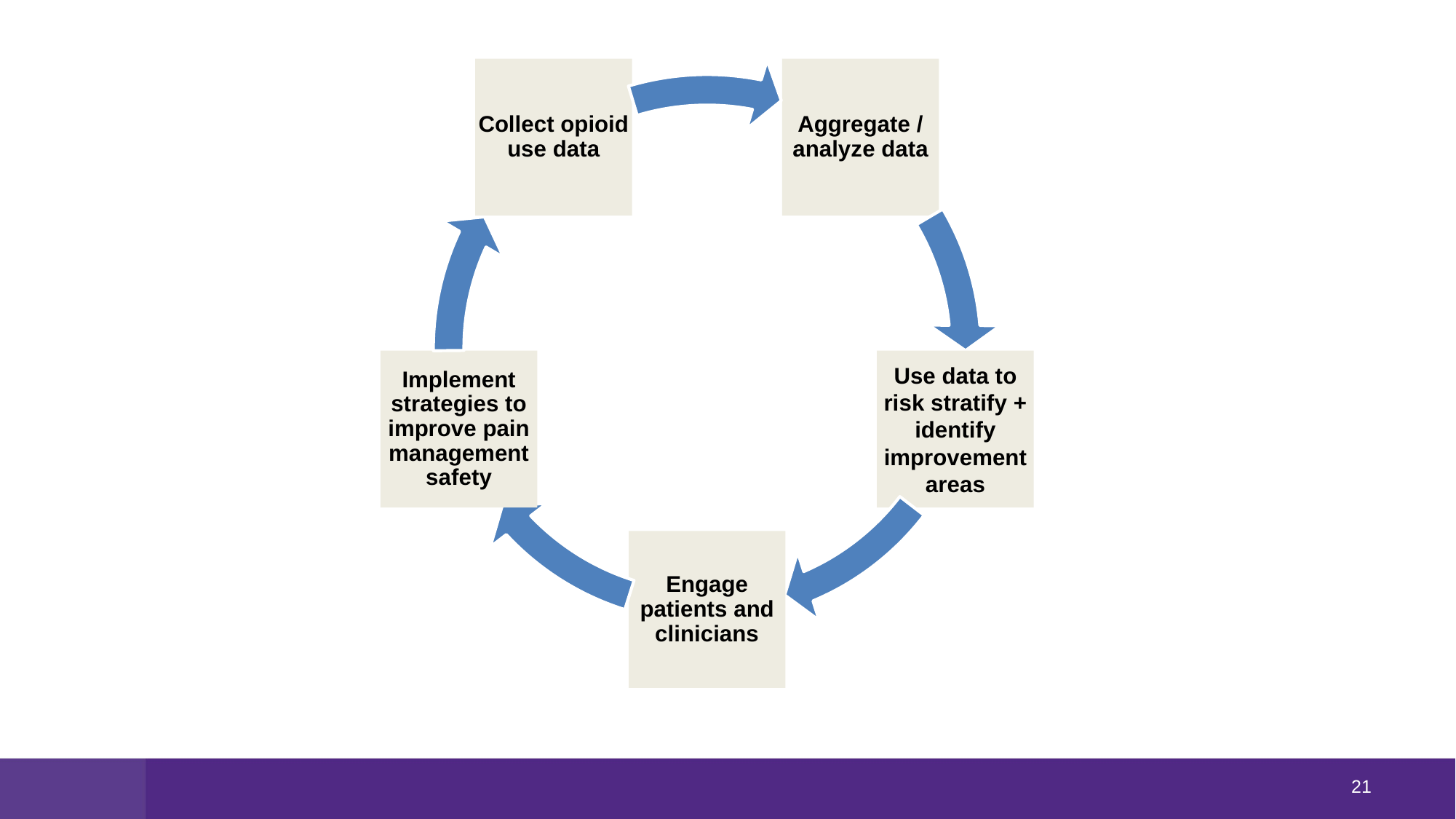

20

## Slide 22
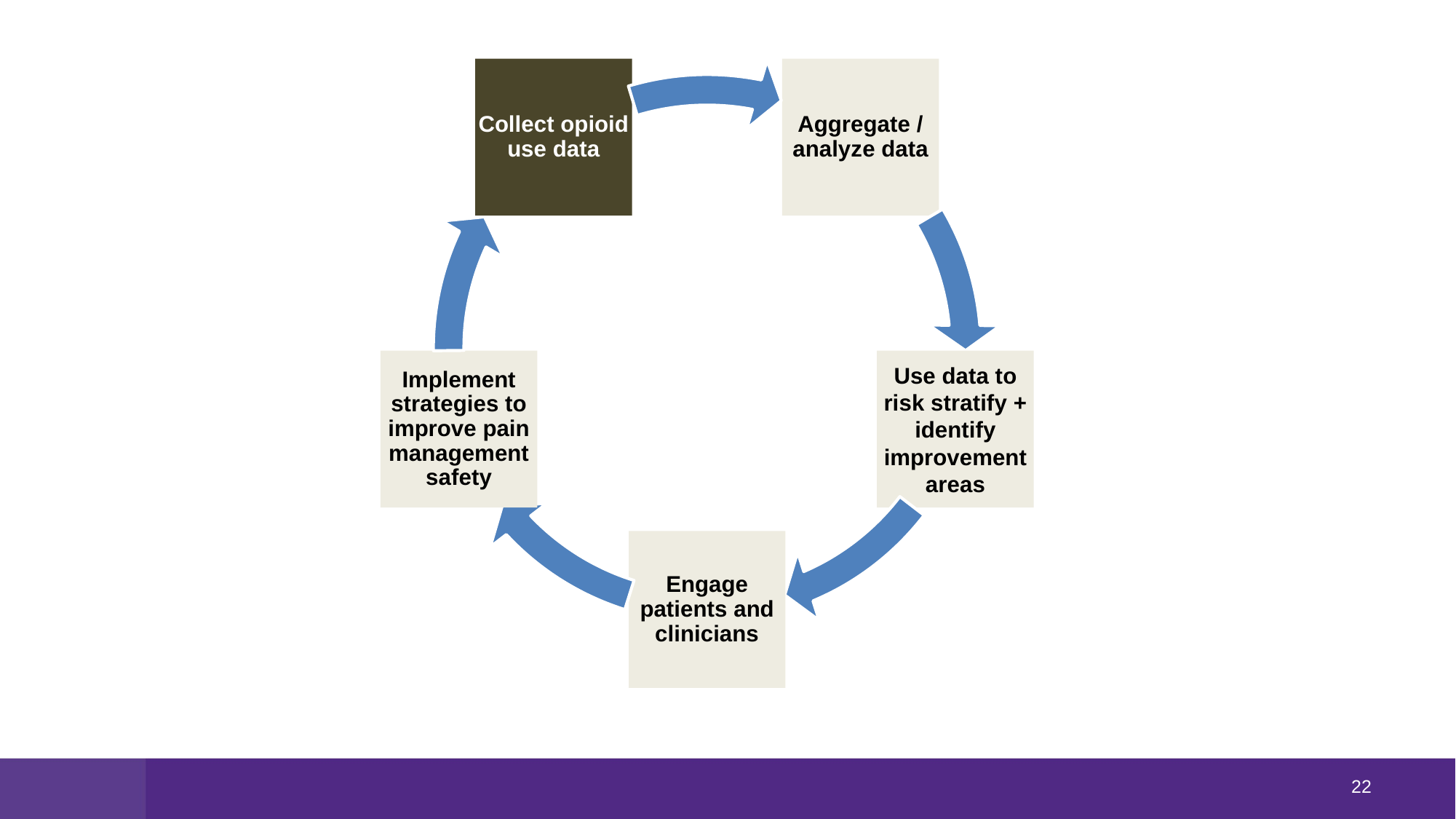

21

## Slide 23
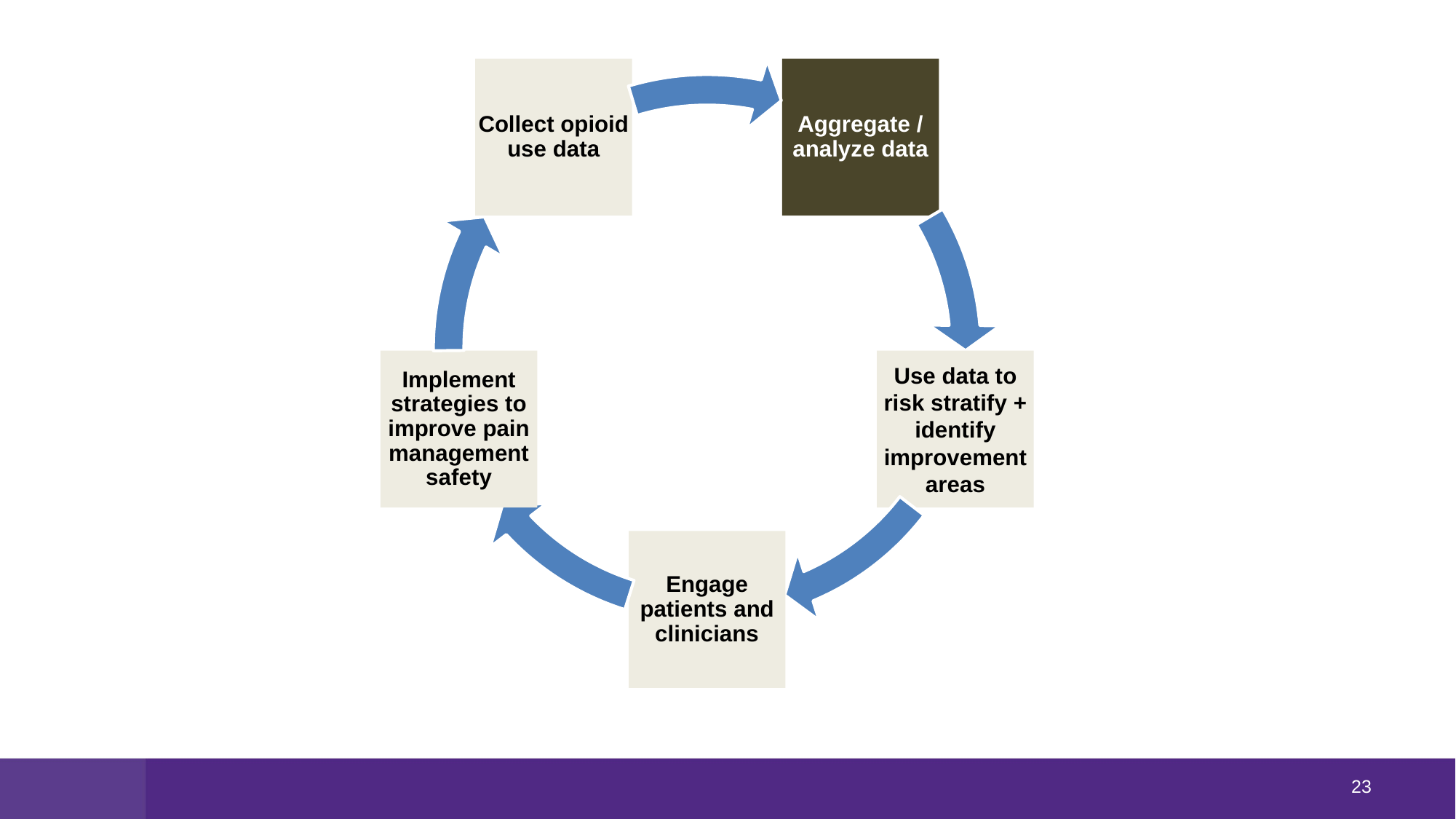

22

## Slide 24
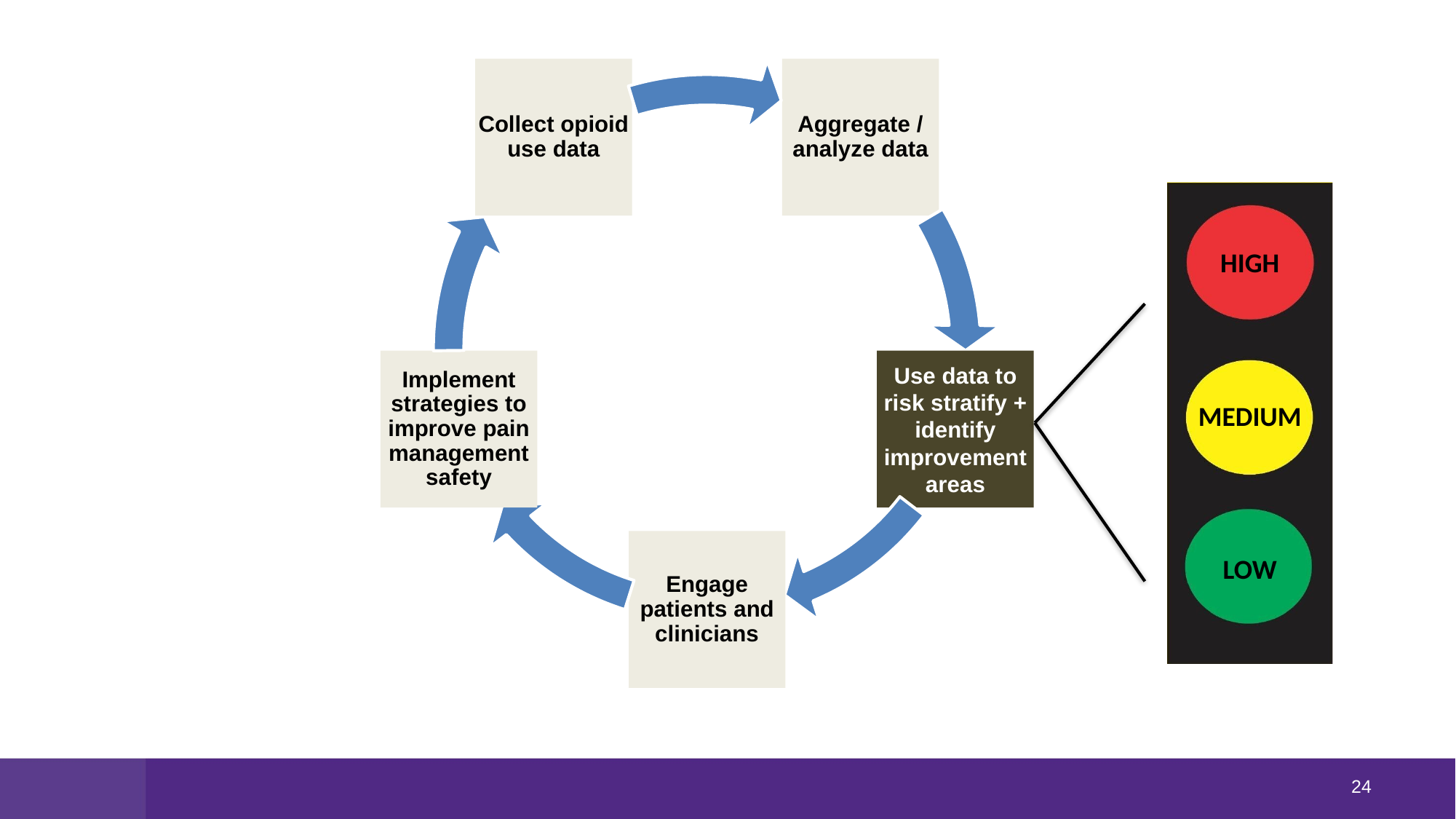

HIGH
MEDIUM
LOW
23

## Slide 25
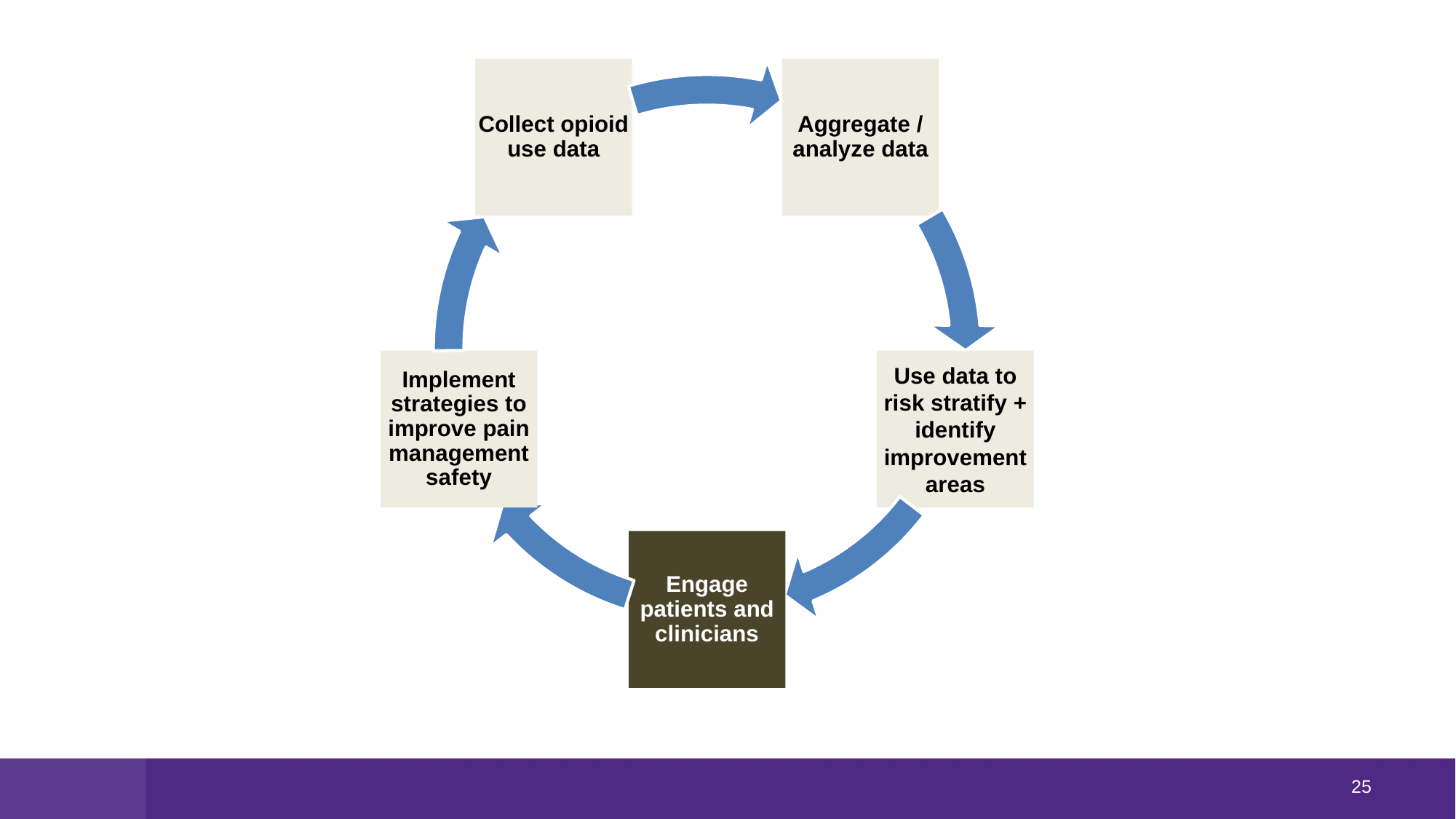

24

## Slide 26
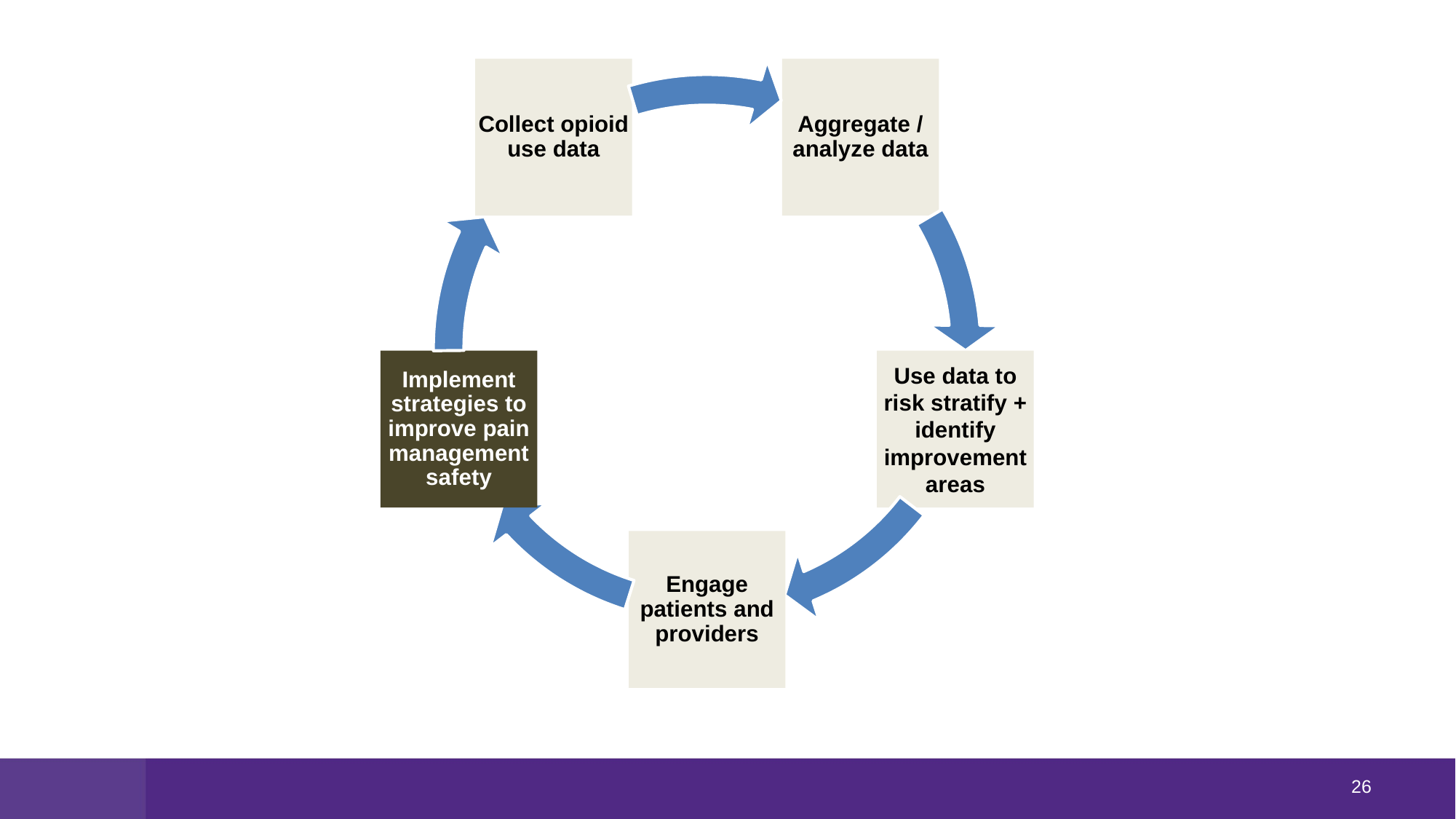

25

## Slide 27
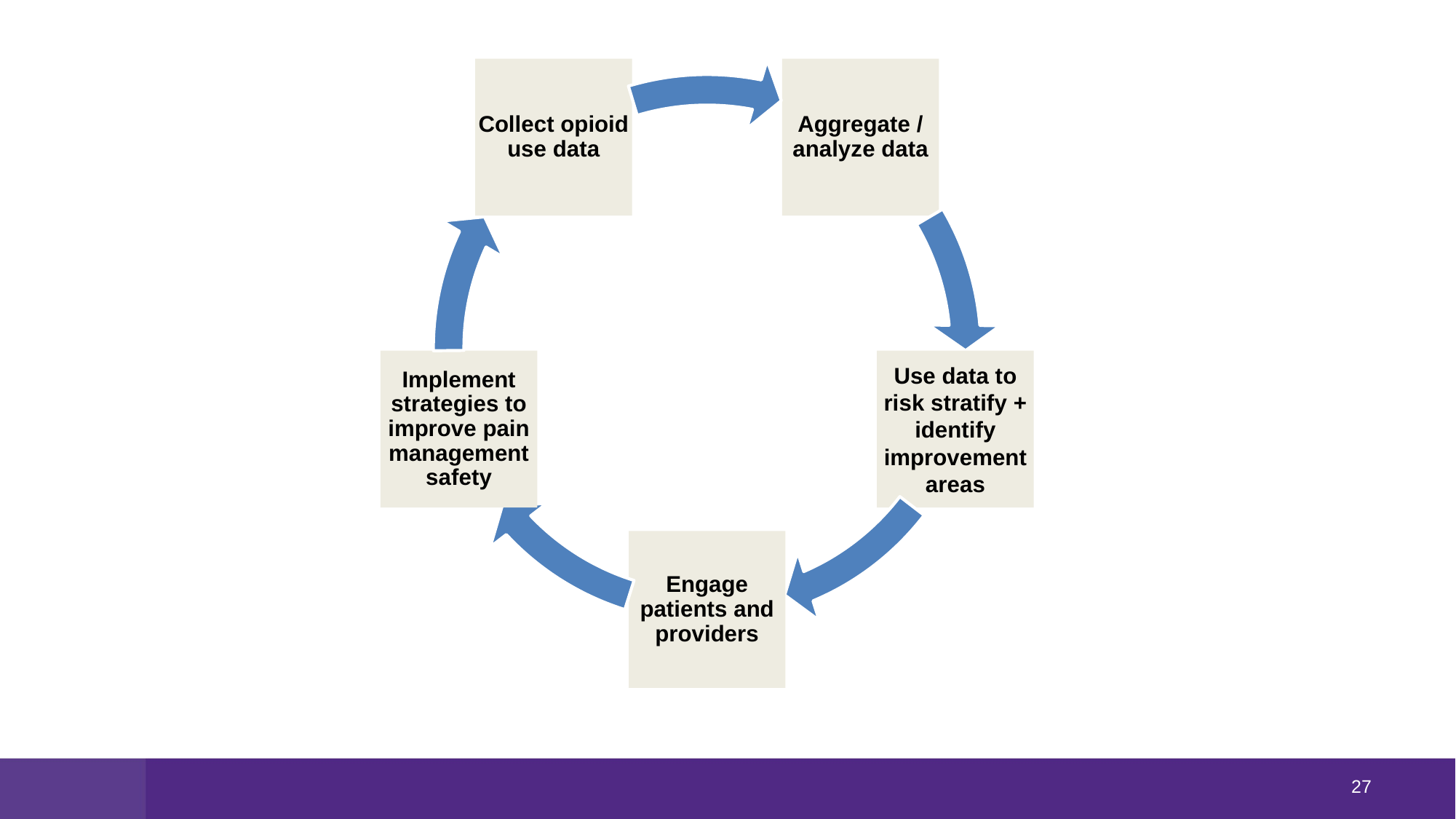

26

## Slide 28
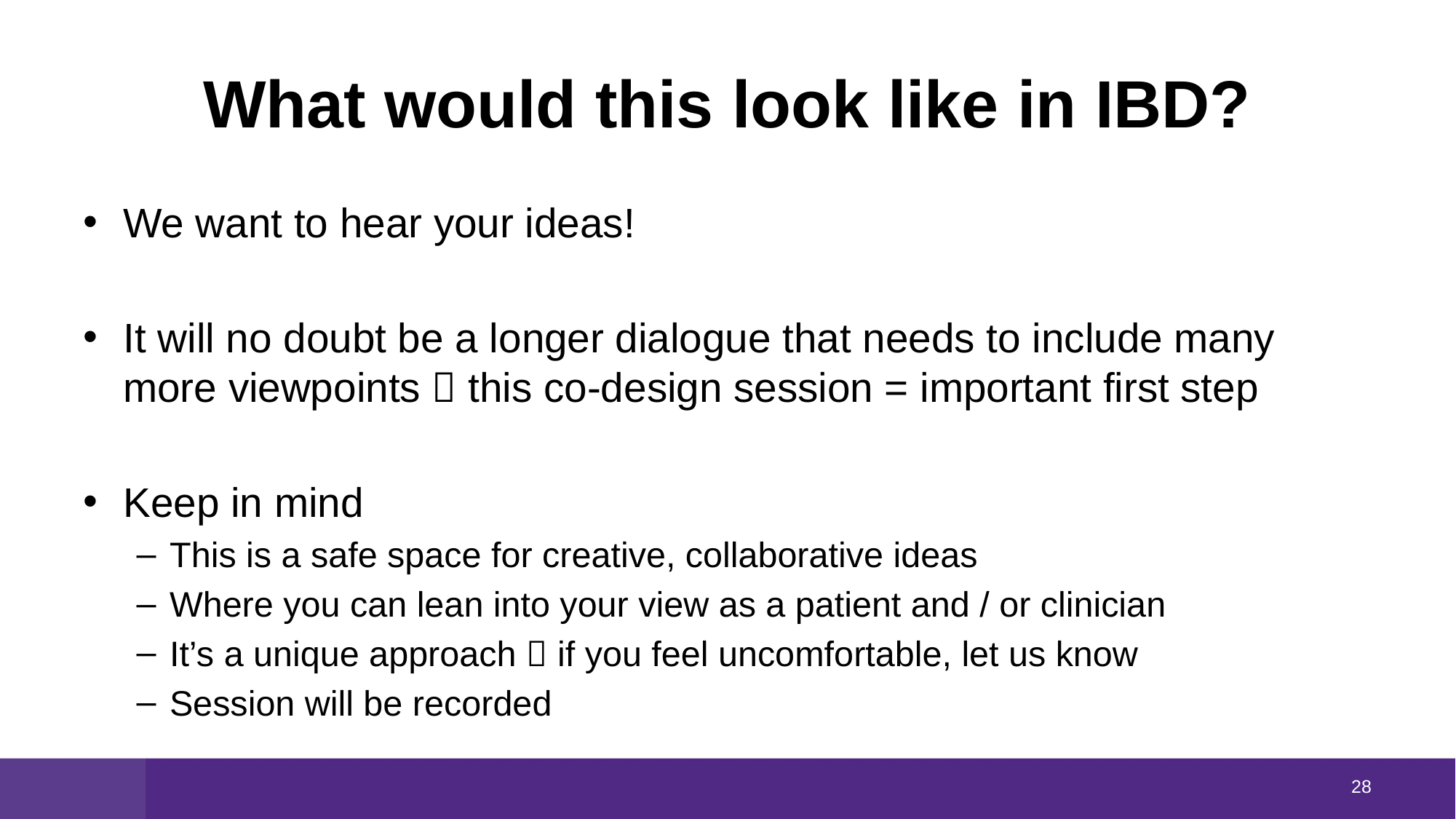

# What would this look like in IBD?
We want to hear your ideas!
It will no doubt be a longer dialogue that needs to include many more viewpoints  this co-design session = important first step
Keep in mind
This is a safe space for creative, collaborative ideas
Where you can lean into your view as a patient and / or clinician
It’s a unique approach  if you feel uncomfortable, let us know
Session will be recorded
27

## Slide 29
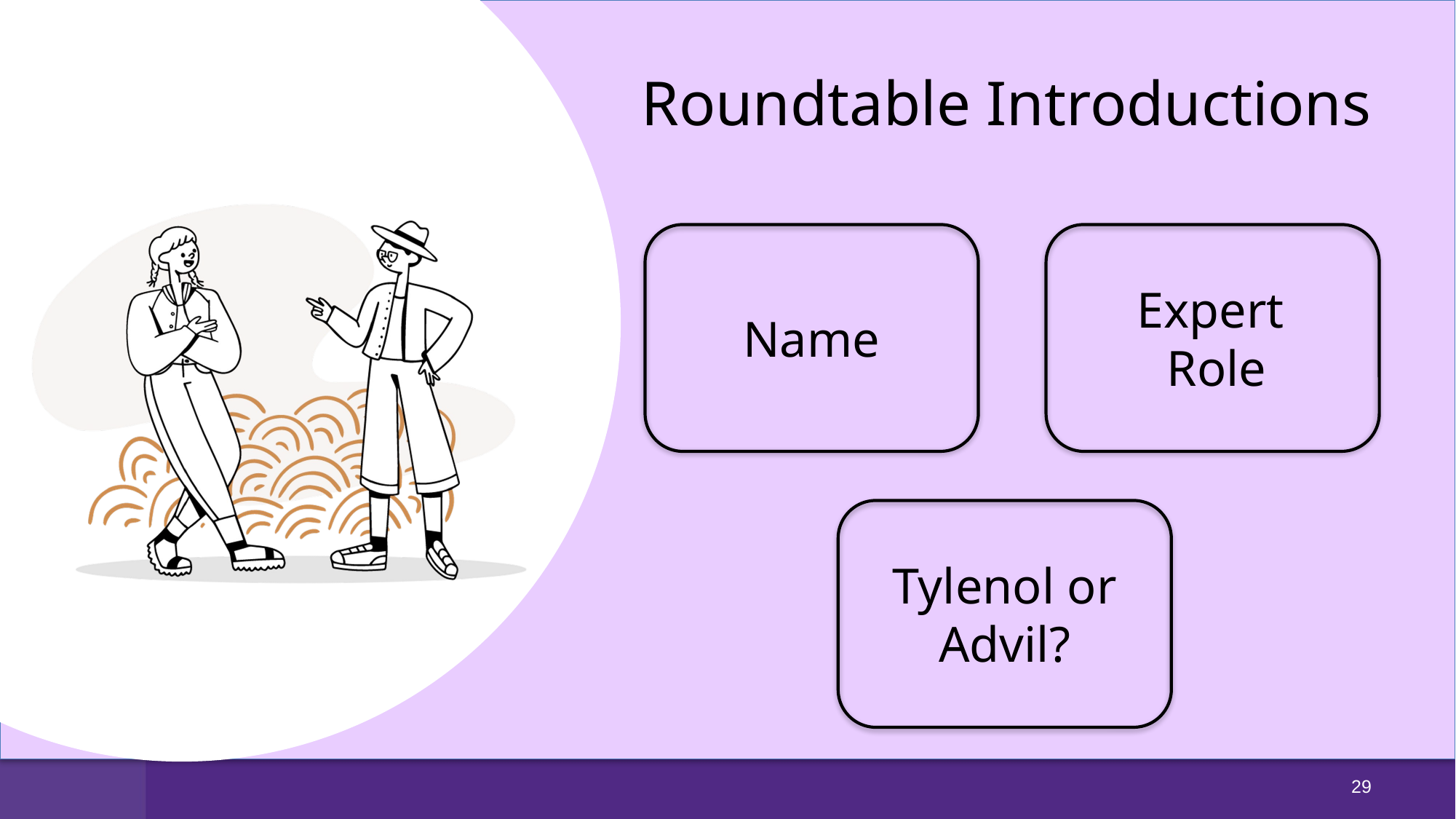

# Roundtable Introductions
Expert
Role
Name
Tylenol or Advil?
28

## Slide 30
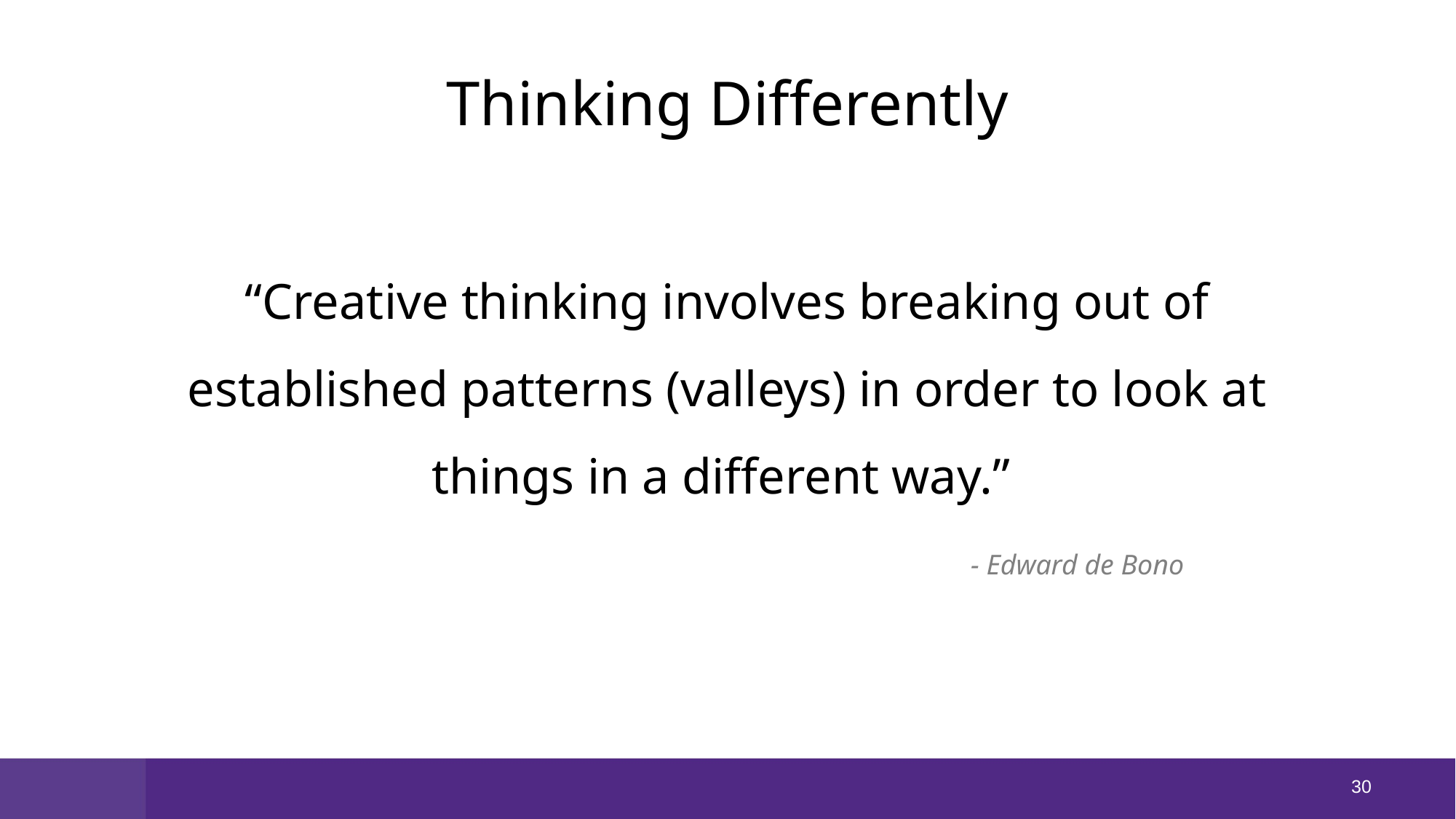

# Thinking Differently
“Creative thinking involves breaking out of established patterns (valleys) in order to look at things in a different way.”
- Edward de Bono
29

## Slide 31
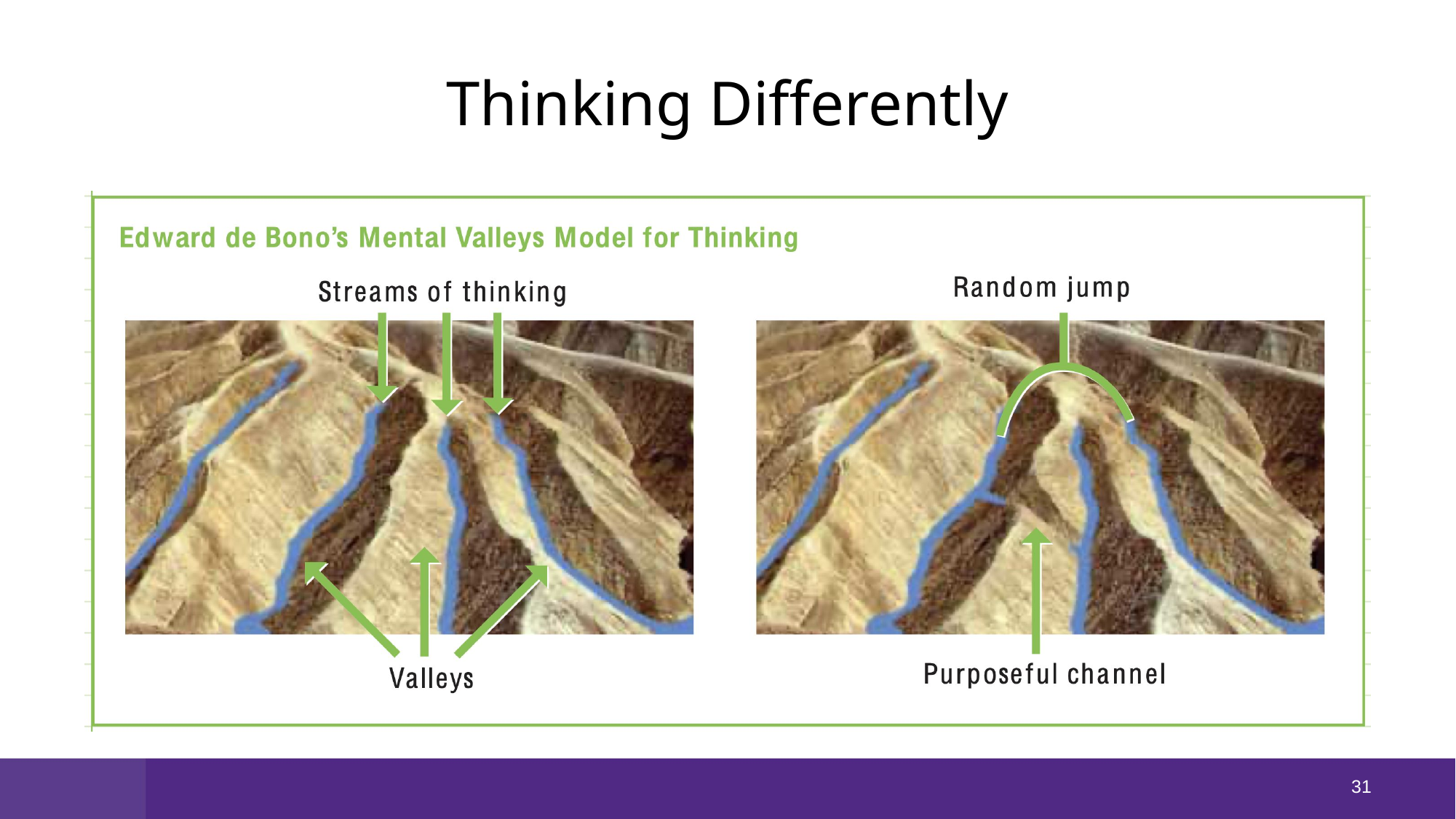

# Thinking Differently
30

## Slide 32
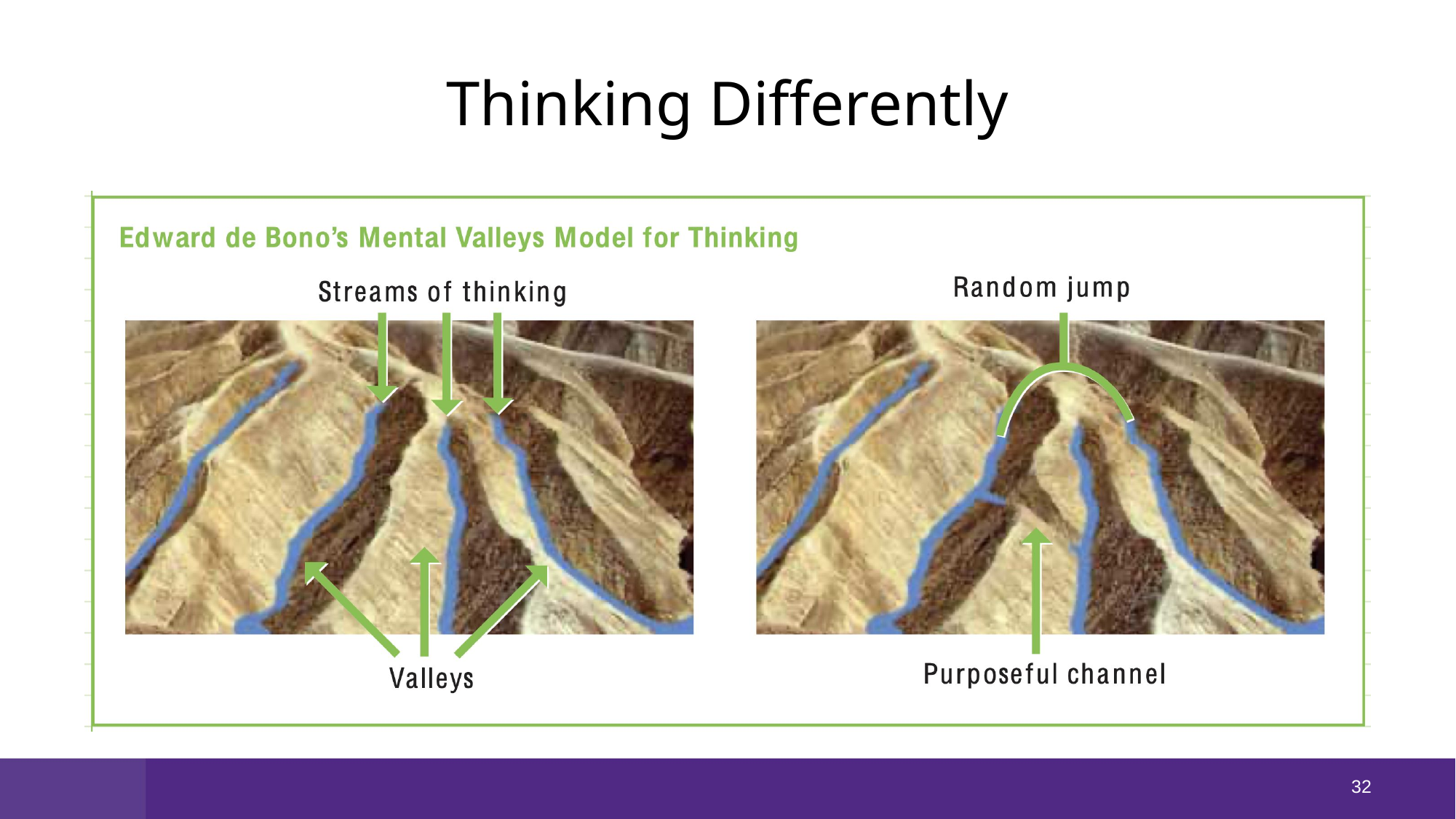

# Thinking Differently
31

## Slide 33
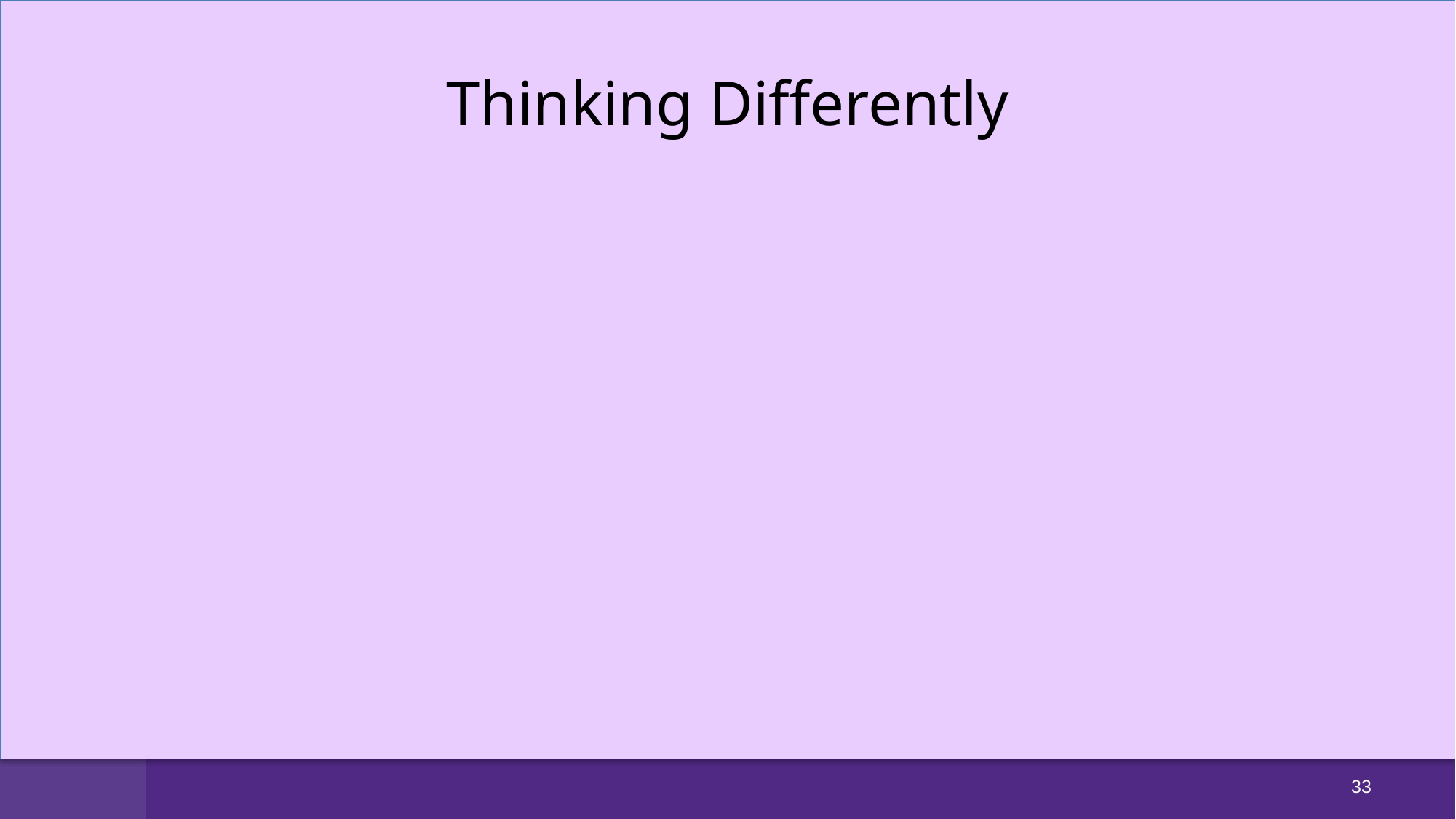

# Thinking Differently
32

## Slide 34
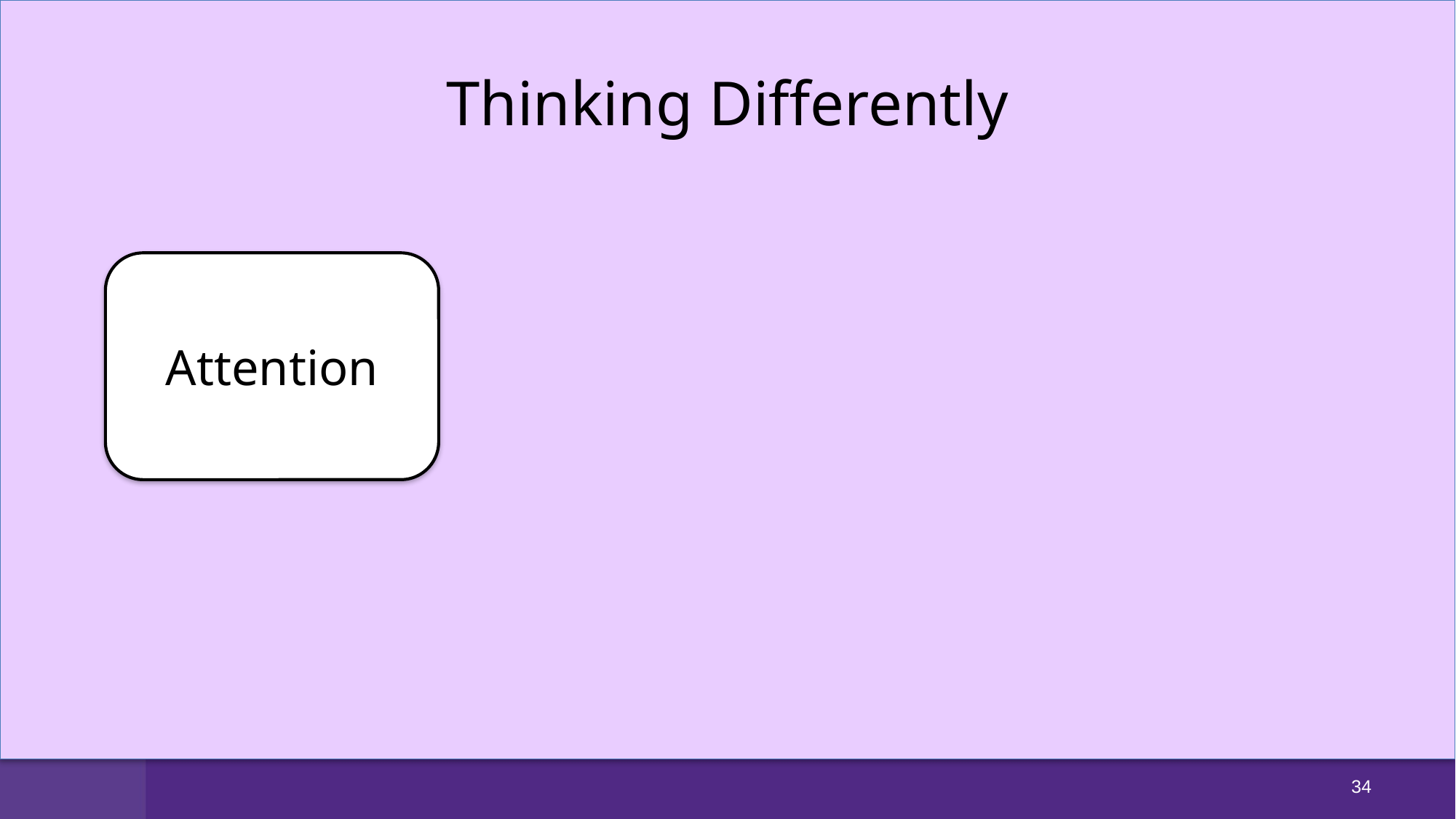

# Thinking Differently
Attention
33

## Slide 35
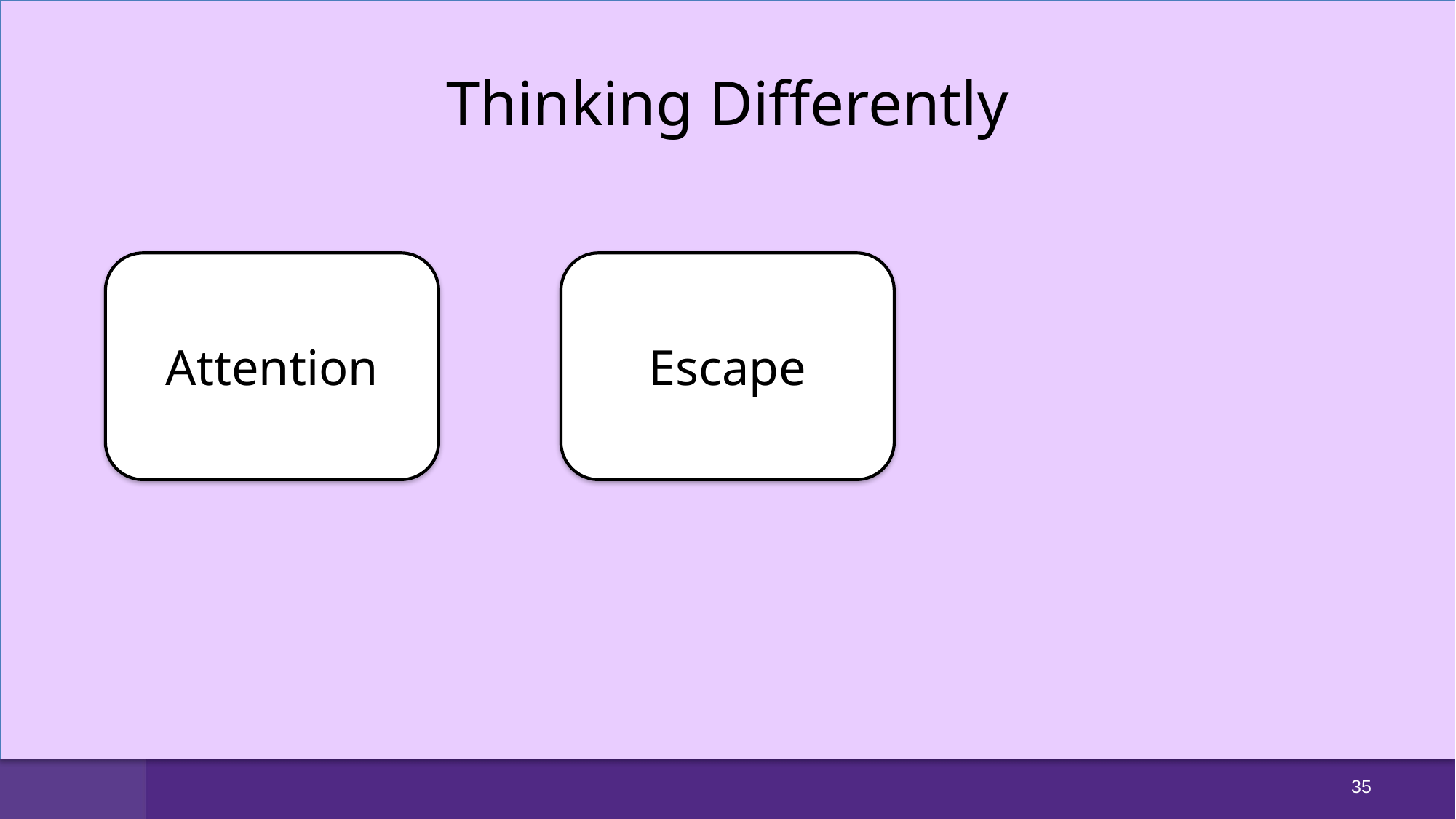

# Thinking Differently
Escape
Attention
34

## Slide 36
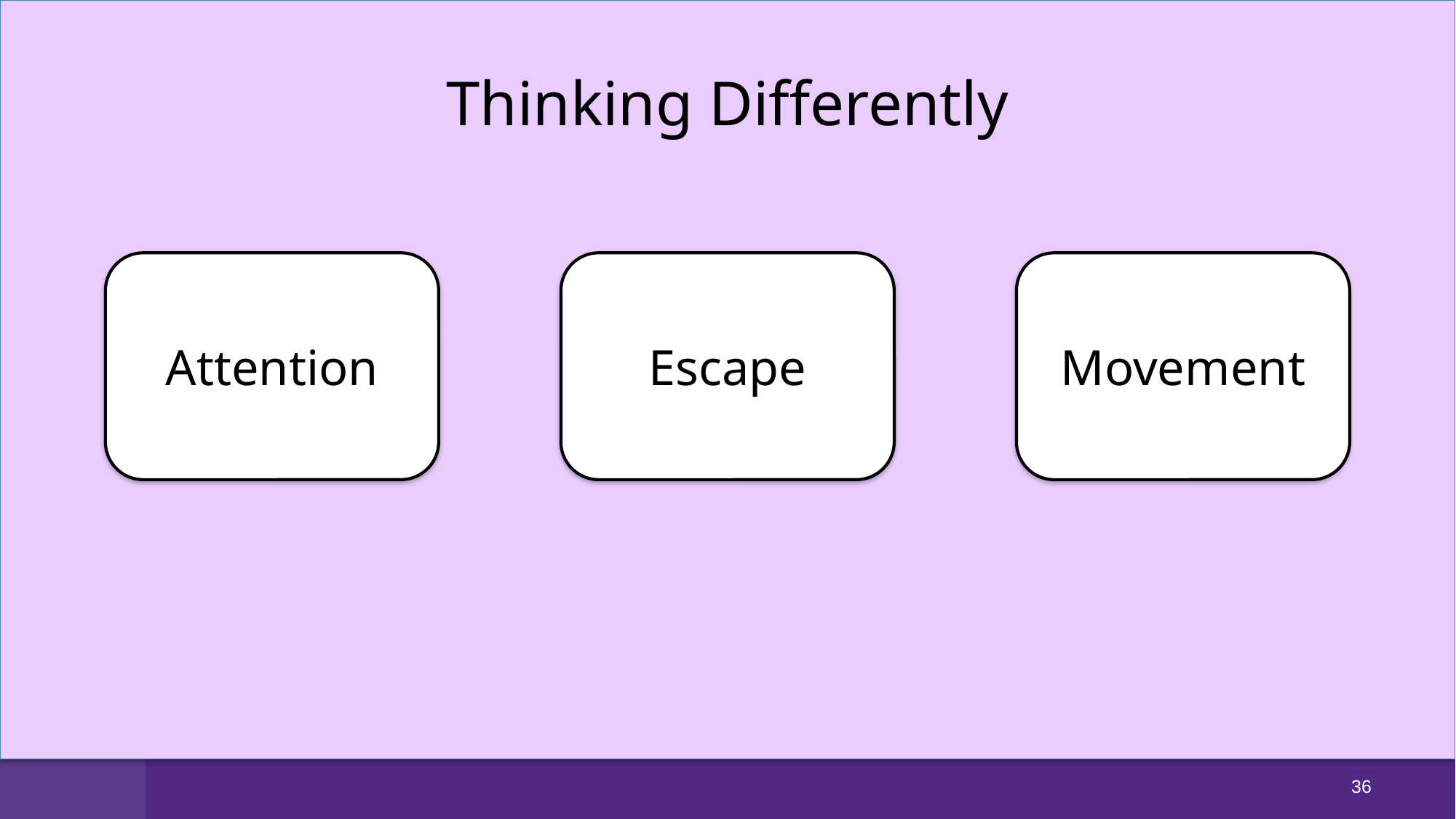

# Thinking Differently
Escape
Movement
Attention
35

## Slide 37
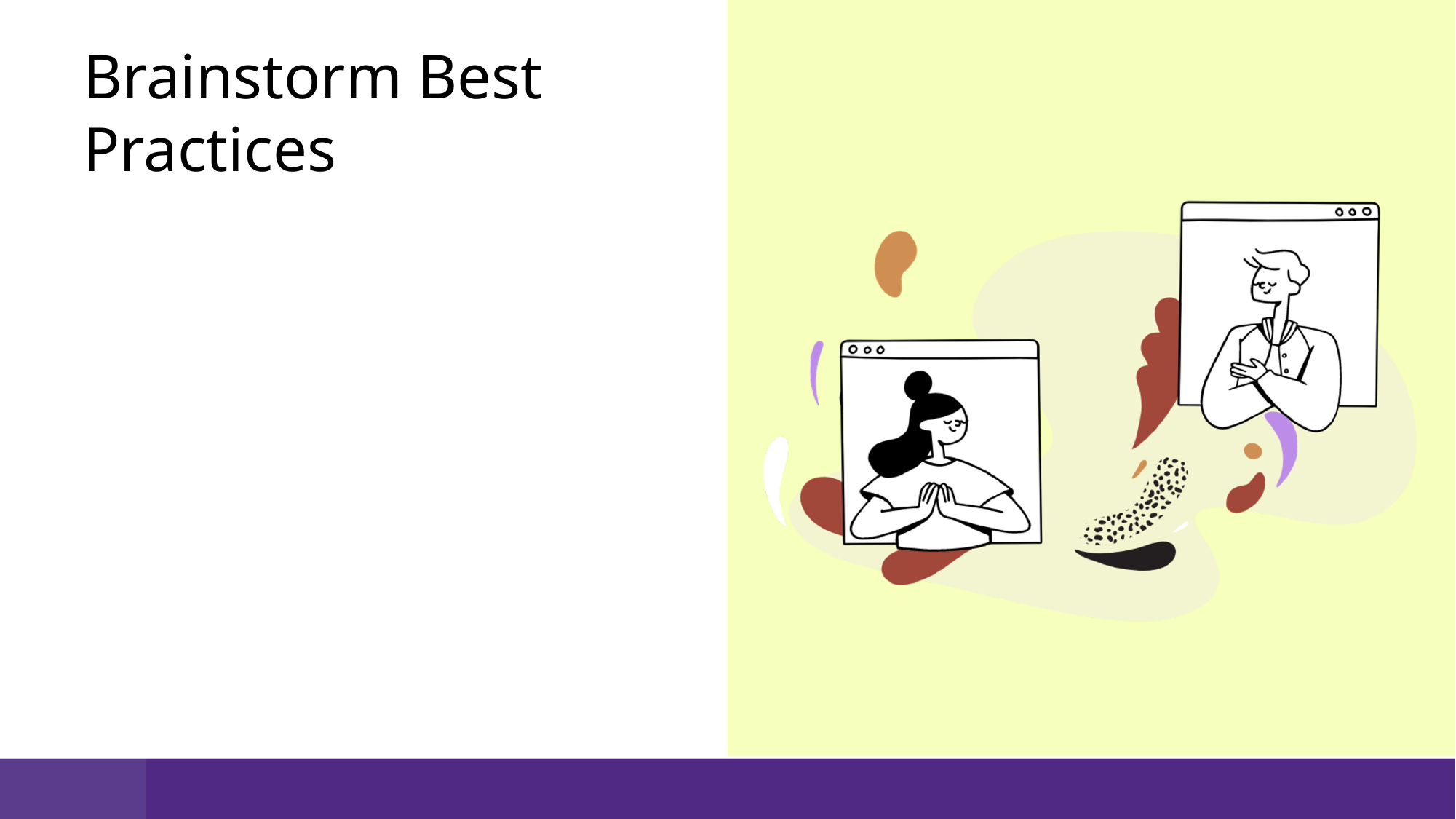

Brainstorm Best
Practices

## Slide 38
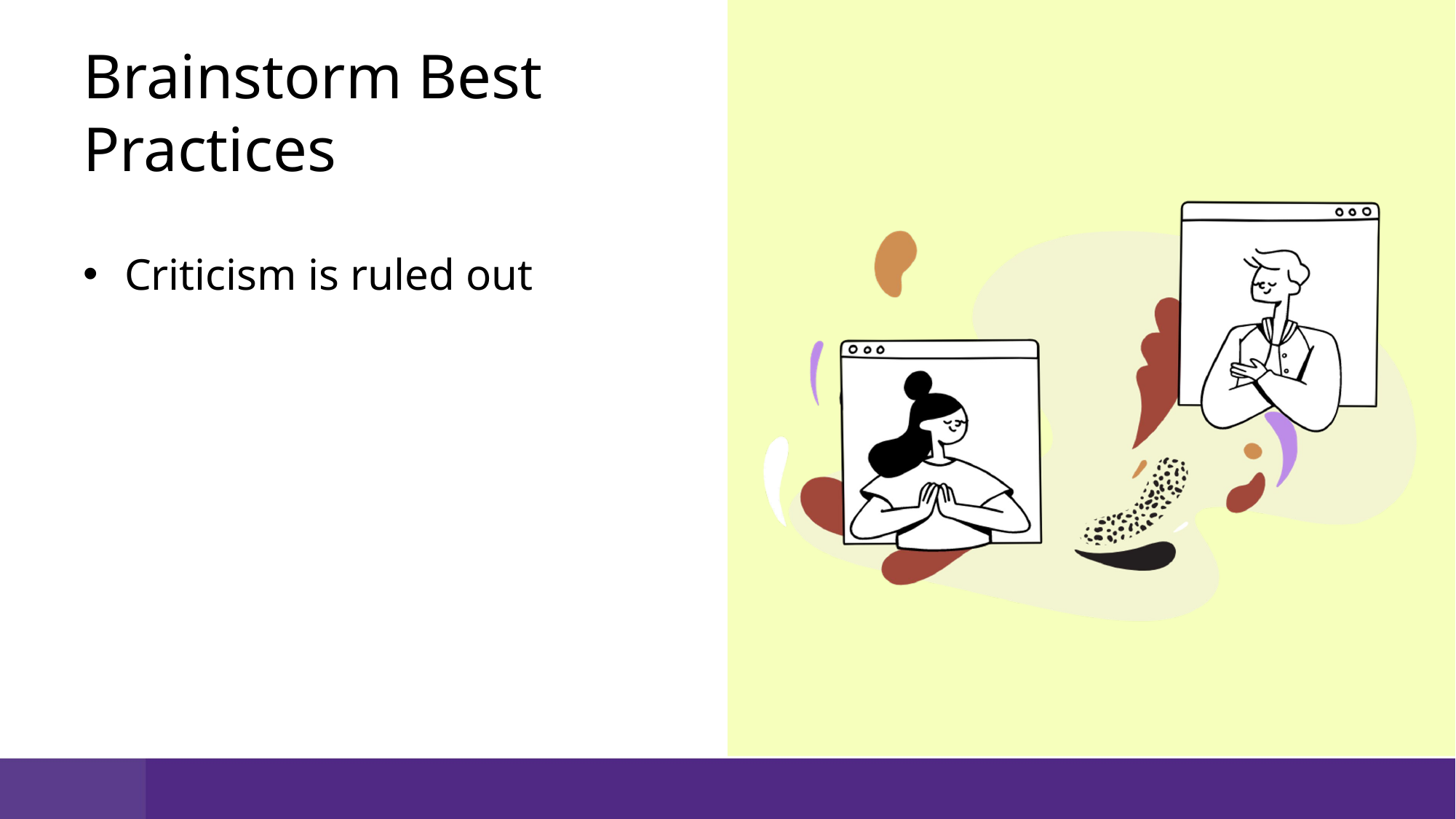

Brainstorm Best
Practices
Criticism is ruled out

## Slide 39
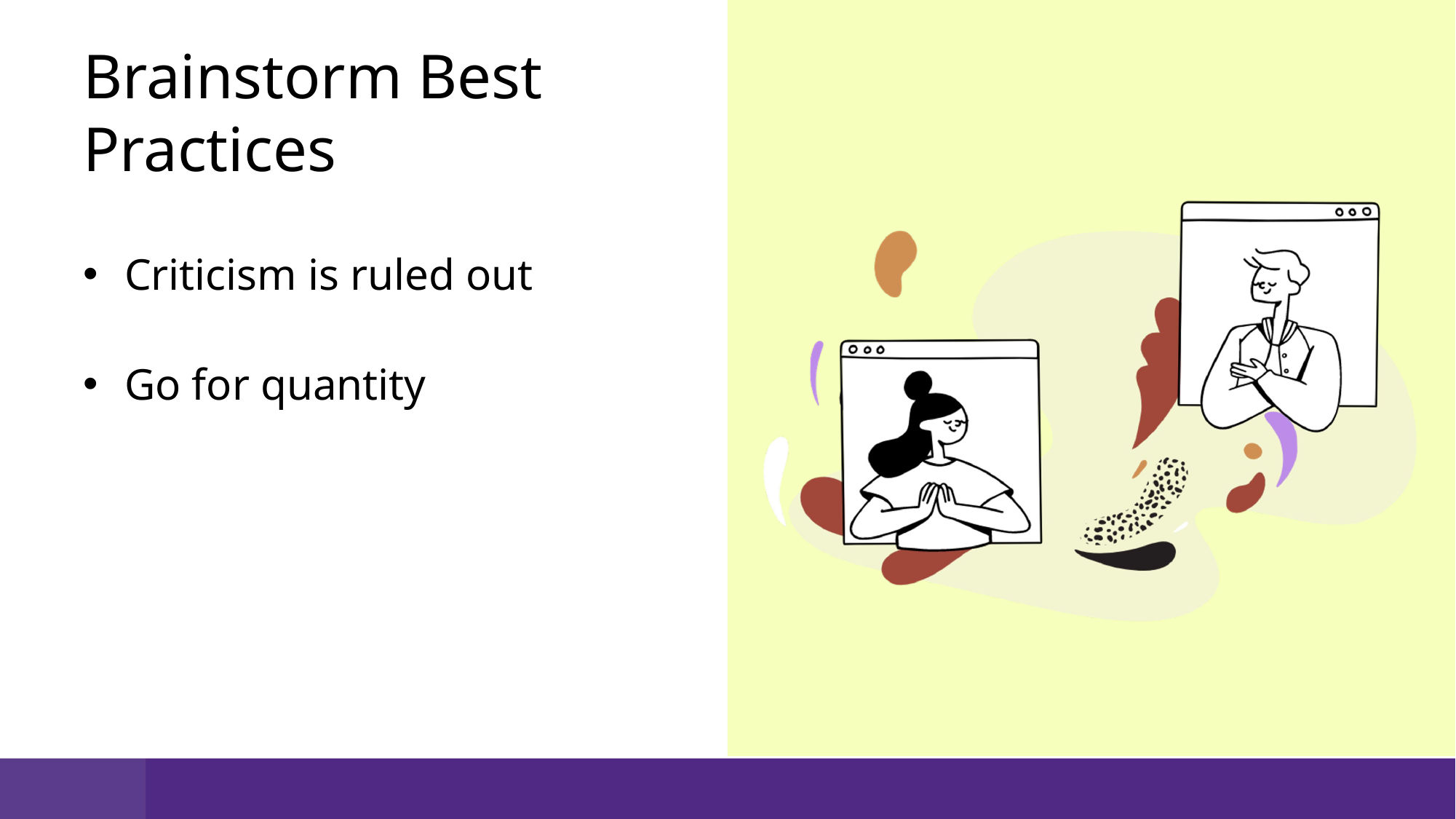

Brainstorm Best
Practices
Criticism is ruled out
Go for quantity

## Slide 40
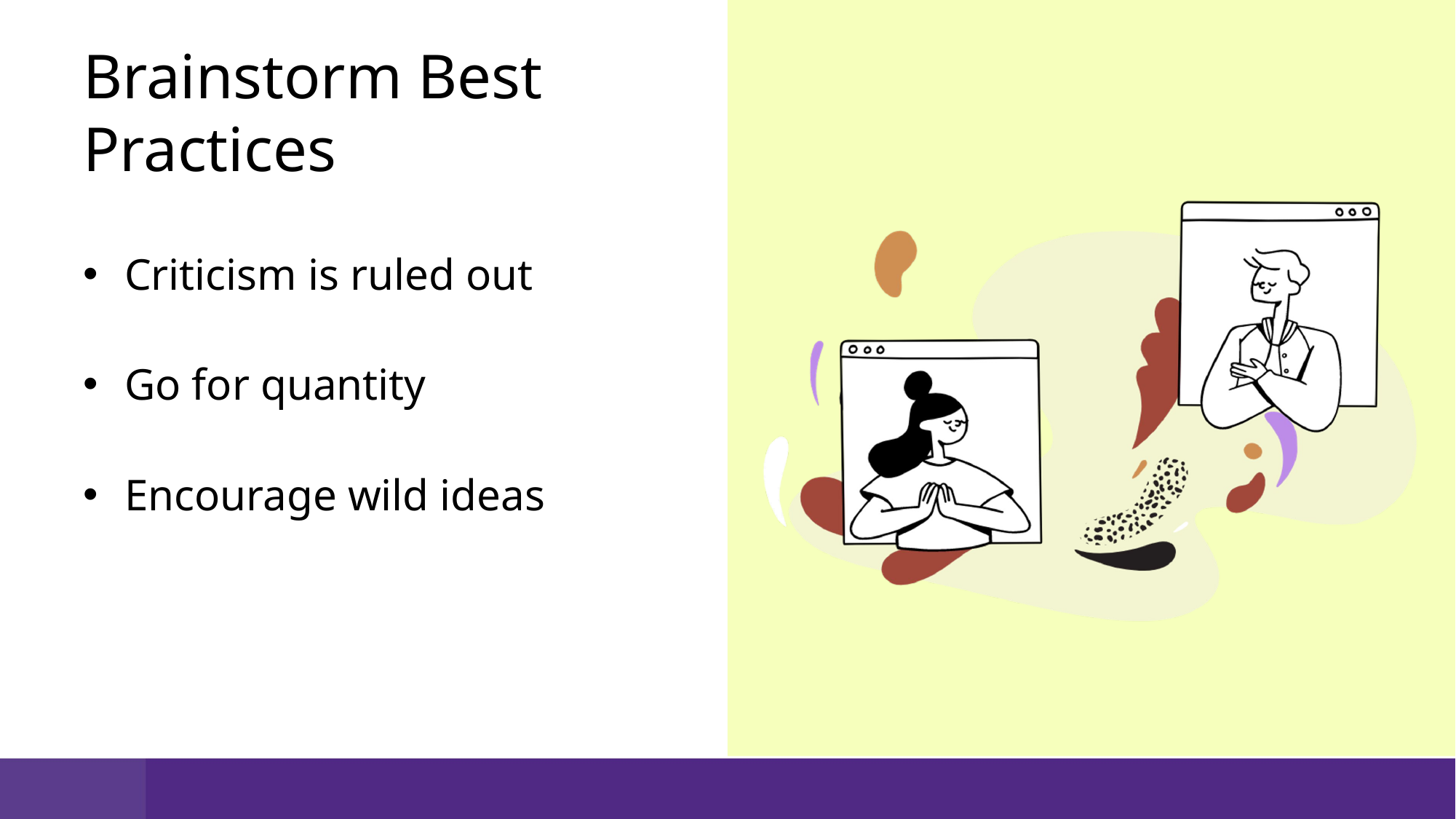

Brainstorm Best
Practices
Criticism is ruled out
Go for quantity
Encourage wild ideas

## Slide 41
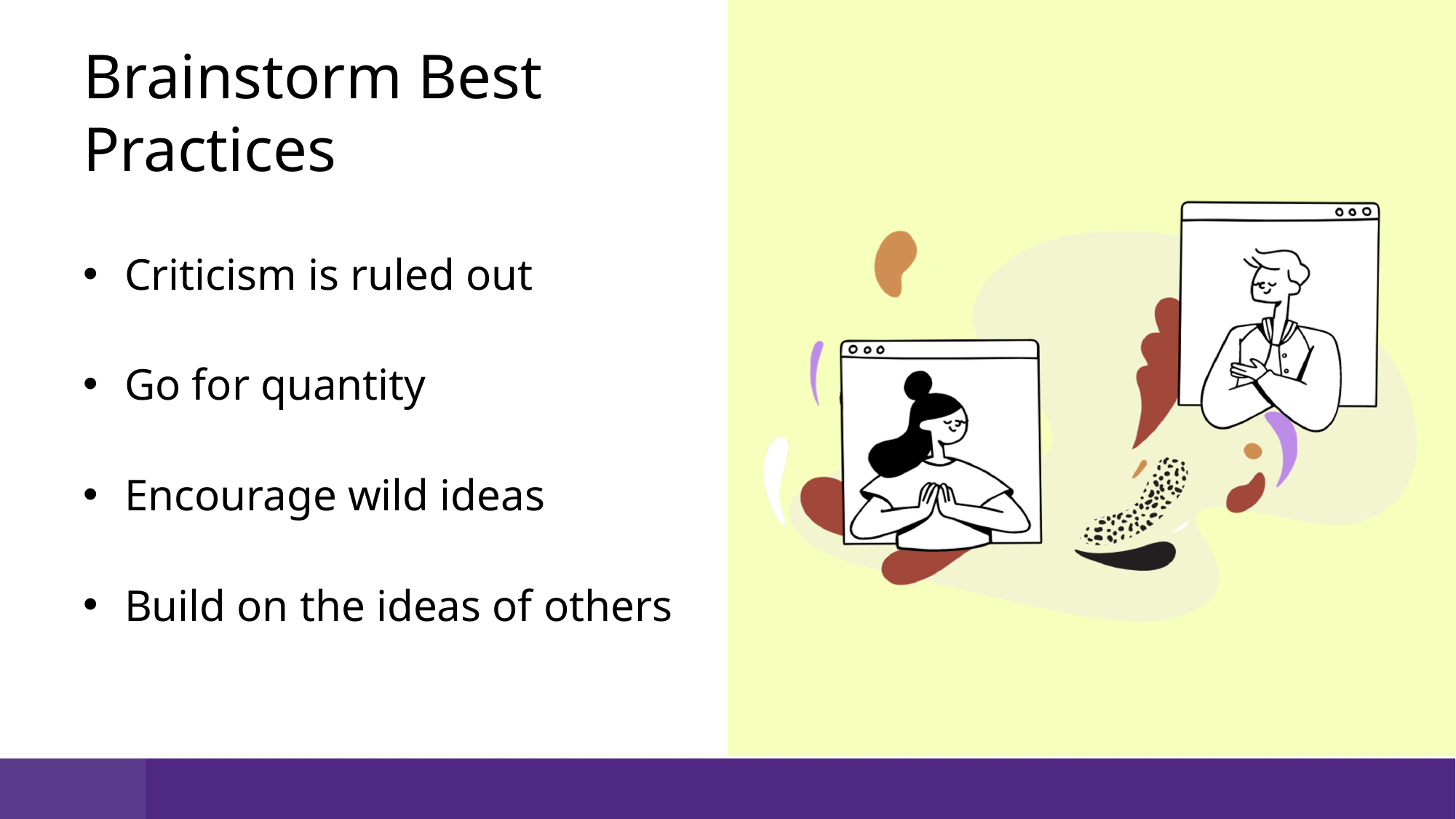

Brainstorm Best
Practices
Criticism is ruled out
Go for quantity
Encourage wild ideas
Build on the ideas of others

## Slide 42
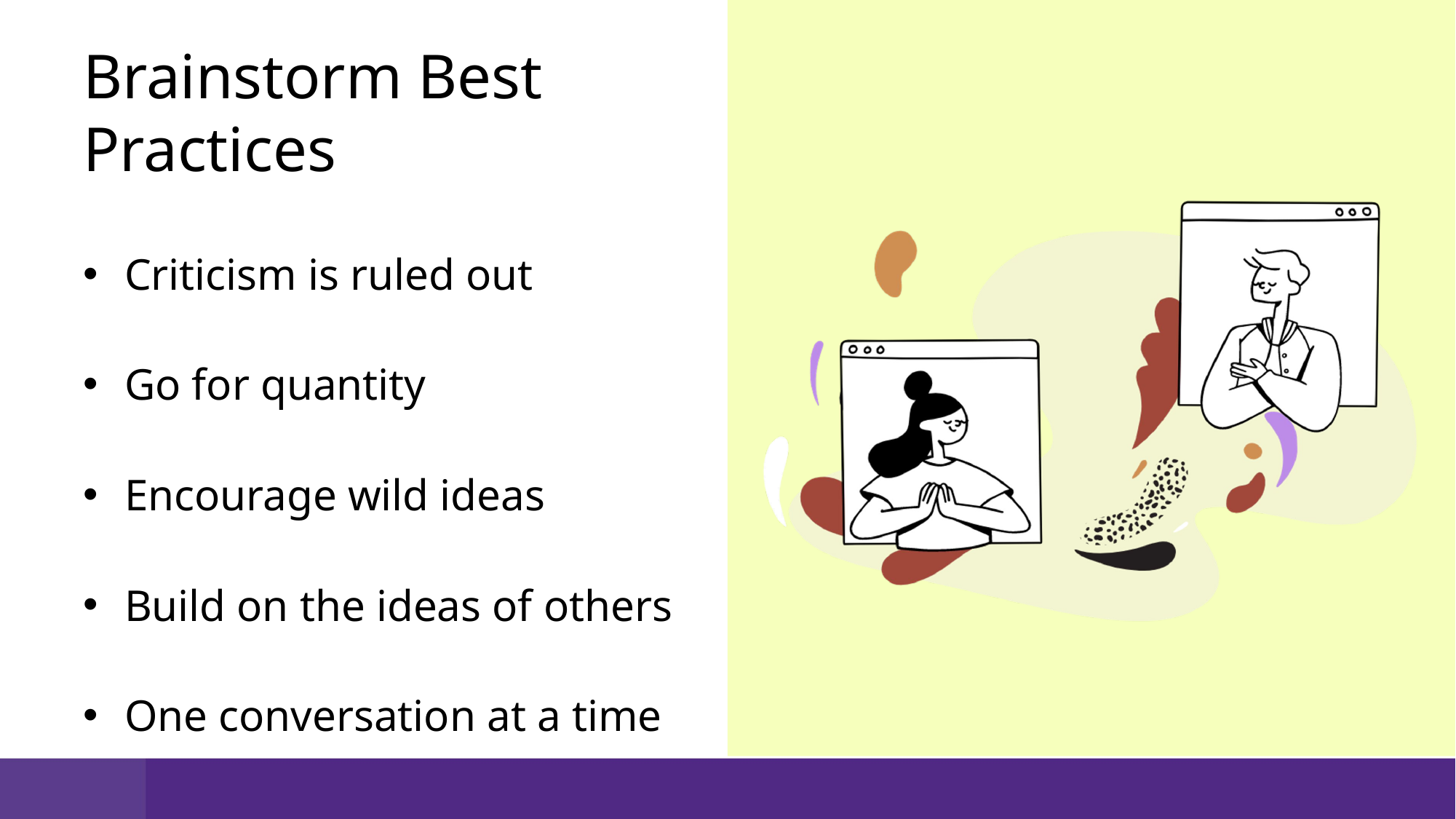

Brainstorm Best
Practices
Criticism is ruled out
Go for quantity
Encourage wild ideas
Build on the ideas of others
One conversation at a time

## Slide 43
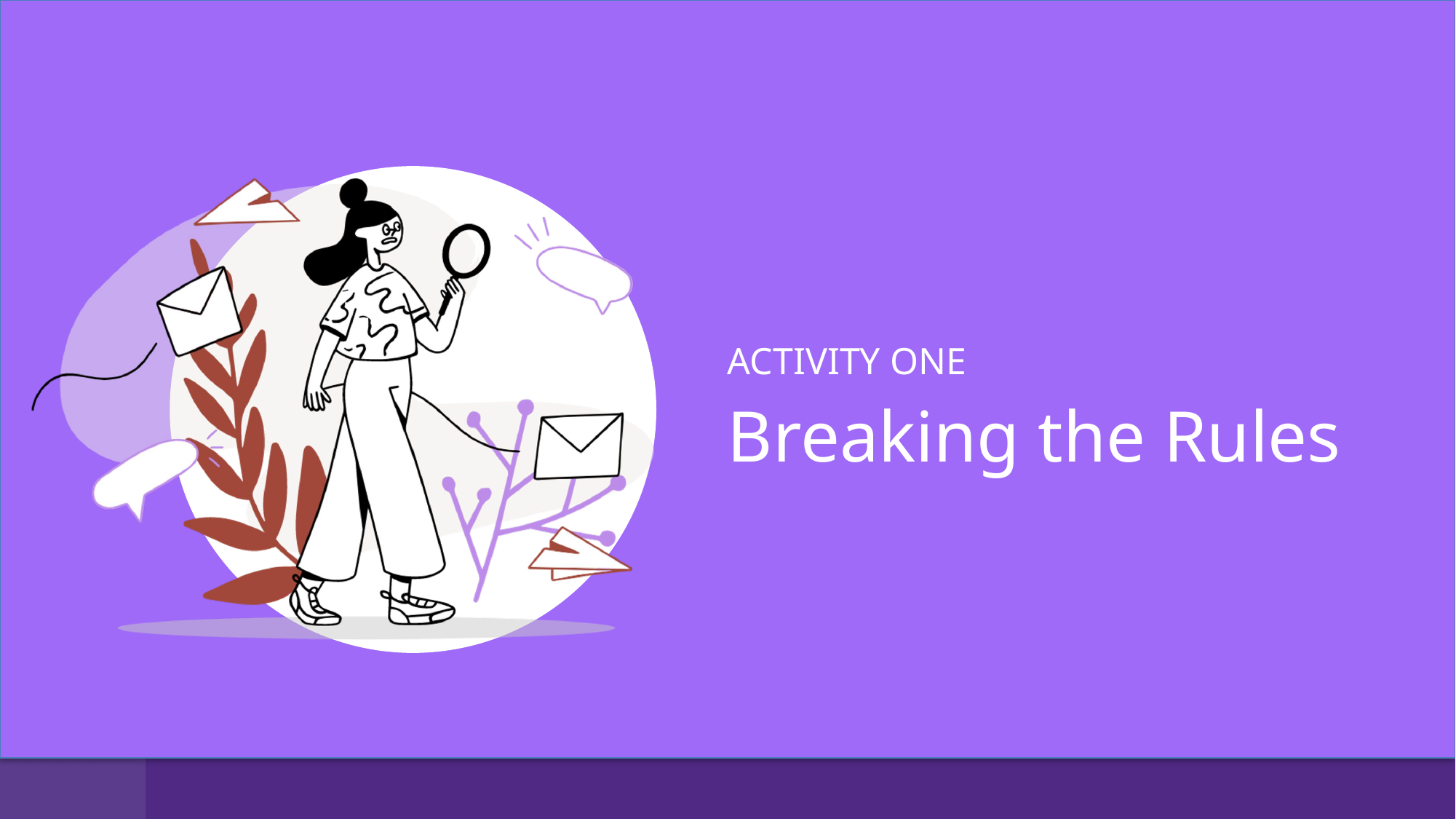

ACTIVITY ONE
Breaking the Rules

## Slide 44
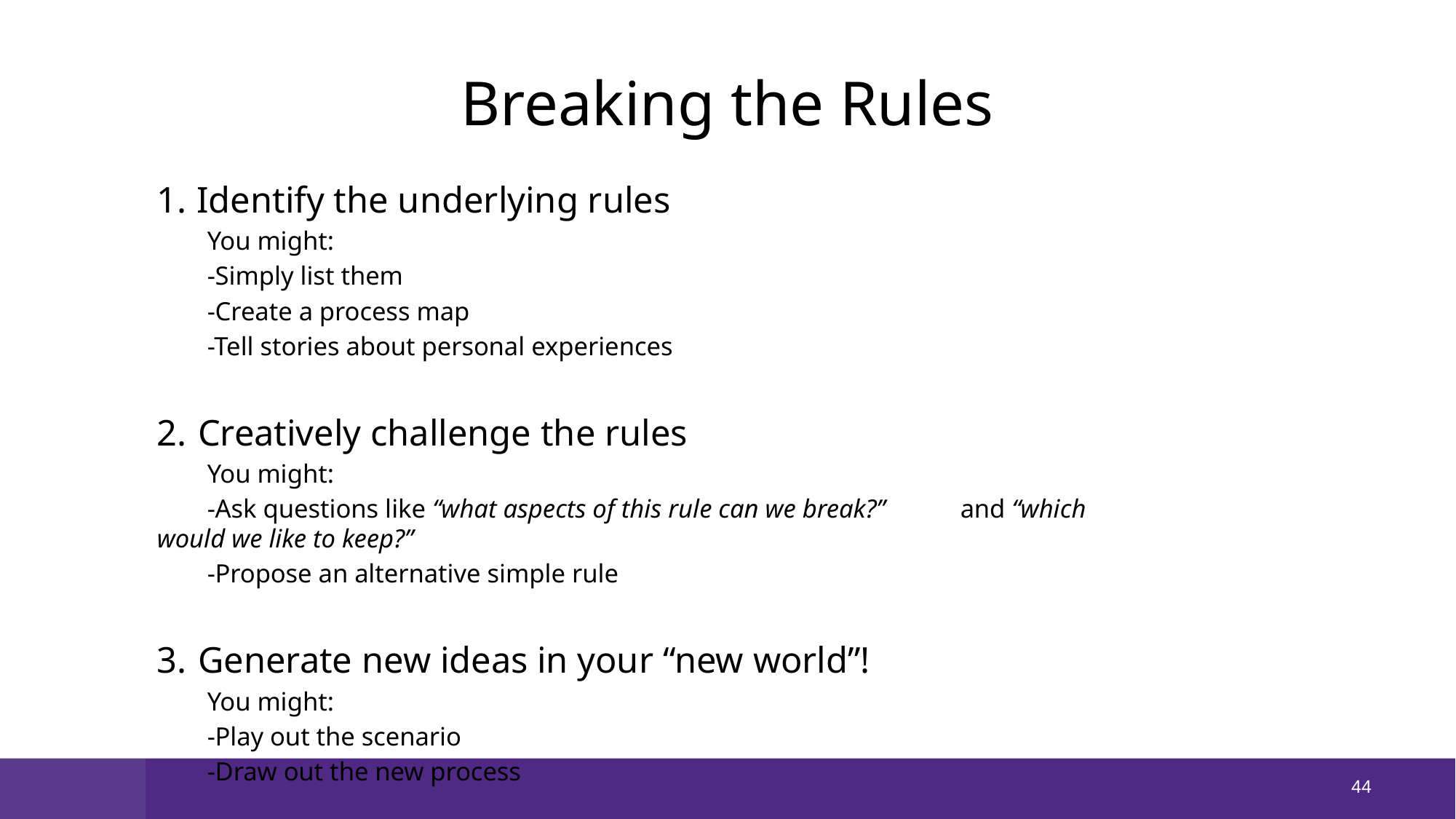

# Breaking the Rules
Identify the underlying rules
	You might:
		-Simply list them
		-Create a process map
		-Tell stories about personal experiences
Creatively challenge the rules
	You might:
		-Ask questions like “what aspects of this rule can we break?” 				and “which would we like to keep?”
		-Propose an alternative simple rule
Generate new ideas in your “new world”!
	You might:
		-Play out the scenario
		-Draw out the new process
43

## Slide 45
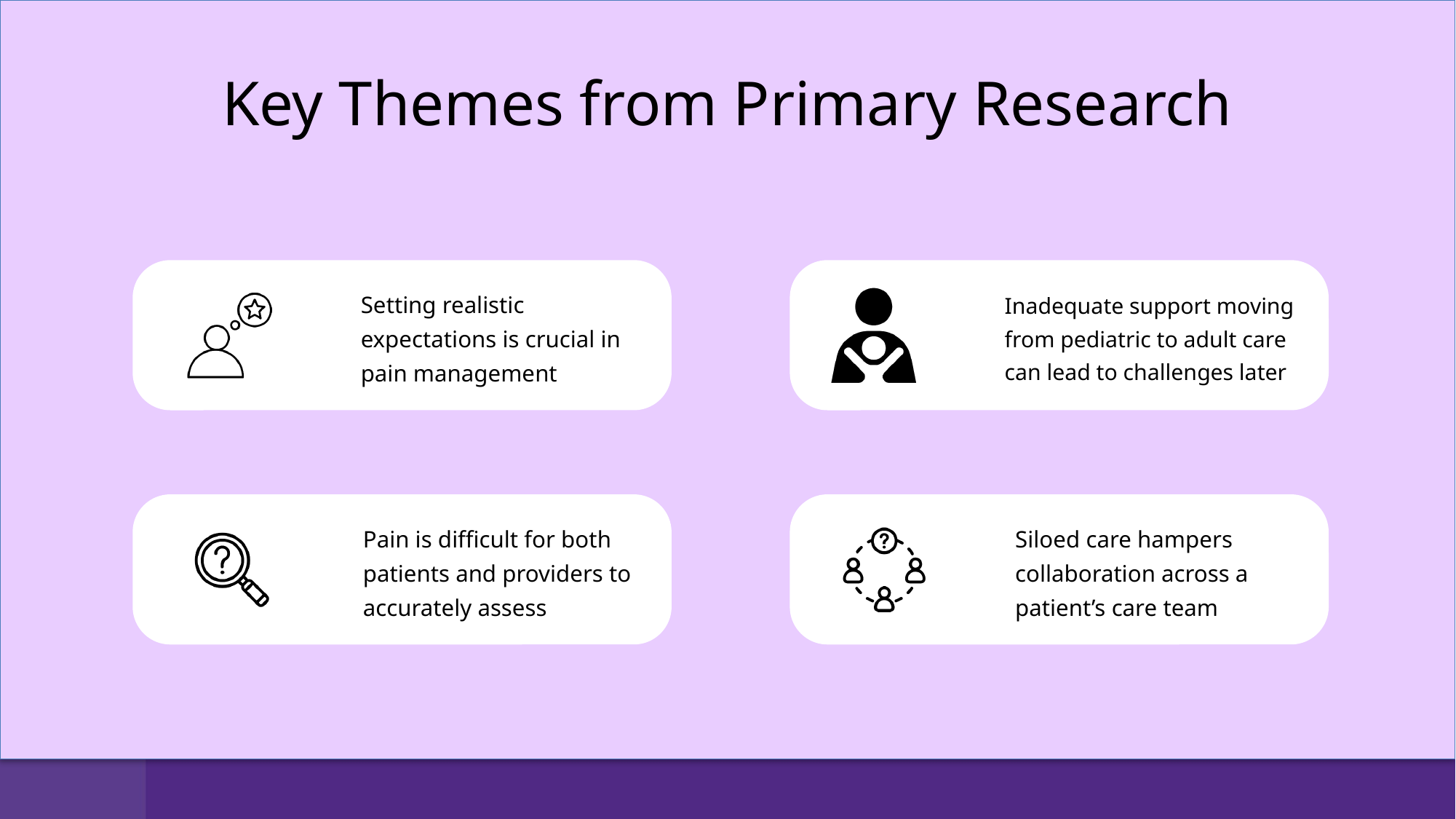

Key Themes from Primary Research
Setting realistic expectations is crucial in pain management
Inadequate support moving from pediatric to adult care can lead to challenges later
Pain is difficult for both patients and providers to accurately assess
Siloed care hampers collaboration across a patient’s care team

## Slide 46
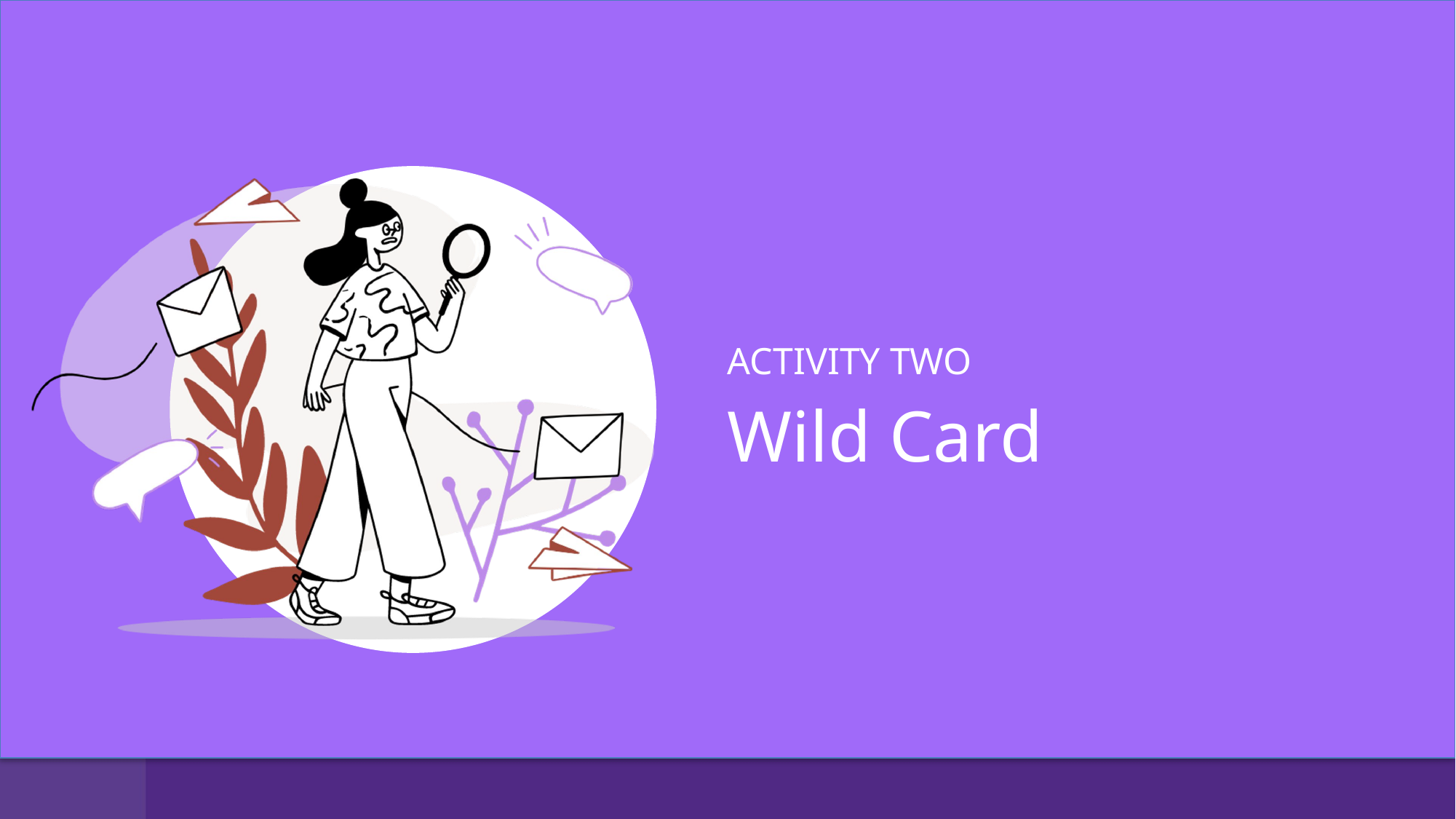

ACTIVITY TWO
Wild Card

## Slide 47
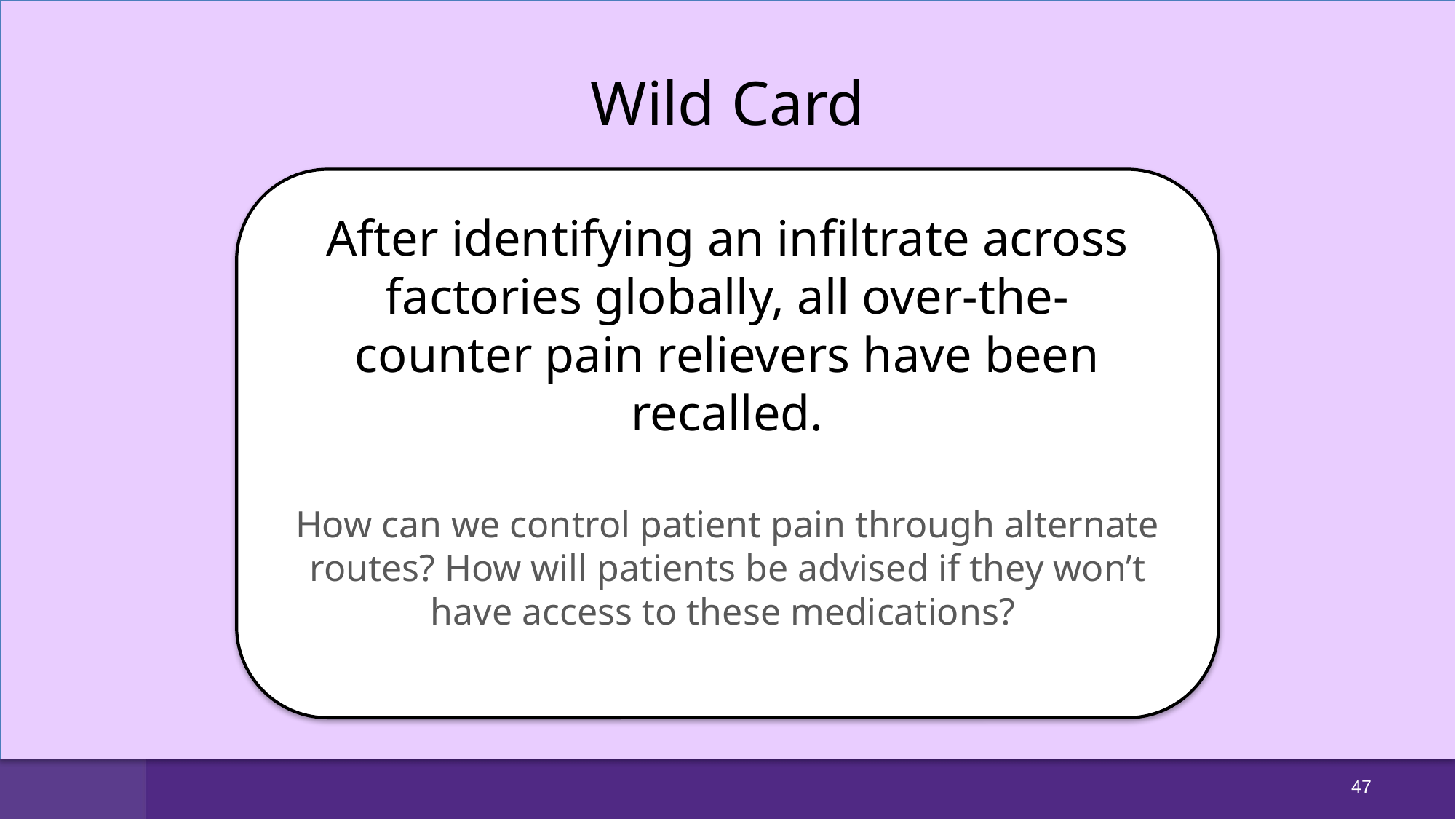

# Wild Card
After identifying an infiltrate across factories globally, all over-the-counter pain relievers have been recalled.
How can we control patient pain through alternate routes? How will patients be advised if they won’t have access to these medications?
46

## Slide 48
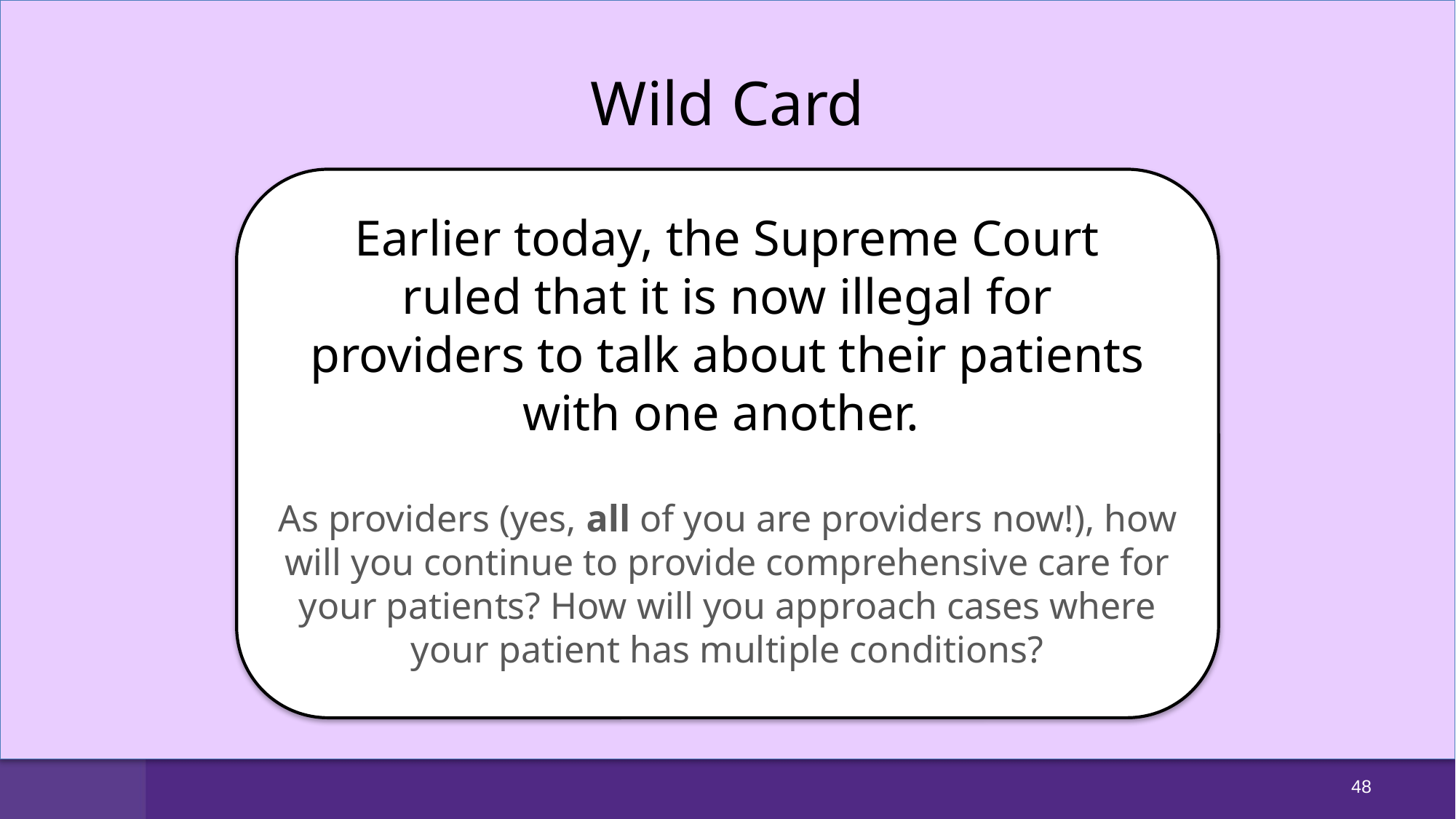

# Wild Card
Earlier today, the Supreme Court ruled that it is now illegal for providers to talk about their patients with one another.
As providers (yes, all of you are providers now!), how will you continue to provide comprehensive care for your patients? How will you approach cases where your patient has multiple conditions?
47

## Slide 49
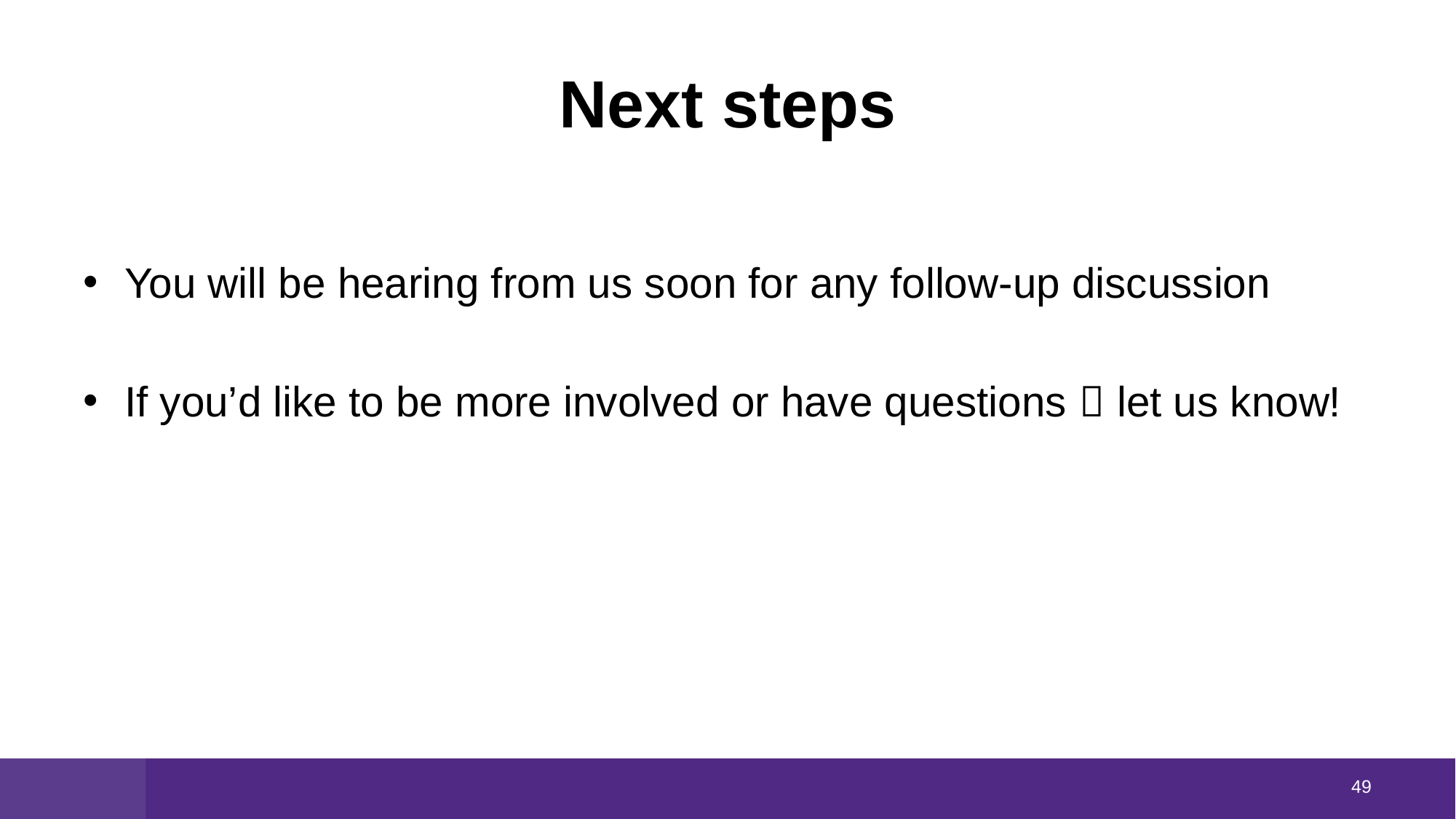

# Next steps
You will be hearing from us soon for any follow-up discussion
If you’d like to be more involved or have questions  let us know!
48

## Slide 50
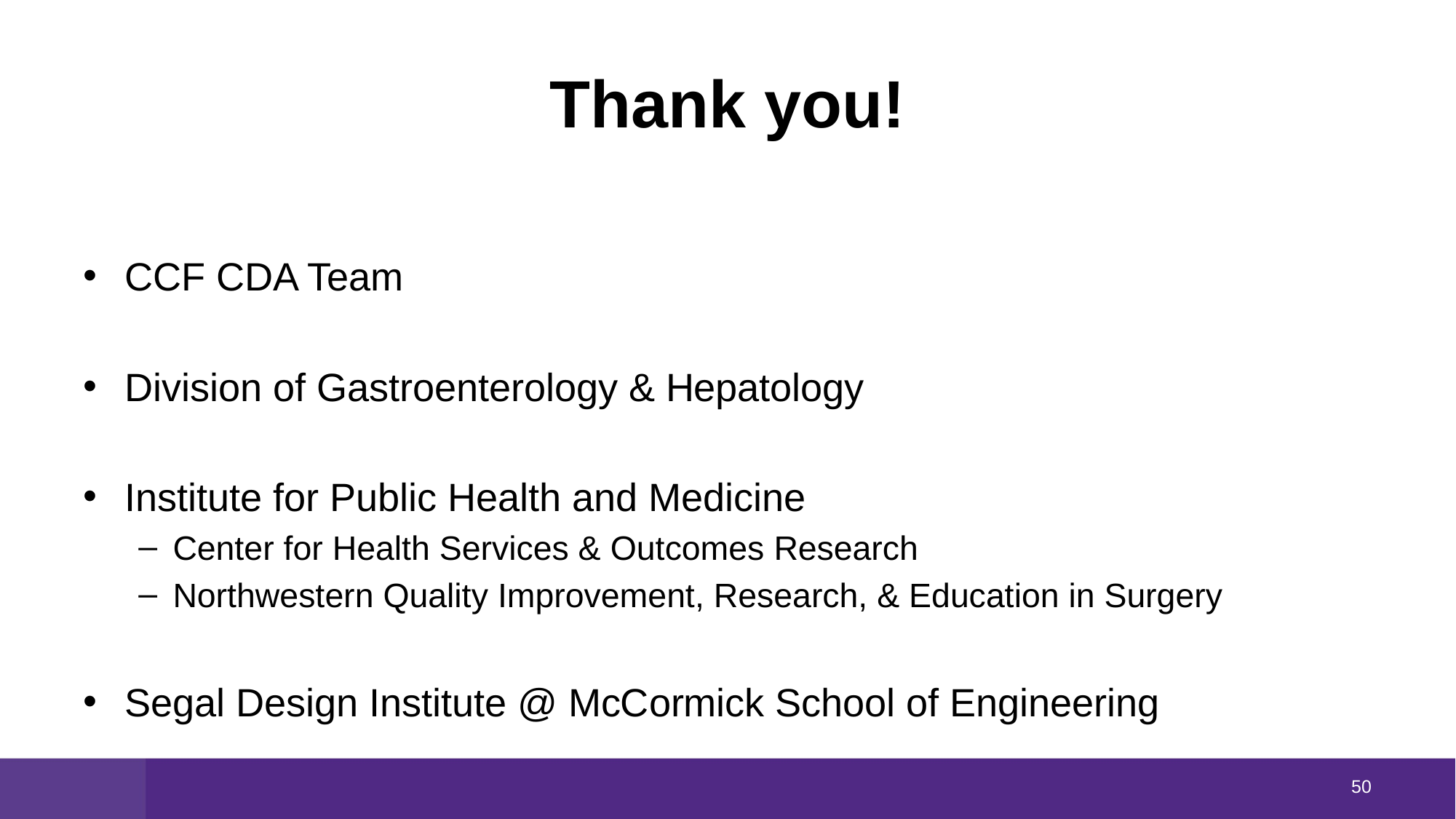

# Thank you!
CCF CDA Team
Division of Gastroenterology & Hepatology
Institute for Public Health and Medicine
Center for Health Services & Outcomes Research
Northwestern Quality Improvement, Research, & Education in Surgery
Segal Design Institute @ McCormick School of Engineering
49
