# Supplementary material for: End User Needs and Perspectives for a Digital Opioid Safety Tool in Adolescents and Young Adults With Inflammatory Bowel Disease: A Qualitative Human-Centered Design Study
Source: JMIR Form Res. 2026 Jul 31;10:e92202. doi: 10.2196/92202 (PMC13426124; doi:10.2196/92202)
Supplement: Multimedia Appendix 4 [file formative-v10-e92202-s004.docx]

**Multimedia Appendix 4: Codebook**

| **Code System** |
| --- |
| **0_Context for Table 1** |
| **Clinicians** |
| **Clinical role** |
| **Patients** |
| **IBD diagnosis** |
| **Pediatric / adult care + any care transitions** |
| **Time of diagnosis (pediatric age)** |
| **3_Great quotes to put in manuscript** |
| **1_General Care Experiences / Clinician Practices** |
| **C_Clinician practices re: pain management/opioid prescribing** |
| **Patient experiences with pain/opioids (if any)** |
| **Staying healthy is a team sport** |
| **Family, social, and financial support** |
| **Communication with clinical team is key** |
| **MyChart for example** |
| **Patients have to be informed/take active role in own care** |
| **There is a need for patient autonomy over own care** |
| **Experience of growing up as an IBD patient** |
| **Challenges finding the right GI doc** |
| **Lots of self-management** |
| **Challenges with medications** |
| **2_Expectations for Pain Management + Opioid Safety Intervention** |
| **Within clinical teams/health systems** |
| **C_Acknowledge opioid safety is not just 1 clinician's job** |
| **From co-design session** |
| **C_Acknowledge that most clinicians not equipped to manage pain** |
| **C_Account for clinical workflows / limited time** |
| **C_Fill information gap efficiently around individual risk** |
| **C_Account for pain management outside the IBD clinic** |
| **In General** |
| **C_Support AYA as they transition to independence** |
| **From co-design session** |
| **C_Frame conversation around overall health + wellness** |
| **C_Acknowledge that every patient is different** |
| **From co-design session** |
| **Patient-Level** |
| **Acknowledge caregiver views if needed** |
| **C_Connect patients with effective/safe non-opioid meds** |
| **From co-design session** |
| **Especially postoperatively** |
| **C_Consider marijuana or other alternative approaches** |
| **C_Acknowledge / assess pain experience and/or set expectations** |
| **From co-design session** |
| **C_Consistently educate patients** |
| **From co-design session** |
